# Evaluation of pathogen specific urinary peptides in tick-borne illnesses

**DOI:** 10.1038/s41598-020-75051-3

**Published:** 2020-11-09

**Authors:** Ruben Magni, Raghad Almofee, Sameen Yusuf, Claudius Mueller, Ngoc Vuong, Mahmood Almosuli, Minh Thu Hoang, Katherine Meade, Ish Sethi, Nuha Mohammed, Robyn Araujo, Teresa Kaza McDonald, Paul Marcelli, Virginia Espina, Brianna Kim, Anja Garritsen, Christine Green, Paul Russo, Weidong Zhou, Iosif Vaisman, Emanuel F. Petricoin, Deborah Hoadley, Robert E. Molestina, Hope McIntyre, Lance A. Liotta, Alessandra Luchini

**Affiliations:** 1 Center for Applied Proteomics and Molecular Medicine, George Mason University, Manassas, VA 20110 USA; 2 Queensland University of Technology, Brisbane, Australia; 3 Ceres Nanosciences, Manassas, USA; 4 Innatoss, Oss, The Netherlands; 5 Medical Offices of Christine Green, Mountain View, USA; 6 New England Institute for Lyme Disease and Tick-Borne Illness, Longmeadow, USA; 7 American Type Culture Collection, Manassas, USA; 8 Lyme Hope, LLC, Mt Airy, USA

**Keywords:** Mass spectrometry, Biomarkers, Infectious diseases

## Abstract

Mass spectrometry enhanced by nanotechnology can achieve previously unattainable sensitivity for characterizing urinary pathogen-derived peptides. We utilized mass spectrometry enhanced by affinity hydrogel particles (analytical sensitivity = 2.5 pg/mL) to study tick pathogen-specific proteins shed in the urine of patients with (1) erythema migrans rash and acute symptoms, (2) post treatment Lyme disease syndrome (PTLDS), and (3) clinical suspicion of tick-borne illnesses (TBI). Targeted pathogens were Borrelia, Babesia, Anaplasma, Rickettsia, Ehrlichia, Bartonella, Francisella, Powassan virus, tick-borne encephalitis virus, and Colorado tick fever virus. Specificity was defined by 100% amino acid sequence identity with tick-borne pathogen proteins, evolutionary taxonomic verification for related pathogens, and no identity with human or other organisms. Using a cut off of two pathogen peptides, 9/10 acute Lyme Borreliosis patients resulted positive, while we identified zero false positive in 250 controls. Two or more pathogen peptides were identified in 40% of samples from PTLDS and TBI patients (categories 2 and 3 above, n = 59/148). Collectively, 279 distinct unique tick-borne pathogen derived peptides were identified. The number of pathogen specific peptides was directly correlated with presence or absence of symptoms reported by patients (ordinal regression pseudo-R^2^ = 0.392, p = 0.010). Enhanced mass spectrometry is a new tool for studying tick-borne pathogen infections.

## Introduction

With an estimate of 300,000^1–3^ cases per year in the US, Lyme borreliosis is the most common vector-borne infection in North America^1,2,4^. Despite the incidence of tick-borne infections and the enormous suffering they cause, progress in accurate diagnosis and durable treatment regimens^5–7^ has been greatly hindered by questions surrounding: (a) the cause of persistent post-treatment Lyme symptoms^4,8,9^, and (b) the prevalence and medical significance of coinfections by two or more tick-borne pathogens^10–12^. The first goal of this study is to introduce a new experimental and bioinformatic authentication method for characterizing the urinary pathogen-derived proteome. The second goal is to apply this technology to acute phase Borreliosis patients and patients with clinical suspicion of tick-borne illnesses.

Post Treatment Lyme Disease Syndrome (PTLDS) defines a subset of patients who continue to experience a variety of symptoms including fatigue, muscoloskeletal pain or cognitive impairment along with functional impairement^4,13^ following antibiotic therapy for Lyme disease. The cause of the persistent symptoms in PTLDS is unknown ^4,6,14^. Direct molecular evidence is lacking to verify that the symptoms are caused by persistence of an active tick-borne pathogen infection^15^. Symptom persistence has been attributed either to immunologic and inflammatory phenomena that are triggered after a successfully treated infection, or to illnesses not associated with a tick-borne infection^16^. Recent molecular evidence suggested that post treatment persistence of Lyme arthritis symptoms maybe influenced by the persistence of Borrelia peptidoglycans in synovial fluid^17^. Whether these biomolecules are derived from viable pathogens or persist in the body long after the infection has resolved remains to be determined.

**Figure 1:**
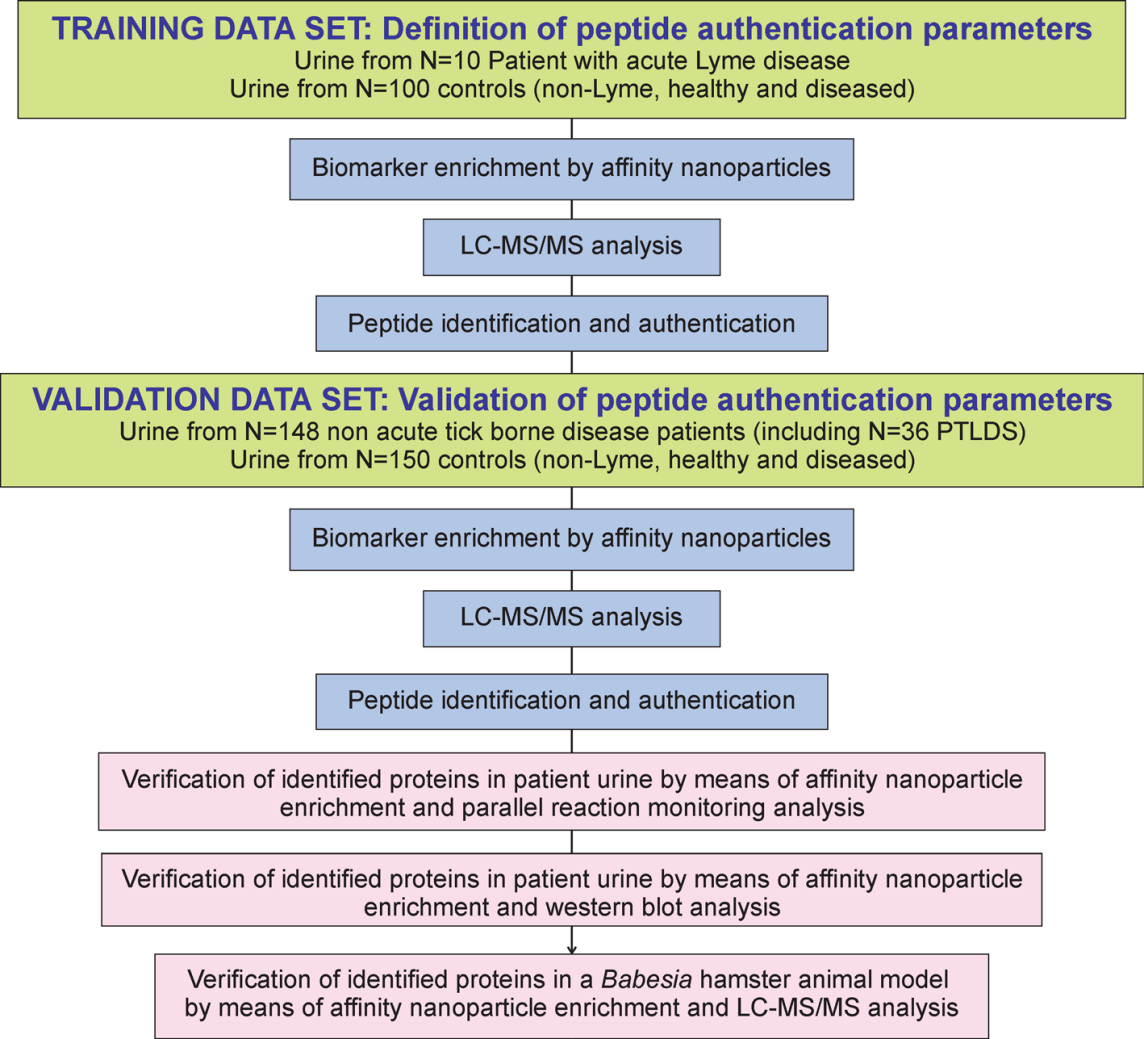
Experimental design for pathogen derived, urinary peptide identification and authentication. Experimental analysis and bioinformatics pipeline was applied to a first set of n = 110 individuals, n = 10 acute Lyme borreliosis patients according to CDC criteria and n = 100 controls, comprising healthy and diseased non-Lyme participants. Parameters for peptide identification and authentications were established in this first phase. The method was verified in an independent set of n = 298 participants, including n = 148 non acute patients suspected of tick-borne illness and n = 150 healthy and diseased controls. Urinary peptides were validated by means of orthogonal methods including western blot analysis, parallel reaction monitoring, and a Babesia animal model. *PTLDS *post treatment Lyme disease syndrome.

**Figure 2:**
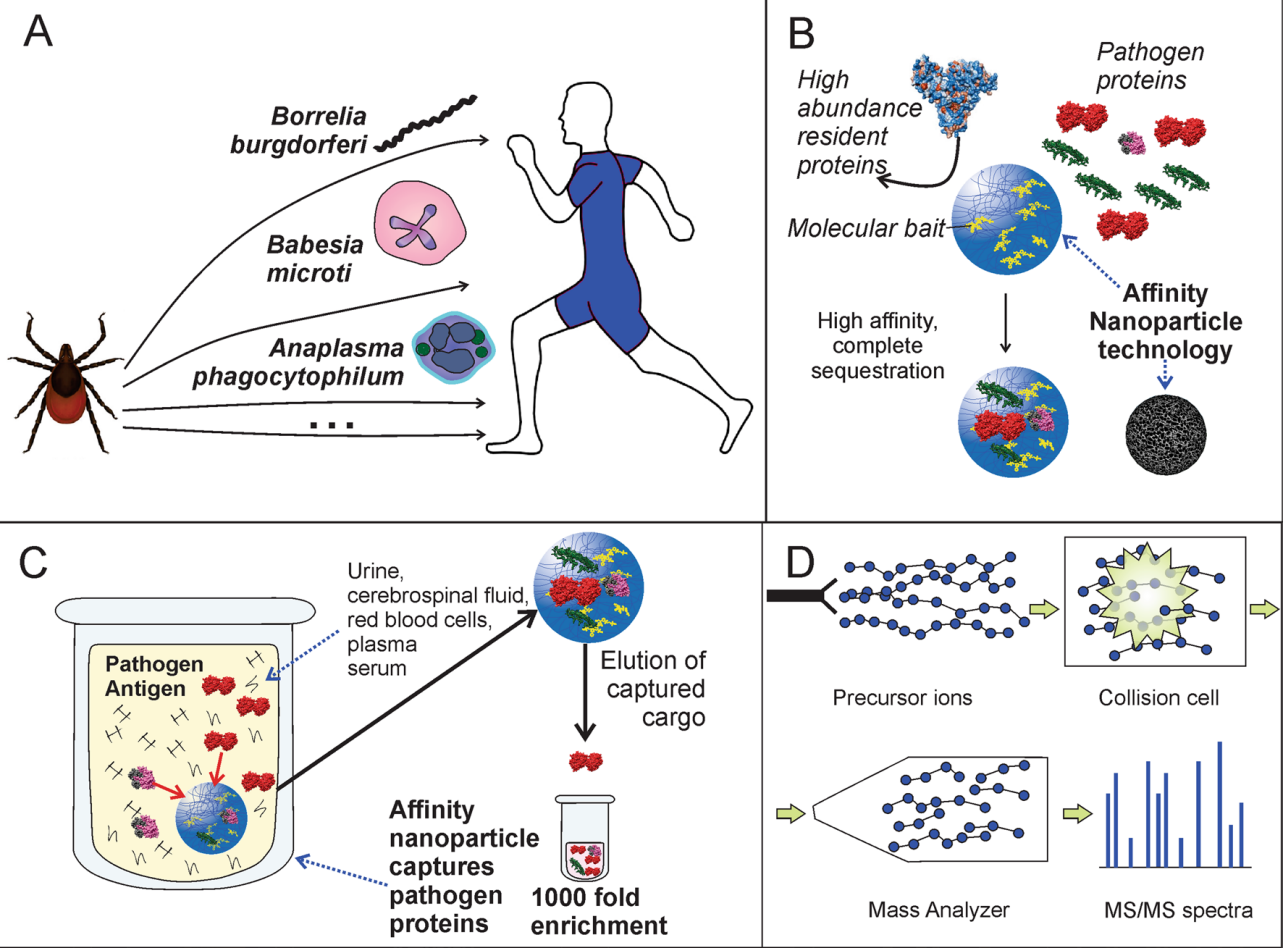
Experimental workflow for tick-borne pathogen derived peptide identification. (**A**) Urine samples were collected under informed consent; (**B**,**C**) urine was subjected to pre-analytical concentration using affinity particles, (**D**) affinity particle extracts were digested with trypsin and analyzed by means of non-targeted LC–MS/MS. This figure was prepared using CorelDRAW X7.

Addressing this need, the present study uses hydrogel particle-enhanced mass spectrometry to characterize pathogen specific proteins in body fluids such as urine (Figs. 1 and 2). The method used herein has been used previously to detect non-tick-borne active infections for a variety of pathogens including HIV-negative tuberculosis^18^, Chagas disease^19^, and Toxoplasmosis^20^ with high sensitivity and specificity. Patients enrolled in this study fall into five categories: (1) patients with acute stage *Borrelia* infection defined by a two-tier serology criteria; (2) symptomatic patients with a clinical diagnosis of PTLDS^4,13^; (3) patients treated in community centers and private practices with clinical suspicion of tick-borne illnesses but in the absence of complete clinical information regarding previous symptoms and treatments; (4) diseased controls, which include patients harboring non-tick-borne infections, who are hospitalized in Peru, a geographic region where ticks are very rare, and U.S. patients with a diagnosis of traumatic brain injury and acute respiratory distress syndrome; and (5) healthy controls. Specificity of the mass spectrometry analytic method is ensured by a three-tier authentication algorithm which requires stringent filters for peptide identification, 100% amino acid sequence identity with tick-borne pathogen proteins, evolutionary taxonomic verification for related pathogens, and lack of identity with human or non-tick-borne pathogenic organisms (Fig. S1). Identified peptides are verified by concomitant urine western blot immunoassays, orthogonal mass spectrometry based parallel reaction monitoring (PRM), and an animal model of persistent Babesiosis. The parameters for the authentication algorithm are established on a set of acute Lyme patients and non-Lyme controls; the method is then applied to a non-overlapping set of non acute patients including PTLDS (category 2) and other patients suspected of tick-borne illnesses (category 3), and controls. The correlation between the number of pathogen-specific urinary peptides and the presence or absence of symptoms as assessed by health care professionals is investigated.

## Results

### Detection of *Borrelia* specific urinary peptides: training set

Affinity particle/mass spectrometry workflow was highly reproducible and analytical sensititity was 2.5 pg/mL in urine from healthy controls spiked with *Borrelia* lysate (Supplementary Results, Table S1, Fig. S2). In the training phase of the study, we analyzed urinary pathogen peptides in N = 10 patients suffering from early stage Lyme borreliosis and diagnosed according to CDC guidelines and 100 non-Lyme controls (Wilcoxon–Mann–Whitney p-value = 2.2E−16, power = 0.999, alpha = 0.05, Fig. S3). Patients (M/F = 0.66, median age = 46.5, IQR = 11.75; female = 6, median age = 56.5, IQR = 4.25, Table 1) presented with a characteristic *Erythema migrans* (EM) rash as well as concurrent symptoms such as fever, joint pain, malaise and neurological symptoms (Table S2). The urine was collected pre-treatment for all patients. All 10 acute patients were later verified to be two tier serology positive 3–6 weeks later. The control group consisted of 100 patients (M/F = 1, median age = 41, IQR = 25.75), including healthy controls and disease control patients diagnosed with acute respiratory distress syndrome, tuberculosis, and traumatic brain injury (Table S3). Peptides derived from *Borrelia* species known to be pathogenic in humans were found in 10/10 Lyme borreliosis patients, and two or more peptides were found in 9/10 samples. No peptides derived from other tick borne organism investigated (*Babesia, Anaplasma, Rickettsia, Ehrlichia, Bartonella, Francisella*, Powassan virus, tick borne encephalitis virus, and Colorado tick fever virus) were identified. The list of peptides identified in each patient is reported in Table 2, Tables S4, S5. Identified peptides belonged to different proteins including membrane associated proteins (e.g. OspC), motility proteins (e.g. flagellar motor switch protein FliN), transport proteins (e.g., ABC transporter substrate binding protein, periplasmic oligopeptide binding protein, mechanosensitive ion channel family protein), chemotaxis proteins (e.g. MotA), protein translation and modification proteins (e.g., peptide chain release factor N(5), glutamine methyl transferase), metabolic enzymes (e.g. Chain A, Glyceraldehyde-3-phosphate dehydrogenase, nicotinate phosphoribosyltransferase), RNA and protein metabolism (e.g. translation elongation factor YigZ), and antigens known to elicit immune response (e.g., immunogenic protein P37). A patient was considered positive for a given pathogen if at least two unique peptides deriving unambiguously (100% sequence identity with the pathogen and less than 90% sequence identity with any other organism) from such pathogen were identified. Zero false positive peptides were identified in 100 healthy and diseased controls (Fig. S4).

**Table 1:** Demographic information of patients enrolled in this study.

Characteristics	Patients			Controls	
	Acute LB	PTLDS	NA-TBI	Diseased controls	Healthy controls
N	10	36	112	215	35
Age (median)	52.5 (IQR = 14)	50 (IQR = 25)	46 (IQR = 32)	35.5 (IQR = 31)	31 (IQR = 60)
**Sex**					
Male/female	4/6	14/22	54/58	112/57	18/17
NA	–	–	–	46	–
Tick discovered	6Y/ N	11Y/10N	11Y/15N	–	–
Erythema migrans rash	10Y/0N	26Y/5N^a^	24N^a^	–	–
Muscoskeletal pain	7Y/0N	22Y/4N	15Y/N	–	–
Fatigue	7Y/0N	18Y/6N	20Y/4N	–	–
Fever	7Y/0N	11Y/13^a^	7Y/17N^a^		
Facial palsy	1Y/3N	3Y/20N^a^	3Y/21N^a^	–	–
Cognitive impairment	4Y/0N	16Y/7N	9Y/14N	–	–
Serology (Pos)	10Y/0N^b^	19Y/4N	12Y/14N		

Demographic and clinical characteristics of sample cohort.

*Acute LB *acute Lyme Borreliosis, *PTLDS *post treatment Lyme disease syndrome, *NA-TBI *suspected non-acute tick borne infection.

^a^A history of Erythema migrans, fever, and facial palsy were recorded for PTLDS and TBI patients.

^b^The urine was collected before treatment regimen for all patients. All 10 acute patients were verified to be two tier serology positive 3–6 weeks later.

**Table 2:** Borrelia peptides identified in the urine of acute Lyme borreliosis cases.

Sample ID	EM rash	Acute LB symptoms	Serology	Under treatment (at time of collection)	Number of identified Borrelia peptides	Peptide sequence
108838	Y	Y	Pos	No	3	IDTEEAAVK; NAGNFEIIR; VTLSDISTK
104821	Y	Y	Pos	No	2	AILKTNGDKTLGAAELEK; NNFCLSKSDLEEIR
790907	Y	Y	Pos	No	2	SNQDNQTLLLSLHQAIAK; LKNSHAELGVAGNGATTDENAQK
889597	Y	Y	Pos	Yes (2 days doxycicline)	2	GGYLDHVNHAGAKKVILTVPAK LATVNEAEGK
213567	Y	Y	Pos	No	2	LATVNEAEGK; NDVSEEKPEIK
453742	Y^a^	Y^a^	Pos	No	3	VVILNEATGK; LATVNEAEGK; FVYIGNVDNMGYTINFK;
463256	Y	N	Pos	No	2	NLSLFTDFYEISMMNAYFIK; QKATGAINAVSGEQIL
459235	Y	Y	Pos	Yes (1 day doxycicline)	2	SAKEVINNTSTMEK; SSSVDGFVSFKEYKER
729340	Y	Y	Pos	No	1	FEDAIVLRDK
310741	Y	Y	Pos	No	2	VNESDLGIKALDEK; FNVEACFPTLIVEK

The urine was collected before treatment regimen for all patients. All 10 acute patients were verified to be two tier serology positive 3–6 weeks later. Acute Lyme Borreliosis symptoms = joint pain, fatigue, facial palsy, neurological symptoms.

^a^Developed after collection.

### Tick-borne pathogen peptides are present in the urine of 40% of nonacute patients with clinical suspicion of tick-borne illnesses

**Figure 3:**
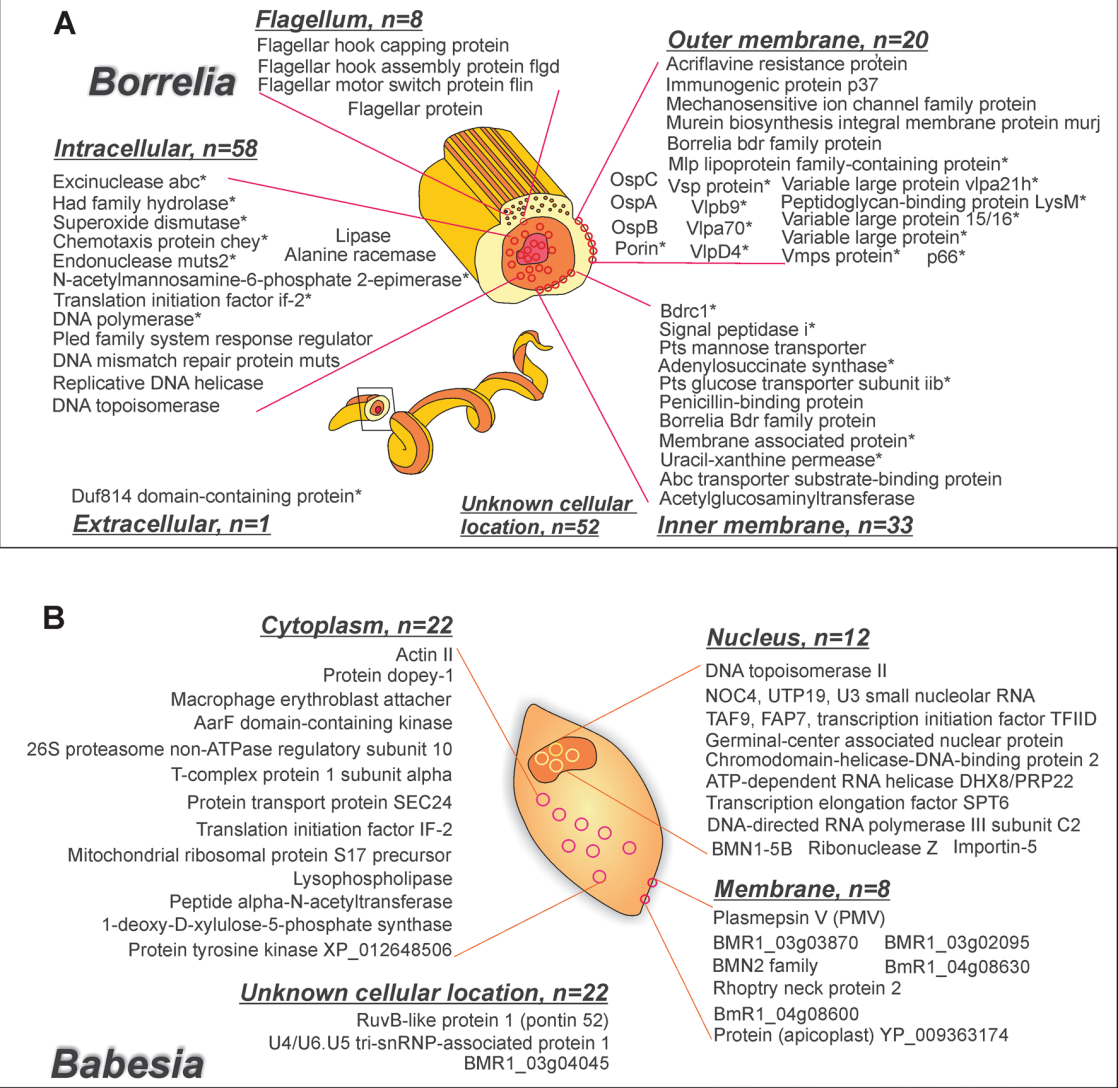
Borrelia and Babesia peptides. (**A**) Borrelia peptides (160) identified in patients affected by acute Lyme borreliosis, post treatment Lyme disease syndrome or suspected of tick-borne illnesses. Proteins marked with a * were unambiguously (100% sequence identity with the pathogen with less than 90% homology with human and other organisms) attributed to the following species: *Borrelia* *hermsii*, *Borrelia* *turicatae*, *Borrelia* *duttonii*, *Borrelia* *miyamotoi*, *Borrelia* *recurrentis*. Unmarked proteins were attributed to the following species: *Borrelia* *burgdorferi*, *Borrelia* *mayonii*, *Borrelia* *garinii*, *Borrelia* *afzelii*, *Borrelia* *bavariensis*, *Borrelia* *spielmani*. (**B**) Babesia peptides (n = 62) identified in patients diagnosed with post treatment Lyme disease syndrome. 37/148 non acute patients had at least one Babesia urinary peptide, and 17/148 non acute patients had two or more peptides. This figure was prepared using CorelDRAW X7.

**Figure 4:**
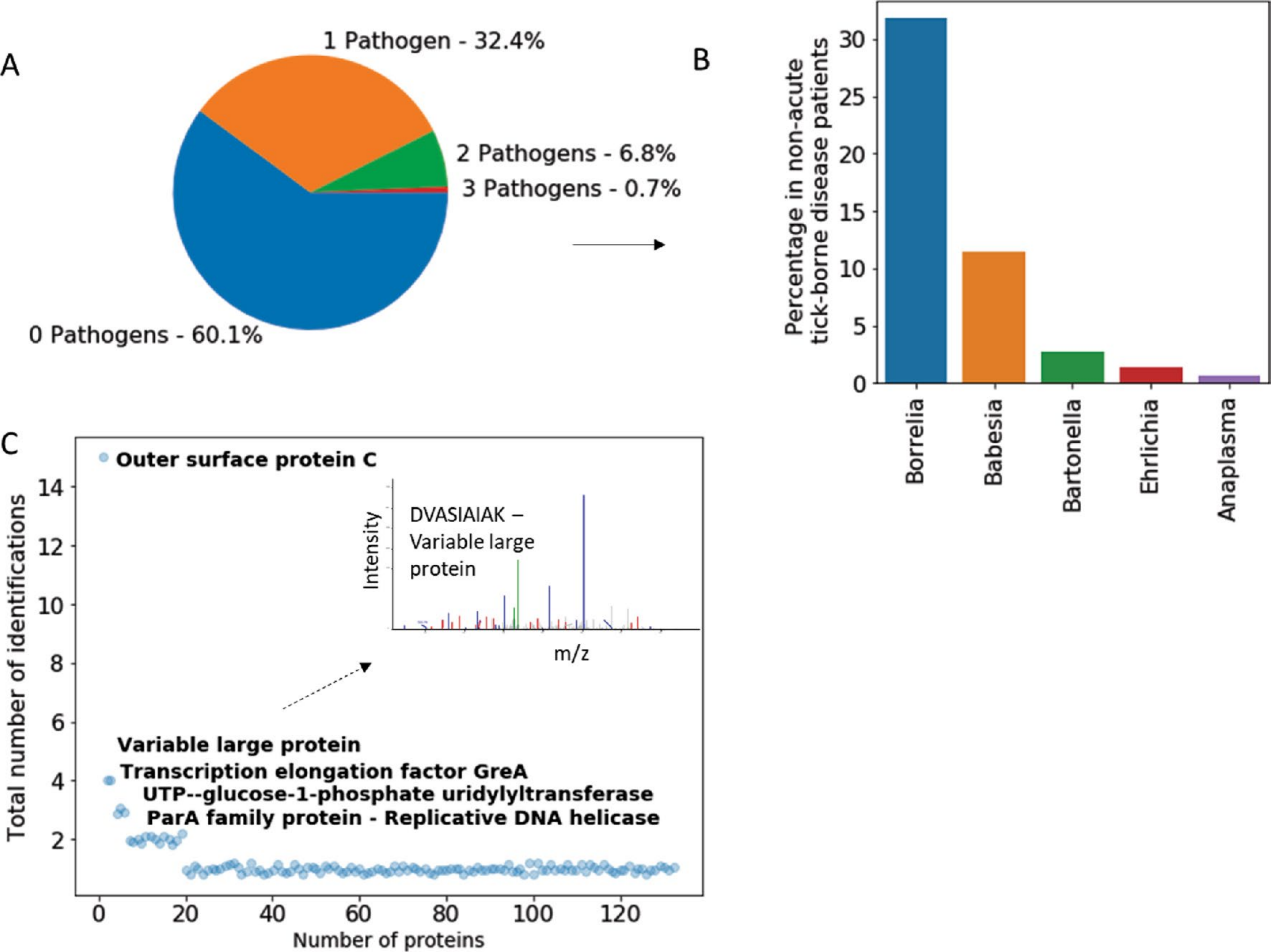
Pathogen and peptide distribution. (**A**) Pathogen peptides were found in the urine of 40% of non-acute tick borne disease patients (cut off > 2 peptides per organism). 32% of patients had urinary peptides deriving from 1 pathogen. 7% of patients presented peptides from two pathogens and less than 1% from three pathogens. (**B**) *Borrelia* peptides were found in 48/148 patients, *Babesia* peptides in 17/148, *Ehrlichia* peptides in in 2/148, *Anaplasma* peptides in 1/148, and *Bartonella* peptides 4/148. (**C**) OspC was the most represented protein in the peptide repertoire derived from acute Lyme borreliosis and non-acute tick-borne disease patients. While 109 peptides were identified once, multiple peptides were identified for Outer surface protein C, Variable large protein, Transcription elongation factor GreA, UTP—glucose-1-phosphate uridylytransferase (15, 4, 4, 3, and 3 peptide hits, respectively).

In the validation phase of the study, which was conducted blinded to the patient clinical category, urine samples from 148 non acute patients (n = 36 PTLDS; n = 112 clinically suspected of tick-borne illnesses) and 150 new healthy and diseased controls were analyzed. Patients (M/F = 0.45, median age = 48, IQR = 28.1) reported with symptoms including fatigue, musculoskeletal pain, cognitive impairment and a history of tick bite, fever, and/or EM rash. The control set (M/F = 1.75, median age = 35, IQR = 22) included healthy controls and disease control patients with clinical history of Chagas disease, tuberculosis and traumatic brain injury. 279 unique peptides specifically attributed to microorganisms belonging to the genus *Borrelia, Babesia, Anaplasma, Ehrlichia, Bartonella, Rickettsia,* and known to be pathogenic in humans were identified in n = 108/148 patient samples. No peptides from TBEV and Powassan virus were identified in patients or controls. Peptides matching *Borrelia *sp. (n = 160, Fig. 3A), *Babesia *sp. (n = 62, Fig. 3B), *Rickettsia *sp. (n = 8), *Francisella *sp. (n = 6), *Anaplasma *sp. (n = 8), *Bartonella *sp. (n = 15), *Ehrlichia* (n = 12) (Tables S4, S5) were identified. In 89/148 patients at least 1 peptide from *Borrelia* was identified. Within this subset, 29% (26/89) carried peptides from *Babesia* proteins, 8% (7/89) for *Rickettsia*, 7% (6/89) for *Anaplasma*, 10% (9/89) for *Bartonella*, 6% (5/89) for *Ehrlichia* (Tables S4, S5). The case group included 8 pediatric patients. Peptides matching *Borrelia* were detected in 4/8 pediatric patients, while peptides from *Babesia* were found in 3/8 patients and one peptide from *Rickettsia* and 2 from Ehrlichia were found in 1/8 patients respectively (Table S6). In the validation set, a single peptide from tick-borne organisms was found in 21/150 non-TBI controls (15%). No control presented more than 1 peptide. As defined in the training phase of the study, a patient was considered positive for a given organism if at least two unique peptides deriving unambiguously from such organism were identified. According to this criterion, 59/148 non acute patients (40%) were positive for at least one tick-borne pathogen and all the controls were negative. n = 48 were positive for *Borrelia*, n = 17 positive for *Babesia*, n = 4 were positive for *Bartonella*, n = 2 were positive for Ehrlichia, n = 8 were positive for *Borrelia* and *Babesia,* n = 1 was positive for *Borrelia* and *Bartonella*, n = 1 was positive for *Babesia* and *Bartonella*, n = 1 was positive for *Babesia*, *Bartonella* and *Anaplasma*. Therefore, 48/148 patients (32%, Fig. 4A,B) were positive for only one pathogen and 10/148 samples (7%) were positive for 2 pathogens, and 1/148 (0.7%) was positive for 3 pathogens suggesting the co-existence of multiple infections (Fig. 4A, Fig. S5). Seven unique peptides belonging to the genus *Francisella* and common to the species *tularensis, novicida*, and *hispaniensis*, *persica* were also identified. Urinary peptides from species known to be non-pathogenic suggest a commensal host-microbe interaction (Table S7).

### Urinary pathogen peptides revert to undetectable levels after symptom resolution

Longitudinal study of three patients provided anectdotal evidence that urinary peptides revert to undetectable levels after symptom resolution. Pre and post treatment urine collection was obtained from two acute LB patients. In one case (patient # 108838), three *Borrelia* peptides were identified at the time of positive serology and EM rash. Complete clearing of *Borrelia* peptides was observed after symptoms resolution with two 14-day courses of doxycycline. In the second patient (# 453742), urine was collected at different time points: (1) after tick bite and before EM rash, (2) after development of the characteristic EM rash and before antibiotic treatment, (3) after 2 days of doxycycline treatment while the patient was still symptomatic. Three *Borrelia* peptides were detected in the urine before development of the EM rash. *Borrelia* peptides were then confirmed in the urine of the untreated, symptomatic patient. *Borrelia* peptides were also detected after two days of doxycycline treatment while the patient was still symptomatic (Table 3). A decline in peptide numbers following treatment was found for patient # 957477, positive for Erhlichia chaffeensis, whose urine was collected the first day of treatment as well as after two and four weeks (Table 3).

**Table 3:** Longitudinal study of two Lyme borreliosis patients, and one non-acute tick-borne disease patient (NA-TBI).

Sample ID	Clinical status	Pathogen	Before EM (after tick bite)	Before treatment—presence of EM	During treatment	Resolution of symptoms
108838	Acute Lyme borreliosis	Borrelia	N/A	3	N/A	0
453742	Acute Lyme borreliosis	Borrelia	3	2	2	N/A
957477	NA-TBI	Ehrlichia	N/A	2^a^	1	0

In patient 108838 (acute LB), Borrelia-specific peptides are identified in presence of acute symptoms (EM rash) and no peptide is detected after symptom resolution (4 weeks of doxycycline). In patient 453742 (acute LB), Borrelia-specific peptides were identified after tick bite but before development of an EM rash. Peptides were detectable in the pre-treatment stage and in presence of EM rash. Peptide count decreased during early treatment (2 days of doxycycline) when the patient was still symptomatic. In patient 957477 (non-acute tick-borne disease) 2 Ehrlichia peptides were identified in the presence of symptoms before starting treatment, 1 peptide after 14 days of doxycycline and no peptides after 4 weeks of doxycycline.

^a^EM rash was not present.

### *Borrelia*-specific urinary peptides are associated with chemotaxis, transmembrane transport, immune evasion and metabolism

Peptides (N = 160) from *Borrelia* species were the most abundant among the tick-borne infection pathogens investigated. Gene Ontology (GO) analysis of biological functions indicated that a large number of proteins were associated with chemotaxis, biosynthesis, transmembrane transport, immune evasion and DNA metabolism (Table S8). Chemotaxis and motility are required for *Borrelia* to establish infection in the mammalian host^21^. In this study, we identified peptides specific for chemotaxis and motility associated proteins including flagellin, CheA, and MotA. Transmembrane transport plays a role in drug resistance, in parasite-host interaction, in cell signaling and virulence^22^. Urinary peptides associated with transmembrane transport proteins included ABC transporter permease, acriflavine resistance protein, and mechanosensitive ion channel. In response to mammalian host immunity, *Borrelia* modulates its transcriptional activity to facilitate dissemination and immune evasion (Table S8)^23^. Cell envelope proteins are involved in a number of processes required for *Borrelia* to establish infection in the mammalian host, including cell adhesion, cell invasion and immune escape^22^. Examples of proteins in this category include outer surface protein A (OspA), outer surface protein B, and outer surface protein C (OspC) (Fig. 3A, Tables S8, S9). Among the proteins identified in the urine of non-acute patients there were 6 known seroreactive proteins: OspA, OspB, OspC, Flagellin, Porin, P37 and OppaIV. OspC and Flagellin are also included in the two-tiered Lyme borreliosis serology according to CDC criteria^24^. 55 identified proteins are known to be localized in the membrane region (of which n = 10 are known to be localized in the outer membrane and n = 4 in the inner membrane), 54 in the cytoplasm and 12 in the flagellum (Fig. 3A, Fig. S6, Tables S8, S9). The most represented *Borrelia* proteins in the peptide repertoire were OspC (17 peptide hits), variable large protein (4 peptide hits), and transcription elongation factor (4 peptide hits) (Fig. 4C). More than 65% of the *Borrelia* derived urinary peptides identified in this study (109/160) were detected only once (Fig. 4C).

### *Borrelia* peptides in the cerebrospinal fluid of a clinically suspected neuroborreliosis patient are also detectable in the urine (anectdotal)

The experimental protocol described in Fig. 2 and the algorithm of Fig. S1 were applied to matched cerebrospinal fluid (CSF) and urine samples from a clinically suspected Neuroborreliosis patient. Ten months prior to sample collection, the patient experienced worsening of neurological symptoms, including fainting, ataxia, and tremors in the face, neck and hands. Two peptides were detected in the CSF: OspC peptide LKEKHQDLGVANGDTTDNNAK and Acriflavine resistance protein peptide VTSNLDVEK. The same OspC peptide was detected in the urine.

### The number of urinary peptides correlates with presence or absence of symptoms in non acute tick borne disease patients

Symptoms reported by non acute patients (PTLDS and patients with clinical suspicion of tick-borne illnesses) included previous EM rash, joint pain, fatigue, fever, facial palsy, and other neurological symptoms. A score of a 0 and 1 was attributed in absence or presence of any symptom designated in Table 1. Using an ordinal regression model, we found that for those subjects where clinical data were available (N = 46), urinary peptide number was positively correlated with presence or absence of symptoms (ordinal regression pseudo-R^2^ = 0.392, p-value = 0.010) (Table S10).

### Alignment analysis informs verification of protein database annotation and unambiguous species attribution of urinary peptides

Alignment analysis within evolutionarily related organisms in the clade was conducted to achieve two goals: (1) verification of the protein database annotation, and (2) attribution of the peptide to an organism at the species level. In order to achieve the former, full length sequence of the protein associated with each urinary peptide was retrieved from the highest-ranking species in FASTA format and compared to homologous proteins (data from Basic Local Alignment Search Tool (BLAST), run on databases downloaded on February 15th 2020). In the case of Borrelia, annotated species of *Borrelia* were used, including: *Borrelia burgdorferi*, *Borrelia garinii*, *Borrelia afzelii*, *Borrelia bissettii*, *Borrelia bavariensis*, *Borrelia mayonii*, *Borrelia miyamotoi*, *Borrelia hermsii*, *Borrelia turicatae*, *Borrelia chilensis*, *Borrelia duttonii* (Fig. S7). If the protein demonstrated greater than 60% homology, over the full query length, with other species in the query, then the database annotation was validated. In this study, protein database annotation was validated in all the instances and no rejection was necessary. In order to attribute peptides to an organism at the species level, the peptide sequence was studied in the context of homologous proteins in the clade. A peptide was unambiguously attributed to a species if the peptide sequence had 100% match with the given species and less than 90% sequence identity to any other species investigated (Fig. S7). Species variation can be an important cause of diagnostic inaccuracy due to lack of reactivity of detection reagents^22^.

### Orthogonal technologies western blot analysis and parallel reaction monitoring confirm urinary peptide identification

**Figure 5:**
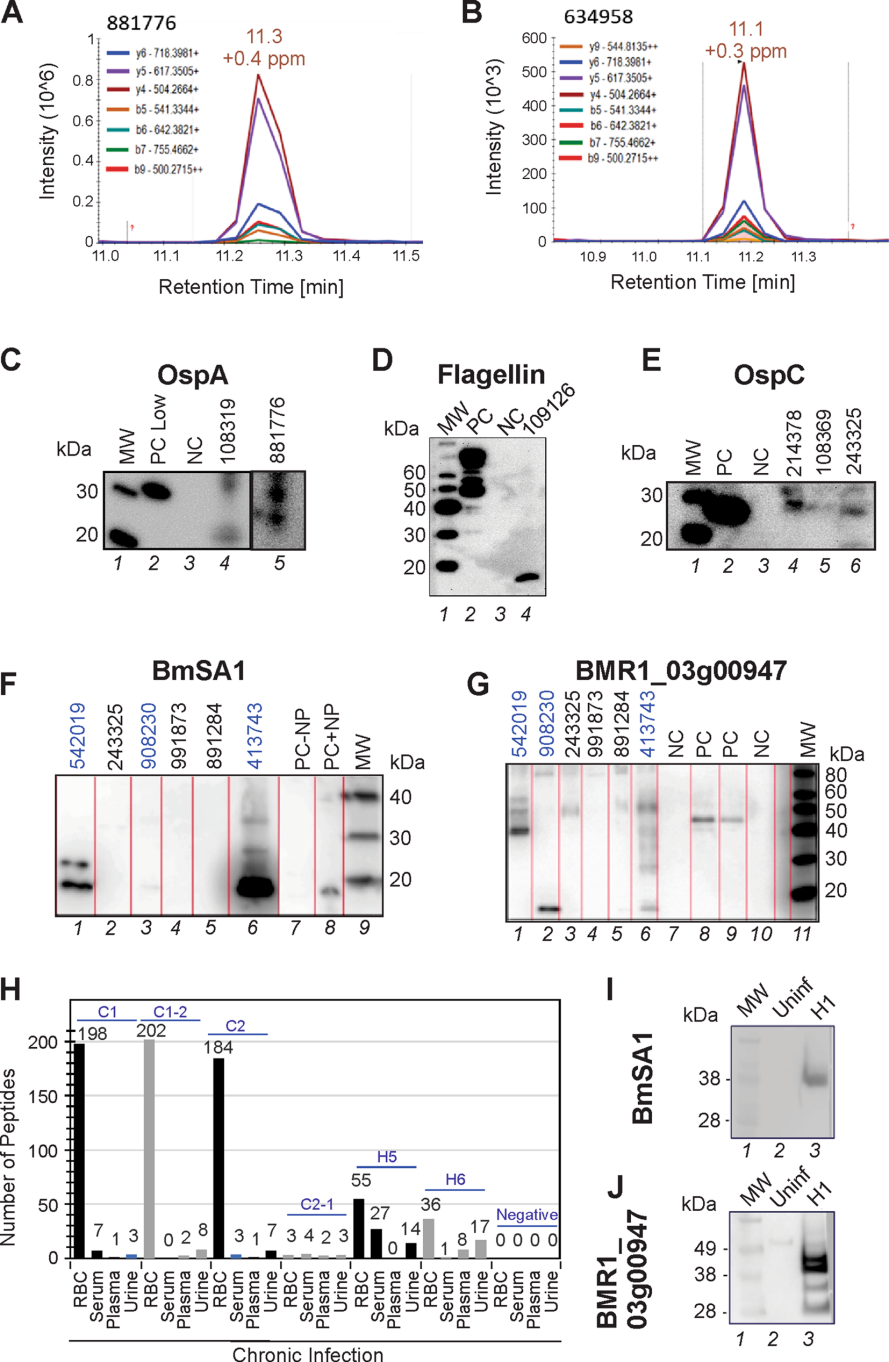
MS/MS findings verification. (**A**,**B**) Parallel reaction monitoring (PRM) verification of a peptide deriving from Outer Surface Protein A (OspA). OspA peptide AVEIKTLDELK was found in the discovery phase and confirmed by PRM in 2 non acute patient samples. (**C**–**E**) The presence of OspC (samples 214378, 108369, 243325), OspA (samples 108319, 881776) and Flagellin (sample 109126) was confirmed in by means of western blot analysis (*MW *molecular weight, *PC *positive control, *NC *negative control). Positive bands were found at 31 kDa, 27 kDa, and 18 kDa for OspA, OspC and Flagellin respectively. Since the positive control used in the western blot for flagellin is a flagellin–maltose-binding protein fusion protein, multiple bands above 40 kDa were detected (right image, PC lane). Patient 109126 yielded a band reactive to the anti-flagellin antibody at a lower molecular weight than the expected band at 34 kDa for the full length protein. This might be due to a truncated form of the protein. Uncropped original images are reported in Figure S16. (**F**,**G**) Western blot analysis detected the presence of Babesia BmSA1 and BMR1_03g00947 antigens in patients 542019, 413743, and 908230 who had at least one Babesia peptide in their urinary peptidome analysis conducted with mass spectrometry (2, 3, and 1, respectively). Patients 891284, 991873, and 243325 who had 2, 1, and 1 Babesia mass spectrometry peptide, respectively, did not show clear reactivity towards these two antibodies. Bands at a molecular weigth lower than the full length protein (48.7 kDa and 35.4 kDa for BMR1_03g00947 and BmSA1, respectively) are indicative of degradation products that pass glomerular filtration. PC is *Babesia* infected hamster red blood cells (RBC) diluted in urine. NC is healthy donor urine. PC − NP and PC + NP is positive control in absence and presence of nanoparticle processing, respectively. (**H**) *Babesia* peptides were identified in the blood and urine of a hamster animal model at early^36^ and late stages of disease with high sensitivity. (**I**,**J**) Verification of MS analysis results of two surface/secreted *B. microti* antigens, BmSA1 and BMR1_03g00947, by Western blotting of hamster RBC lysates.

Pathogen-specific, urinary peptides were confirmed using affinity particle enrichment and two orthogonal approaches—Parallel Reaction Monitoring (PRM) and western blotting. For the former, N = 3 peptides from OspA, OspC, and flagellar proteins were chosen based on the discovery full scan MS/MS results on patient samples (Figs. S8–S14) and on additional LC–MS/MS analysis of healthy volunteer urine spiked with recombinant proteins. Peptide AVEIKTLDELK deriving from OspA was confirmed by PRM analysis in four patient samples that yielded this peptide in the discovery MS/MS analysis (Fig. 5A,B, Fig. S15). In these samples, peptide LKNSHAELGVAGNGATTDENAQK from OspC and NDVSEEKPEIK from flagellar motor switch protein were not detected. To further verify MS/MS results, additional sample aliquots from six previously tested patients were processed with affinity particles and tested by western blot analysis using previously validated antibodies for OspA, OspC and Flagellin. Western blot results were concordant with MS/MS findings for OspC (2/3 patients), OspA (2/2 patients), and Flagellin (1/1 patient) (Fig. 5C–E). Western blot analysis detected the presence of Babesia BmSA1 and BMR1_03g00947 antigens in patients 542019, 413743, and 908230 who had at least one Babesia peptide in their urinary peptidome analysis conducted with mass spectrometry (2, 3, and 1, respectively). Patients 891284, 991873, and 243325 who had 2, 1, and 1 Babesia mass spectrometry peptide, respectively, did not clear show reactivity towards these two antibodies. Bands at a molecular weigth lower than the full length protein (48.7 kDa and 35.4 kDa for BMR1_03g00947 and BmSA1, respectively) is indicative of degradation products that pass glomerular filtration (Fig. 5F,G).

### *Babesia microti* derived peptides are detected in the urine and blood of an animal model of persistent infection and correlate with parasitemia

In order to substantiate the hypothesis that peptides derived from a tick-borne pathogen in later phases of infection can be detected in peripheral body fluids such as urine, we analyzed bodily fluids derived from golden hamsters (*Mesocricetus auratus*) infected with *Babesia microti.* Six golden hamsters with parasitemia ranging from 0 to 42% and one uninfected control were studied at different times after infection, ranging from 3 to 6 months to mimic chronic infection. PCR to *Babesia* ITS regions^25^ was used to confirm infection loads (Supplementary Methods and Supplementary Results). 870 unique *Babesia* peptides belonging to 319 proteins were identified in red blood cells (RBC), plasma and urine. The number of *Babesia* proteins in the RBC compartment correlated with levels of parasitemia by linear regression analysis (p < 0.0001, Table S11). Even though the site of *Babesia* infection is the blood, *Babesia* derived peptides were detected in the urine of hamsters at late stage of infection (Fig. 5H, and Table S12), where parasitemia was low or undetectable (H5 and H6 yielded 14 and 17 urinary peptides and parasitemia of 0% and 1%, respectively, Fig. 5H, and Table S11). Among the hundreds of peptides identified (Supplementary Table S12), members of the BMN2 family were detected in RBCs (BMR1_03g00020, BMR1_02g04265; BmR1_04g09980) and in urine of chronic hamsters (BMR1_02g04265). BMN2 family members are considered to be relevant in the immune evasion as suggested by their high mutation frequency and low immunogenicity^25^, and were previously reported to be highly expressed antigens in *Babesia microti.* 3/62 (5%) *Babesia* peptides found in urine of non acute patients (Tables S4 and S5) were also identified in body fluids of infected hamsters (Table S12, Magni et al.^26^). These include importin-5 (BMR1_02g00750), guanine nucleotide-binding protein subunit beta-2-like 1 protein (BmR1_04g08285), hypothetical protein (BMR1_03g04255).

## Discussion

The goal of this study is to introduce a method for investigating candidate pathogen specific peptides in patients diagnosed with acute Borreliosis or suspected of tick-borne illnesses including Borreliosis, Babesiosis Anaplasmosis, Ehrlichiosis, Tick-borne encephalitis virus, Powassan Virus disease, Rickettsiosis, TBRF, and Tularemia^27^. Past studies have focused on serology, culture, or erythrocyte microscopy (Babesiosis) to study single tick-borne infections, and little is known the incidence of co-infections^7,10,12,28^.

The analytical sensitivity of MS analysis is currently in the range of 10–100 ng/mL when analyzing complex matrices without pre-analytical processing^11,29^, hence mass spectrometry analysis applied directly to body fluid samples lacks the sensitivity needed for low abundance pathogen derived protein detection. We show that pre-processing the sample with affinity hydrogel particles^18,19,30–34^ concentrates the low abundance biomarkers to achieve mass spectrometry sensitivity in the low picogram/mL range^32^. Additionally, the present method ensures linearity and precision of the assay in physiologically relevant protein concentration ranges (Table S1). Affinity hydrogel particles^18,19,30–34^ consist of polymeric networks functionalized with high affinity chemical baits that capture, concentrate, and preserve solution phase analytes in one step, while excluding interfering high abundance proteins (Supplementary Methods) that would otherwise negatively affect the analytical sensitivity of mass spectrometry analysis^35^. In previous studies we successfully pre-processed biofluids with hydrogel particles dramatically increase the sensitivity of the analytical technique used downstream and allowing the detection of previously undetectable markers of *Mycobacterium tuberculosis*^18^, *Toxoplasma gondii*^20^, and *Trypanosoma cruzi*^19^, and *Babesia microti*^26,36^. We also combined hydrogel particle pre-processing with a highly sensitive immunoassay^30^ to detect OspA, a relevant biomarker for Lyme borreliosis.

Our 3-tier authentication algorithm, which requires 100% amino acid sequence identity with tick-borne pathogen proteins, evolutionary taxonomic verification for related pathogens, and lack of identity with human or any other organism (Fig. S1), dramatically reduces the number of false positives that would have been otherwise called using direct MS sequencing by conventional MS software (Fig. S4). Biologic and technical validation of the algorithm employs CDC criteria, serology positive acute Lyme patients, concomitant urine western blot immunoassays, orthogonal targeted identification using PRM, and an animal model of persistent Babesiosis. PRM is a targeted proteomic approach able to simultaneously monitor all fragment ions derived from selected peptides with high resolution and accuracy^37^. Orthogonality between discovery phase and PRM can be obtained through a different combination of fragmentation strategies and mass analyzers. In discovery, precursors were fragmented with collision induced dissociation (CID), and product ions measured in the ion trap analyzer; in PRM, fragmentation was obtained with high energy collision induced dissociation (HCD) and product ions were measured in the Orbitrap mass spectrometer. Stringent mass tolerance filters (≤ 1 ppm) were applied to the product ions in the spectra, allowing for a highly confident peptide identification.

The method yielded zero false positives in 250 diseased and healthy controls and identified up to five specific urinary *Borrelia* peptides in 10 acute LB patients, including proteins that are part of the panel for the standard Lyme serological test (Table 2). For 2 of the acute LB patients as well as for a PTLDS patient we were able to obtain urine samples at different time points (Table 3). As a result, we were able to anectdotically observe a decrease in the number of peptides during antibiotic treatment and absence of tick-borne pathogen peptides after successful treatment and symptom remission.

Addressing the question of persistent infection, 279 different urinary peptides, derived from the surface or subcellular compartments of pathogenic strains of tick-borne pathogens, were identified in non acute patients (PTLDS and patients with clinical suspicion of tick-borne illnesses). In 40% (n = 59/148) of them we identified two or more peptides unique for at least one tick-borne pathogen and the number of urinary pathogen derived peptides correlated with the presence or absence of symptoms (p < 0.011) reported by the treating physician when available. 32% (n = 48/155) of patients presented peptides derived from one pathogen, while 7% presented (n = 10/148) peptides from two pathogens, and less than 1% (n = 1/148) presented 3 pathogens.

*Borrelia* was the most frequently represented organism. A large number of identified proteins are located on the membrane surface and several are known to be antigenic^38–41^. It is important to note that *Borrelia* undergoes several changes during host infections which require the production of new membrane proteins that could be used for immune evasion or adaptation to the new environment^38,42,43^. Multiple proteins identified herein are currently recognized as antigens in the standard serological test: OspC, Flagellin^44^. Among the *Borrelia* genus, the highest number of peptides were derived from species related to Lyme borreliosis.

In this study, at least two different peptides associated with *Borrelia* were found in n = 48/148 non acute patients suspected of tick-borne illnesses. Represented *Borrelia* species included Lyme-associated as well as TBRF-associated species. In many subjects both Lyme and TBRF associated species were found (Tables S4 and S5). We identified 42 unique peptides specific for TBRF *Borrelia* species (Tables S4, S5), and 24 peptides from *Borrelia miyamotoi*, which is being diagnosed in the United States in an increasing number of patients^45^. Recent evidence shows that TBRF *Borrelia* species can also be carried by Ixodes ticks, the same vector that transmits Lyme borreliosis^46–48^. TBRF is an often-neglected disease and may go underdiagnosed in many patients. In fact, TBRF patients can yield a positive serology for Lyme borreliosis because of proteins with overlapping antigenic similarities with Lyme *Borrelia* species^49,50^, thus TBRF true prevalence can be underestimated.

We chose to build the mass spectrometry proteomics database to comprise as many pathogenic species as possible, including pathogen species that are currently thought to be limited to narrow geographic location (e.g. *Borrelia mayonii* detected only in the US upper Midwest, Wisconsin and Minnesota). The reason of this choice was two fold: (1) a larger database increases the algorithm sensitivity and specificity; (2) we wanted to devise an analytical tool that is robust across all geographical locations, in face of the possibility that climate change may induce unexpected relocations of ticks and vertebrate reservoirs.

**Figure 6:**
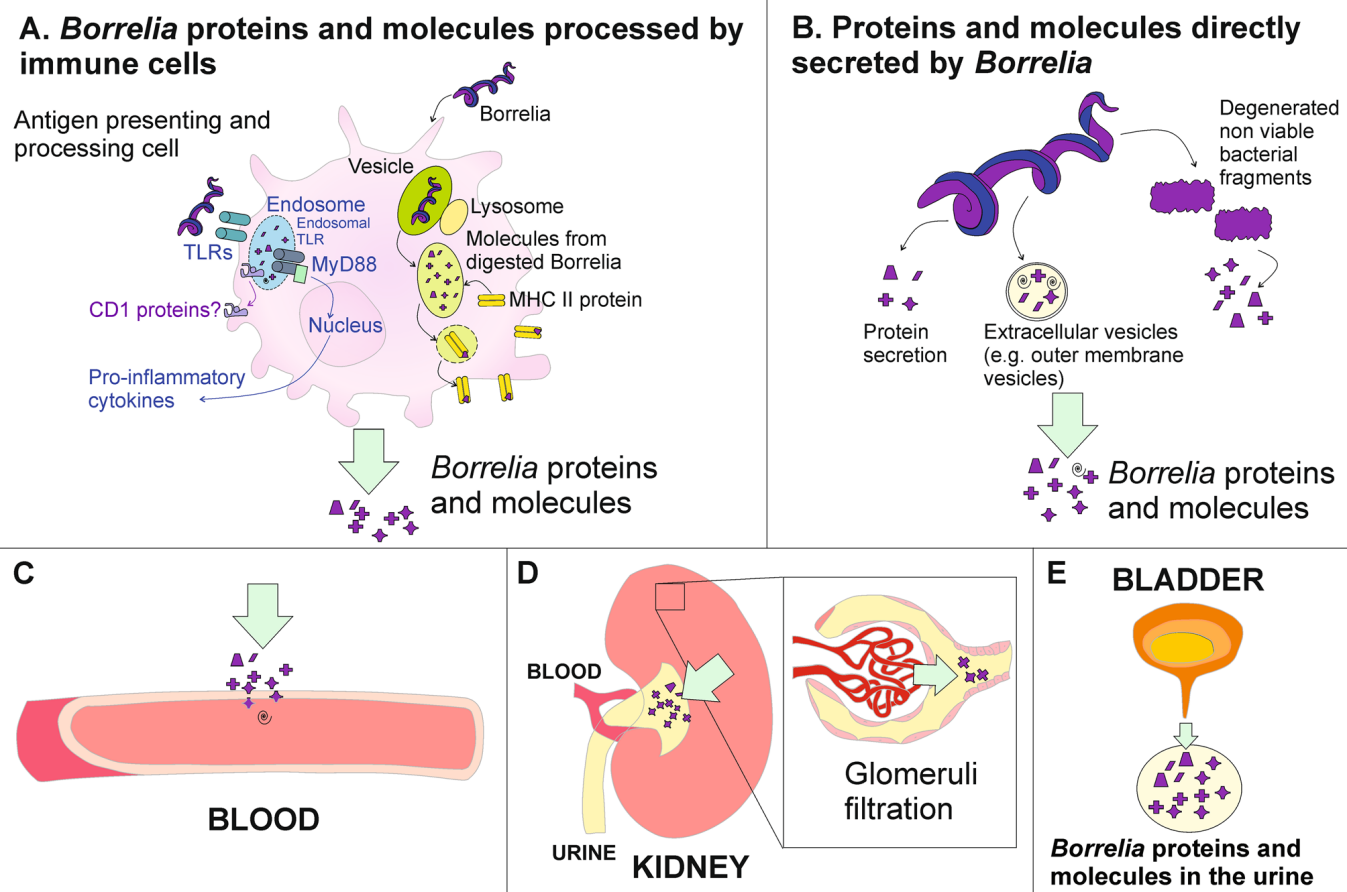
Proposed sources and shedding mechanism of *Borrelia* derived proteins and molecules that are detected in the urine. (**A**) During the immune response, *Borrelia* proteins and molecules are processed and presented by antigen presenting cells (e.g. dendritic cells and macrophages) in conjunction with MHC II or CD1 proteins. Digested Borrelia antigens bind to endosomal TLRs and trigger the production of pro-inflammatory cytokines. Bacterial peptides are released following apoptosis of APC, or by secretion of soluble molecules or extracellular vesicles. (**B**) Proteins and molecules can be directly secreted by *Borrelia* into the extracellular environment, released in extracellular vesicles, or liberated by degenerated non-viable bacterial fragments. (**C**) Proteins, protein fragments and molecules of a size inferior to 60 kDa can permeate blood vessel walls and enter the blood circulation, (**D**) be filtered in the kidneys through glomeruli filtration and (**E**) accumulate over time in the urine.

We hypothesize that *Borrelia* peptides present in the urine can be derived from two distinct sources: (a) pathogen peptides previously processed by immune cells, such as antigen presenting cells, and (b) peptides that are released directly by viable or non viable spirochetes (Fig. 6). Bacterial cell wall fragments have been previously detected in rats months after inoculation^51^ and a recent study shows the persistence of Borrelia peptidoglycan in the synovial environment for several weeks after antibiotic treatment^17^. It is also known that antigens from several pathogens, including *Borrelia*, can persist in the lymph nodes in association with antigen presenting cells such as dendritic cells (DC)^52^ and also with non-hematopoietic cells such as lymph node stromal cells^53,54^ for at least several weeks after infection resolution^53^. DCs play a crucial role in the development of a specific T-cell mediated response to *Borrelia* after phagocytosis^55,56^. Published studies support the evidence that *Borrelia* peptides are displayed in conjunction with MHC class II molecules^52^ and with CD1 proteins^57–59^ on the surface of professional antigen presenting cells. Additionally, endosomal Toll Like Receptors bind to *Borrelia* processed antigens and initiate a MyD88 mediated signaling response that leads to expression of IL-1beta and inflammatory cytokines^60^. As a result of their persistence in the lymph nodes, peptides can be shed over time due to apoptosis of the antigen presenting cell or to secretion of soluble molecules or extracellular vesicles^61^. While the concentration of these peptides is unknown, we cannot exclude that it may still fall within the analytical range of detection. In support of this hypothesis, we have found that the OspC peptide AILKTNGDKTLGAAELEK, frequently recurring in this study, is compatible with MHC class II binding using the algorithm RankPep^62,63^. This computational method predicts the likelihood of peptide–MHC class II binding based on primary and secondary structure parameters. Predictions were conducted using HLA-DR haplotypes, based on the published evidence that HLA-DR frequency is significantly increased in patients that suffer from antibiotic refractory Lyme rheumatoid arthritis^64^ and Neuroborreliosis^65^. Alternatively, *Borrelia* peptides can derive directly from spirochetes present in different tissues (e.g. synovial connective tissue^66^, brain^67^, and heart^67^). *Borrelia* is known to secrete soluble proteins^68^ and to produce extracellular vesicles^76^ that contain a variety of molecules such as nucleic acids, intracellular and membrane proteins, lipopolysaccharides, phospholipids, and metabolites. Additionally, peptide molecules can be released by degenerated, non viable bacterial fragments (Fig. 6B). Peptides can then reach the blood circulation by permeating the blood vessel wall to enter the blood circulation. They are then filtered in the kidneys by the glomeruli and accumulate over time in the urine (Fig. 6C–E)^69^. This momentary shedding of pathogen molecules from living or non viable organisms in the blood is integrated over time as they accumulate in the urine.

The number of pathogen peptides in the urine of untreated individuals were not higher than in PTLDS and NA-TBI patients. The scientific premise is that the number of urinary pathogen peptides correlates with pathogen burden, as demonstrated in other infections including tuberculosis^18^, Babesiosis^26,36^ and Chagas disease^19^. In the acute, untreated phase, the total body burden of *Borrelia* organisms is low and there may have not been sufficient time for immune mediated attack and break down of pathogen organisms into antigenic peptides that are small enough to pass through glomeruli filtration (Fig. 6). We expect that, due to low body burden of disease in the early stages and late stages of *Borrelia* infection, we would find similar number of peptides.

This study has several limitations. Only 10 acute samples were analyzed in the training phase of the algorithm. Due to the fact that we have high precision and large effect size (3.35), even using a very low number of observations in the Lyme group, we achieved a power of 0.9999 (alpha = 0.05, Wilcoxon Mann Whitney).

We lack any independent means (e.g. culture or PCR) to verify a tick-borne pathogen infection in non acute patients with pathogen peptides in the urine. Moreover, while symptomatic non-TBI controls used in this study were collected from individuals living in geographical areas wth very low incidence of Borreliosis and healthy non-TBI controls had no history of a clinical diagnosis of tick borne illnesses, participants were not tested for Lyme and other TBI serology. Under standard of care, it is not justified to conduct a serology test on a patient in absence of clinical suspicion of Lyme disease or tick borne illness.

A binary scale was used to assess presence or absence of symptoms. This choice was motivated by the fact that the location of treating physicians contributing to this study ranges from Europe to the USA east coast, the Baltimore Washington area, and the USA west coast. Thus we judged that there is no practical way to normalize a quantitative symptom level severity score across the physicians. Nevertheless, as has been previously shown a yes/no binary recording of a subjective symptom, such as level of pain, can be just as accurate across physicians as a ranking score^70,71^ Although a binary approach provides less information and granularity than a ranking score, in the interest of increasing accuracy and rigor we chose to use presence or absence of symptoms that fall under the IDSA guidelines^13^. We report the correlation as merely descriptive and we cannot consider it clinically for a treatment decision. It would be interesting to investigate the findings further on a larger dataset, and possibly grade the severity of each symptom, after using some means to normalize the scoring across treating physicians.

We did not use a *Borrelia* animal model for this study; nevertheless, we attempted to study the concept of chronic disease shedding in the well-established *Babesia* animal model. In previous papers^26,36^ we showed the ability to identify Babesia peptides in urine of acutely infected hamsters; in the current paper we are showing the persistence of peptides in chronically infected hamsters even in presence of very low or undetectable parasitemia.

A further limitation of the study relates to the unknown temporal variation in shedding of tick-borne pathogen proteins in a patient who harbors an active infection. Thus, if a symptomatic patient is negative for urinary tick-borne pathogen specific peptides, even though they are actively infected, they might not be shedding the pathogen protein at the time the urine was collected. On the other hand, pathogen specific peptides shed in the urine may be leftover from a prior active infection and may reflect discharge of intracellular processed proteins following immune cell phagocytosis (Fig. 6). These limitations may be addressed in future studies that sample urine longitudinally over time before and after antibiotic therapy in patients with acute Lyme borreliosis who successfully clear the symptoms and in those who do not.

Despite these limitations, the method is cost-effective, is not restricted to specialized centers, and has potential for routine diagnosis of TBI following thorough independent validation.

## Methods

### Study design

A method consisting of sample pre-analytical concentration, mass spectrometry analysis and a novel peptide authentication algorithm was applied to 408 urine patient specimens (Table 1) in order to discern the presence of peptides belonging to the proteome of selected tick-borne pathogens. Urine samples were subjected to pre-analytical concentration and mass spectrometry analysis. Urine specimens were divided in a training (N = 110 patients, 10 cases and 100 controls) and a non-overlapping validation (N = 298 patients, 148 cases and 150 controls) set that were used to establish the parameters of the peptide authentication algorithm to ensure that identified peptide sequences were uniquely attributable to tick-borne pathogens. Validation was conducted blinded to clinical category. The algorithm included three steps: (1) determination of physical and statistical parameters for mass spectrum matching, (2) BLAST searches of peptides longer 7 amino acids to ensure that the selected peptide sequence has percentage identity lower than 100% with proteins of non-tick pathogen organisms, and (3) validation of protein database annotation via alignment with homologous proteins of evolutionary related organisms in the clade (Fig. S1). At the conclusion of the analysis, we performed manual quality check of spectra and we did not find any discrepancy or incorrect attributions. Peptides identified with the method were verified using Western Blot analysis^30^, parallel reaction monitoring^20^ and an animal model of persistent *Babesia microti* infection^26^. The correlation of urinary peptides derived from tick-borne pathogen with patient symptoms was investigated.

### Patient study cohorts

Urine samples were collected from consented (IRB Pro00008518, Chesapeake IRB) patients who were suspected of having tick-borne diseases in different geographic regions at high risk for tick-borne diseases: US East Coast, including New England, Northern Virginia, Maryland and Washington, DC (N = 122), US West Coast (N = 7), and Northern Europe (N = 29) (clinics: Hope McIntyre, MD, Maryland; Deborah Hoadley MD LLC, Massachusetts; Christine Green, MD, California, Innatoss Laboratories B.V., Netherlands). Acceptance criteria for (1) acute LB patients (N = 10) included the characteristic *erythema migrans* (EM) rash and positive two-tier LB serology according to CDC criteria The urine was collected before treatment regimen for all patients. All 10 acute patients were verified to be two tier serology positive 3 to 6 weeks later. (2) PTLDS patients were identified according to the Infectious Disease Society of America guidelines^13^. Patients were considered PTLDS if (a) they were previously diagnosed for Lyme disease (based on EM rash and positive two-tier serology) more than 6 months before the urine sample was collected, (b) if Lyme Borreliosis symptoms ameliorated following antibiotic treatment, and (c) if patients presented with fatigue, musculoskeletal pain, or cognitive impairment according to the IDSA criteria at the time of urine collection. Symptoms were judged by the physician to be functionally disabling. (3) Other non acute patients suspected of tick-borne disease (n = 112): patients had a history of tick bite and a history of clinical diagnosis of a tick borne illness. For patients suspected of a prior diagnosis of Lyme Borreliosis, clinical information, or IDSA categorized symptoms were not sufficient to fit the requirement of PTLDS according to the standards specified above. The ticks that bit the patients were not formally identified. This study met the requirements for IRB approval (Pro00008518, Chesapeake IRB) and followed principles of the Declaration of Helsinki. All participants provided written informed consent prior to the study. For minors, written informed consent from a parent or legal guardian was obtained. All methods were performed in accordance with relevant guidelines and regulations. Clinical and demographic data included age, sex, previous tick-borne disease diagnosis, self-reported symptoms, and physician assessed symptoms. Urine specimens were collected from 215 diseased controls and 35 healthy volunteers (Table S3) from the US and Peru. Diseased controls included hospitalized patient affected by HIV infection, tuberculosis, Chagas disease, and acute respiratory distress syndrome (ARDS) following traumatic brain injury. Diagnosis of pulmonary tuberculosis was verified by sputum smear microscopy and culture methods. HIV infection was confirmed by HIV nucleic acid amplification test and CD4+T cell count. Chagas disease status was determined by microscopy examination of blood smears and quantitative PCR analysis of blood. ARDS patients were diagnosed using the *Berlin Definition* criteria^72^ that include bilateral lung infiltrates detected with chest X-rays, pulmonary capillary pressure ≤ 18 mmHg, and oxygenation levels PaO_2_/FiO_2_ ≤ 200 mmHg. Healthy volunteers were asymptomatic and had no history of tick-borne infection. Both diseased controls and asymptomatic healthy controls were not tested for Lyme serology. Under standard of care, it is not justified to conduct a serology test on a patient in absence of clinical suspicion of Lyme disease or tick borne illness.

### Collection of bio-fluids from patients under evaluation for acute Lyme borreliosis and non acute patients suspected of tick-borne illnesses

Matched coded clinical records and LB serology results were also provided under patient consent. Urine samples from US-based collection sites were refrigerated immediately after collection and sent to George Mason University in refrigerated containers within 24 h from collection; samples were then frozen at − 80 °C upon arrival. Urine samples from European collection sites were immediately frozen upon collection, transferred to George Mason University in dry ice and stored at − 80 °C. Cerebrospinal fluid (CSF) was collected by lumbar puncture. The CSF sample was immediately placed in dry ice, shipped from Massachusetts to Mason in dry ice, and kept at − 80 °C until analysis.

### Affinity particle processing of biofluids from patient subjects

500 μl of cerebrospinal fluid from patients suspected of tick borne illnesses were centrifuged at 3750×*g* for 15 min, the pellet was discarded, and supernatant was recovered and diluted with 500 µl Tris–HCl 50 mM, pH 7.2. Urine samples (at least 42 ml) were thawed in warm water (approx. 37 °C) on an orbital shaker. Urinalysis was performed using a Multistix 10 SG reagent strip. Urine was transferred into 50 ml tubes and centrifuged at 3700×*g* for 15 min. Urine was decanted into a new tube and the pellet was discarded. pH was adjusted to 5.5 incrementally adding 1 M hydrochloric acid or 1 M sodium hydroxide. 40 ml of urine sample was transferred into a 50 ml polycarbonate tube. Urine and CSF samples were incubated with 200 µl affinity particles (10 mg/ml) for 30 min at RT. CSF samples were centrifuged at 16,100×*g* for 20 min while urine samples at 19,000×*g* (Beckman Avanti JXN-26 Centrifuge) for 45 min. Supernatants from CSF and urine samples were discarded. Particle pellet was washed twice by vigorously resuspending it in 1 ml 18 MΩ-cm water followed by centrifugation at 16,100×*g* for 20 min. Supernatant was discarded and particle pellet was resuspended in 20 µl of elution buffer solution (4% sodium dodecyl sulfate (SDS) in 50 mM ammonium bicarbonate), and incubated for 20 min at RT. Samples were centrifuged at 16,100×*g* for 20 min. Eluates were saved and transferred into new tubes and processed for mass spectrometry as described further.

### Mass spectrometry analysis

Eluates were reduced using 200 mM dithiothreitol at room temperature for 15 min and alkylated using 50 mM iodacetamide at room temperature in the dark for 20 min. The enzymatic digestion ran overnight with 2 µl of (0.5 µg/µl) of sequencing grade trypsin (Promega, V5113) in 50 mM ammonium bicarbonate pH 8 at 37 °C. Digestion was then stopped by adding 2 µl of 100% trifluoracetic acid (TFA). Digested samples were then desalted with C-18 spin columns (Pierce, #89870). Final eluates were dried with a nitrogen evaporator (Microvap 118, Organomation Associates, Inc). Samples were reconstituted in 10 µl of 0.1% Formic Acid. LC–MS/MS was performed on an Orbitrap Fusion Tribrid Mass Spectrometer (Thermo Scientific) coupled with a nanospray EASY-nLC 1200 UHPLC. Reversed-phase chromatography separation of the peptide mixture was performed using PepMap RSLC 75 μm i.d. × 15 cm long with 2 μm, C18 resin LC column (ThermoFisher). 0.1% formic acid as mobile phase A, and 0.1% formic acid, 80% acetonitrile mobile phase B were used. Peptides were eluted using a linear gradient of 5% mobile phase B to 50% mobile phase B in 90 min at 300 nl/min, then to 100% mobile phase B for an additional 2 min. The Thermo Orbitrap Fusion Tribrid Mass Spectrometer (Thermo Scientific) was operated in a data-dependent mode in which each full MS scan was followed by TopN MS/MS scans of the most abundant molecular ions with charge states form 2+ to 4+ were dynamically selected for collision induced dissociation (CID) using a normalized collision energy of 35%. Tandem mass spectra were searched against microorganism databases with Proteome Discoverer 2.1 software using tryptic cleavage constraints. Databases for the following microorganisms were downloaded from NCBI, UniProt, and PiroplasmaDB: *Borrelia burgdorferi, Borrelia mayonii, Borrelia afzelii, Borrelia garinii, Borrelia bissettii, Borrelia spielmani, Borrelia bavariensis, Borrelia hermsii, B. turicatae, B. parkeri, B. miyamotoi, Babesia microti, Francisella tularensis, Ehrlichia chaffeensis, Rickettsia rickettsiae, Rickettsia parkeri, Rickettsia species 364D, Rickettsia conorii, Anaplasma phagocytophilum, Bartonella henselae,* Powassan virus, Tick-borne encephalitis virus, Colorado tick fever virus. In the training phase of the method databases were modified in order to exclude peptide sequences whose spectrum overlaps with sample contaminants.

### Three-tier peptide identification and authentication algorithm

We developed an algorithm to perform peptide authentication, which incorporates stringent filtering criteria in order to minimize the false positive rate. The algorithm includes the following steps:

(A) *Statistical and physical parameters for spectrum matching*. (1) Peptide false discovery rate (FDR) based on forward-reverse decoy < 1%, (2) Xcorr > 2.0, > 3.0 and > 4.0 for 2+, 3+, 4+ precursor ions, (3) q-value < 0.05, (4) precursor ion mass tolerance < 2 ppm and fragment ion mass tolerance < 0.5 Da, (5) If precursor is triply-charged: (5.1) Presence of a basic residue (K, R, H) within the sequence (excluding N-terminus and C-terminus residues), and (5.2) Presence of corresponding doubly charged precursor ion in the full mass chromatogram (MS1).

(B) *Unambiguous peptide attribution to one microorganism*. In order to exclude peptides that share amino acid sequence with other organisms, each peptide attributed to a tick-borne pathogen was subjected to BLAST searches against the NCBI Reference Sequence database (RefSeq)^73^, a comprehensive dataset containing the available protein sequence information for any given species. A peptide showing 100% identity to any additional species included in the RefSeq database beyond the intended tick-borne pathogen was discarded. Peptide sequences shorter than 7 amino acids were discarded in order to minimize random error of attribution when searching for short-peptide sequences^74^. Date and time of BLAST search and database download were recorded.

(C) *Validation of protein database annotation*. The full-length protein, to which every peptide was attributed, was aligned with homologous proteins of evolutionary related organisms in the clade. If the full-length protein had greater than 60% identity with proteins in the query, the database annotation was considered valid.

Attribution of urinary peptides to an organism at the species level was conducted as follows. Full length homologous proteins in related microorganism were aligned using the JalView software. For *Borrelia*, the following species were taken into consideration: *Borrelia burgdorferi*, *Borrelia garinii*, *Borrelia afzelii*, *Borrelia bavariensis*, *Borrelia mayonii*, *Borrelia miyamotoi*, *Borrelia hermsii*, *Borrelia turicatae*, *Borrelia recurrentis*, *Borrelia chilensis*, *Borrelia crocidurae*, *Borrelia duttonii*, *Borrelia bissettii*. A peptide showing 100% identity to a single species and < 90% to other species was attributed to the microorganism at the species level. A peptide showing 100% identity to one or more species and > 90% identity to different species was not attributed to the microorganism at the species level, and all the species with 100% identity were reported (Table S4).

### Targeted peptide identification with parallel reaction monitoring

LC–MS/MS was performed on an Orbitrap Fusion Tribrid Mass Spectrometer (Thermo Scientific) coupled with a nanospray EASY-nLC 1200 UHPLC. Reversed-phase chromatography was performed using PepMap RSLC 75 μm i.d. × 15 cm long with 2 μm, C18 resin LC column (ThermoFisher). Peptides were eluted using a linear gradient of 5% mobile phase B to 50% mobile phase B in 15 min at 300 nl/min, then to 100% mobile phase B for an additional 2 min. The Orbitrap Fusion was operated in data independent acquisition parallel reaction monitoring mode. A targeted list of precursor ions of the peptides of interest AVEIKTLDELK (m/z = 420.24; z = 3), LKNSHAELGVAGNGATTDENAQK (m/z = 775.72; z = 2), NDVSEEKPEIK (m/z = 644.32) were isolated and fragmented by Higher-energy C-trap Dissociation (HCD) with 35% normalized collision and detected at a mass resolution of 60,000. The data were then analyzed using Skyline v3.6 (University of Washington, MacCoss Lab) to determine the presence or absence of peptides of interest.

### Propagation of *Babesia microti* in hamsters

*Babesia microti* GI (BEI Resources NR-44070; ATCC PRA-398) was originally isolated from blood obtained from a human case of babesiosis in Nantucket, Massachusetts, USA, in 1983^74,75^. The isolate was maintained by in vivo propagation in Golden Syrian hamsters (Harlan Laboratories, stock: HsdHan:AURA) according to published protocols^76,77^ and procedures approved by the ATCC IACUC. Ten hamsters were inoculated with ~ 10^8^ parasitized erythrocytes in 0.5 ml of blood. Blood samples were collected by the peri-orbital route following inhalational anesthesia with isoflurane and parasitemia was determined by microscopic examination of Giemsa-stained blood films at different times of infection. A minimum of 500 erythrocytes were counted to calculate the percent parasitemia of each sample. This included all parasitized cells regardless of intraerythrocytic stage or number of parasites per cell. After 30 days of infection, four hamsters (acute group) were anesthetized by ketamine injection (50 mg/kg) and 0.5 ml of blood with and without heparin was collected from each animal. Urine samples (~ 0.1 ml) were collected directly from the bladders with a syringe during abdominal surgery and animals were subsequently euthanized using carbon dioxide inhalation. The six remaining hamsters (chronic group) were monitored for 6 months and blood and urine samples were collected as described above.

### Statistical analysis

Ordinal regression analysis was performed to evaluate correlation between the number of urinary pathogen derived peptides and presence or absence of clinical symptoms in non acute symptomatic patients suspected of tick-borne diseases. Linear regression analysis was performed to evaluate the correlation of the number of *Babesia* derived peptides with parasitemia in the hamster animal model experiment. T-test was used to test the significance of regression. Statistical analyses were performed using SPSS v.19.0 (IBM Corp.). Descriptive statistical analysis of data derived from LD and non acute patients, controls, and hamsters was performed using Python 3 Pandas library and MicrosoftExcel. Visualization were obtained using Python 3 Matplotlib 3.1.1, Seaborn 9.0 libraries and Excel.

## Supplementary information

Supplementary Information.

## Data Availability

All peptide sequences and matching accession numbers reported in this study are made available in the Supplementary Information. Materials used in this study can be obtained by request to A.L.
