# Supplementary material for: Evaluation of pathogen specific urinary peptides in tick-borne illnesses
Source: Sci Rep. 2020 Nov 9;10:19340. doi: 10.1038/s41598-020-75051-3 (PMC7653918; doi:10.1038/s41598-020-75051-3)
Supplement: Supplementary file 1 — Supplementary Information. [file 41598_2020_75051_MOESM1_ESM.docx]

# **Supplementary information for:**

# **Evaluation of pathogen specific urinary peptides in tick-borne illnesses.**

Ruben Magni^a^, Raghad Almofee^a^, Sameen Yusuf^a^, Claudius Mueller^a^, Ngoc Vuong^a^, Mahmood Almosuli^a^, Minh Thu Hoang^a^, Katherine Meade^a^, Ish Sethi^a^, Nuha Mohammed^a^, Robyn Araujo^b^, Teresa Kaza McDonald^a^, Paul Marcelli^a^, Virginia Espina^a^, Brianna Kim^c^, Anja Garritsen^d^, Christine Green^e^, Paul Russo^a^, Weidong Zhou^a^, Iosif Vaisman^a^, Emanuel F. Petricoin III^a^, Deborah Hoadley^f^, Robert E. Molestina^g^, Hope McIntyre^h^, Lance A. Liotta^a^, Alessandra Luchini^a, 1^.

Affiliations:

^a^George Mason University, United States; ^b^Queensland University of Technology, Australia; ^c^Duke University; ^d^Ceres Nanosciences, United States; ^e^Innatoss, The Netherlands; ^f^Medical Offices of Christine Green, United States; ^g^New England Institute for Lyme Disease and Tick-Borne Illness, United States; ^h^American Type Culture Collection, United States; ^i^Lyme Hope, LLC, United States.

^1^To whom correspondence should be addressed: Center for Applied Proteomics and Molecular Medicine, George Mason University, Manassas, VA 20110. Email: [aluchini@gmu.edu](mailto:aluchini@gmu.edu).

**Table of Contents**

**Supplementary Results**…………………………………………..…………………………………….………...……..4

The Affinity Particle – Mass Spectrometry Workflow Attains High Analytical Sensitivity and High Reproducibility.

Babesia microti derived peptides are detected in the urine and blood of an animal model of persistent infection and correlate with parasitemia.

**Supplementary Discussion**.…………………………………………….………………………………….…..……..5

**Supplementary Methods**………………………………………………….……………….……………….…..……..7

Orthogonal confirmation of surface and secreted antigens identified by mass spectrometry, using western blot, Parallel Reaction Monitoring and an animal model of Babesiosis

Western blot analysis for Borrelia proteins

Processing biofluids from Babesia microti

Synthesis of peptide antigens and production of rabbit polyclonal antibodies

Protein extraction for Western blots

Western blot analysis for Babesia proteins

Immunofluorescence analysis

ELISA

Affinity Capture Harvesting: Mathematical model of the biomarker harvesting mechanism

**Supplementary Tables**……………………………………………………………..……………………..…………..13

**Table S1**. Assessment of the analytical sensitivity of the experimental and bioinformatics workflow employed in this study in human urine.

**Table S2.** Demographic and clinical information of acute Lyme borreliosis (acute) and post treatment Lyme disease syndrome (PTLDS) and other non-acute patients suspected of tick-borne illnesses (NA TBI).

**Table S3.** Demographic and disease status of the control samples analyzed in this study.

**Table S4**. Amino acid sequence, protein accession number, protein description, and organism species of all urinary peptides identified in this study.

**Table S5.** List of acute LD and non-acute tick borne disease patients and results of the mass spectrometry analysis.

**Table S6**. Peptides from tick-borne pathogens were identified in pediatric patients suspected of tick-borne illness.

**Table S7.** *Francisella* species and endosymbionts to which seven peptides were attributed.

**Table S8.** Gene Ontology annotation of 109 proteins from *Borrelia* species. Proteins are grouped according to the biological function.

**Table S9**. Gene Ontology annotation of 160 proteins from Borrelia species. Proteins are grouped according to the cellular compartment.

**Table S10:** Number of urinary peptides in PTLDS patients positively correlates with presence or absence of symptoms (p-value <0.025)

**Table S11.** The number of *Babesia* peptides identified in the hamster RBCs correlated with levels of parasitemia.

**Table S12**. List of *Babesia* proteins identified in hamsters infected with *Babesia microti* and their functional annotation.

**Table S13** Seroreactive *Babesia* proteins identified in the blood and urine of a hamster animal model.

**Supplementary Figures**…………………………………………………………….…………………………......………..70

**Fig. S1**. Bioinformatics pipeline for peptide identification and authentication.

**Fig. S2.** Affinity nanoparticle-mass spectrometry experimental workflow is highy reproducible.

**Fig**. **S3**. Statistical power of the authentication algorithm reaches 0.999 at significance level alpha=0.05.

**Fig. S4**. Peptide authentication algorithm reduces the number of false positive peptide identifications.

**Fig. S5**. Distribution of pathogen derived, urinary peptides is equivalent in post treatment Lyme disease syndrome (PTLDS) patients and in non-acute, tick-borne disease (NA TBI) patients.

**Fig. S6**. Biological process and cellular component of Borrelia derived, urinary peptides identified in the urine of acute LB and non-acute patients.

**Fig. S7**. Taxonomy analysis to confirm protein database annotation and peptide attribution to an organism at the species level.

**Fig. S8.** LC-MS/MS CID mass spectrum of an OspA-derived, tryptic peptide captured by affinity hydrogel nanoparticles and detected in the urine of non acute patients.

**Fig. S9**. LC-MS/MS CID mass spectrum of an OspC-derived, tryptic peptide captured by affinity hydrogel nanoparticles and detected in the urine of non acute patients.

**Fig. S10**. LC-MS/MS CID mass spectrum of an OspC-derived, tryptic peptide captured by affinity hydrogel nanoparticles and detected in the urine of non acute patients.

**Fig. S11**. LC-MS/MS CID mass spectrum of a tryptic peptide derived from Protein RecA that was captured by affinity hydrogel nanoparticles and detected in the urine of non acure patients.

**Fig. S12**. LC-MS/MS CID mass spectrum of a tryptic peptide derived from membrane protein insertase YidC [*Bartonella henselae*] that was captured by affinity hydrogel nanoparticles and detected in the urine of non acute patients.

**Fig. S13**. LC-MS/MS CID mass spectrum of a tryptic peptide derived from DNA-directed RNA polymerase subunit beta [*Rickettsia parkeri, Rickettsia conorii, Rickettsia philipii*] that was captured by affinity hydrogel nanoparticles and detected in the urine of non acute patients.

**Fig. S14**. LC-MS/MS CID mass spectrum of a tryptic peptide derived from prolipoprotein diacylglyceryl transferase [*Francisella tularensis*] that was captured by affinity hydrogel nanoparticles and detected in the urine of non acute patients.

**Fig. S15**. Comparison between discovery MS/MS spectrum (above) and PRM showing entire MS/MS spectrum (below) for peptide AVEIKTLDELK (m/z 420.2468 ^3+^; MH+ 1258.726).

**Fig. S16.** Original, uncropped images of western blot membranes shown in Figure 5.

# **Supplementary Results.**

### The Affinity Particle – Mass Spectrometry Workflow Attains High Analytical Sensitivity And High Reproducibility.

Hydrogel particles affinity capture and concentrate low abundance target analytes even when the analytes are bound to a carrier molecule (Supplementary Methods), and effectively increase the analytical sensitivity of mass spectrometry. Analytical sensitivity of the affinity particle / mass spectrometry workflow was 2.5 pg/ml after spiking *Borrelia lysate* in healthy volunteer human despite the presence of urinary endogenous proteins at a concentration more than 8 orders of magnitude greater than *Borrelia* peptides (Supplementary Table S1). The number of unique human peptides identified in each sample was consistently in the range of 4,500 – 5,000. A set of 15 human proteins, known to be generally invariant in the urine^23^, were monitored as a measure of process quality control. The experimental workflow was highly reproducible, as demonstrated by a Pearson correlation score > 0.99, which was calculated via label free quantification of three technical replicates using Peaks Studio (Supplementary Fig. S2).

### *Babesia microti* derived peptides are detected in the urine and blood of an animal model of persistent infection and correlate with parasitemia.

In concordance with the intraerythrocytic nature of *Babesia*, the majority of unique proteins (303/319) were found in the RBC fraction of the blood, nonetheless several proteins were also detected in plasma (6/319), in serum (11/319) and in urine (16/319). Proteins found in hamster body fluids are reported in Fig. 5 and Table S11. The proteomics workflow applied herein was more sensitive than PCR and immunofluorescence in the hamster H5, which had 0% parasitemia with the latter methods but presented with 14 peptides in the urine, 55 peptides in RBCs, and 27 in plasma. Identified peptides were attributed to proteins that serve functions related to ribosomal activity (13%), transport (5%), metabolism (13%), chaperones (4%), transcription (5%), trafficking (2%), DNA replication/recombination/repair (5%), signal transduction (8%), protein translation and modification (9%), biosynthesis (3%), surface/secreted antigens (15%), and motility and invasion (3%); the remaining proteins have not been characterized yet and their function is unknown (15%) (Table S11). Six of the proteins identified in hamster body fluids are among the top 20 immunogenic (IgG or IgM) antigens according to Silva et al. (Table S13). These include GPI-anchored proteins (BmSA1; BmGPI10; BMR1_03g00947), secreted proteins (N1-15 protein, maltese-cross seroactive antigen), and transmembrane proteins (rhoptry neck protein 2)^31,32^. While most of the immunogenic antigens where found in RBCs, serum, plasma, 2/6 proteins (BmSA1, BmGPI10) were also detected in urine of chronic hamsters as further confirmation that urine is a viable body fluid for detection of Babesia antigens in the chronic phase of the infection. Antibodies were raised against BmSA1 and BMR1_03g00947, and additional verification of the mass spectrometry data was obtained by Western blotting (Fig. 5D; Supplementary methods).

# **Supplementary Discussion.**

*Babesiosis* is an emerging zoonotic disease which can be fatal in immunocompromised individuals in the absence of prompt treatment. 17/148 non acute patients were positive for two *Babesia* peptides. We validated our workflow for Babesiosis by investigating biological fluids of hamsters infected with *Babesia microti*. Several of the surface or secreted Babesia antigens identified are known to elicit host immune responses *in vivo*^31^ (i.e., BmSA1, BmGPI10, N1-15 maltese-cross antigen)^31,84^. Several members of the BMN2 family were also detected. These proteins show low immunogenicity and are reported to be involved in the immune evasion^31^. BMN2 family proteins were not only found in RBC but also in urine of chronic hamsters as well as non-acute patients. Within the genus *Babesia*, only *Babesia microti* – the most abundant species in the US - was investigated due to the limitation of available *Babesia* genomes. Nevertheless, our method has the potential to detect also other species such as *Babesia duncani* and *Babesia divergens* which, although less prevalent in the United States and more diffused in Europe, can still pose a significant health risk. Additional tick-borne infections for which we found at least one peptide per single patient are discussed below.

We identified 8 unique peptides from *Rickettsia* species (Supplementary Table S4 and S5) but no patient reached the threshold of two *Rickettsia* peptides. Spotted Fever Rickettsiosis in humans can be caused by several species including *Rickettsia rickettsii*, *Rickettsia parkeri*, *Rickettsia* species 364D, *Rickettsia akari*. Current IFA tests suffer from poor specificity and sensitivity in the early phase of the infection^85^. Moreover, discrimination of different species can be extremely challenging^85^. We also found 15 unique peptides associated to *Bartonella* (Supplementary Table S4 and S5) and 4/148 patient was positive for two Bartonella peptides. Although the possibility of *Bartonella* transmission by ticks still remains controversial^94,95^, no FDA approved test for *Bartonella* is currently available on the market.

*Ehrlichiosis* and *Anaplasmosis* present diagnostic challenges similar to Rickettsiosis. Direct observation of pathogens in peripheral blood is difficult due to low pathogen titers and low specimen cytologic sampling coverage using microscopy reading of IFA. In this study, 12 and 8 unique peptides were identified for Ehrlichia and Anaplasma, respectively. Two patients were positive for two Ehrlichia peptides and one patient for two Anaplasma peptides. (Supplementary Table S4 and S5).

Like other tick-borne illnesses, Tularemia, caused by *Francisella tularensis*, can be difficult to diagnose because it has low prevalence and its symptoms overlap with other infections. Furthermore, culture is difficult and poses a health risk to lab personnel^86^ and the development of sufficient antibody titers might take weeks^87^. In this study, we have found seven peptides that have 100% match with sequences unique of the *Francisellaceae* family and *Francisella*-like endosymbionts (Supplementary Table S7). It is well documented that the presence of phylogenetically-close organisms^88^ such as *Ornithodoros* *moubata*^89^ and *Amblyomma maculatum*^88^ as well as other members of the *Francisellaceae* family characterized by lower^90^ or not yet known virulence^91^ poses a challenge for the identification of *Francisella tularensis*^92,93^. Proteomics might provide more specific diagnostic information to distinguish these commensal organisms from pathogenic species.

Documented cases of TBEV and Powassan virus infections have recently been reported^96,97^. These infections can lead to extremely severe symptoms including fatalities but, fortunately, the number of occurrences is still very low. Conventional tests for these organisms are often non-specific and not easily available to providers^98,99^. No peptide deriving from these viruses was detected in our study.

The unique aspect of the present study is the capability to identify peptides specifically derived from pathogens in the urine of patients without the need of culture amplification. Although MS-based proteomics is becoming more prominent for protein identification, characterization and quantitation, its use in clinical settings for infectious disease testing has been restricted to the use of MALDI-TOF or PCR-Mass Spectrometry analysis after *in vitro* culture of the pathogen isolated from the host ^100,101,102^. These procedures still require time consuming *in vitro* culture of the pathogen. Specifically, in the case of Lyme borreliosis, successful culturing of *Borrelia burgdorferi* using body fluid is very challenging and is hampered by a very low success rate^103^.

# **Supplementary Methods.**

### Orthogonal confirmation of surface and secreted antigens identified by mass spectrometry, using western blot, Parallel Reaction Monitoring and an animal model of Babesiosis

Western blot analysis for Borrelia proteins. Particle eluates were heated at 100°C for 10 minutes. Samples were loaded on a 4-20% Tris-Glycine gel (Invitrogen Corporation) and separated by SDS-PAGE gel electrophoresis. Gel was run in Tris-Glycine SDS running buffer using Novex X-Cell IITM Mini-Cell (Invitrogen Corporation) at 120 V for 90 minutes. Proteins were transferred onto a PVDF membrane (Millipore), blocked with a solution of 0.2% I-Block (Applied Biosciences) and 0.1% Tween 20 (Fisher) in PBS (Life Technologies). The membrane was incubated overnight with monoclonal antibody (mAb) specific for OspA, OspC, or flagellin (Santa Cruz, sc-58093, Clone ID 0551; 200-401-C11S, Rockland ; Novus Biological, 7G11/6802). mAbs were used at a 1:100 dilution in PBS supplemented with I-Block and Tween 20. After mAb incubation, the membrane was washed three times for 10 minutes with 0.2% I-Block, 0.1% Tween 20 in PBS. The membrane was incubated with a peroxidase conjugated goat anti-mouse IgG adsorbed against bovine, equine and human serum proteins (Sigma) diluted 1:5,000 in 0.2% I-Block, 0.1% Tween 20 in PBS. Three washes of 10 minutes in 0.1% Tween 20 in PBS were performed. Proteins were detected with an enhanced chemiluminescence system (Supersignal West Dura, Thermo Fischer Scientific, cut off for detection: mid-femtogram levels of target proteins) on an Azure Imager.

Processing biofluids from Babesia microti. Aliquots of blood, plasma and urine from hamsters infected with *Babesia microti* were inactivated for 20 minutes at 55°C. 500ul of whole blood was centrifuged for 10 minutes at 2000 x g. 500 ul of water was added to the cell pellet and incubated for 45 minutes at room temperature. Red blood cells lysis was facilitated by three freeze and thaw cycle. Samples were centrifuged for 5 minutes at 16,100 x g to eliminate pellets from lysed cells and supernatant was recovered. In parallel, 500ul of plasma was diluted with 500 µl Tris-HCl 50mM pH 7.2 and 200 µL of urine was centrifuged for 10 minutes at 3,750g to eliminate cell particulates and transferred into new tubes. All samples from RBC lysate, plasma and urine were then processed according to the following protocol: samples were incubated with 200 µl affinity particles (10 mg/ml) for 30 minutes at RT. Samples were centrifuged at 16,100 x g for 20 minutes and supernatants were discarded. Pellets were vigorously re-suspended in 1 ml 18 MΩ × cm water and then centrifuged at 16,100 x g for 20 minutes. Supernatant was discarded and pellet was re-suspended in 20 µl of elution buffer solution (4% SDS in in 50 mM NH_4_CO_3_), and incubated for 20 minutes at RT. Samples were centrifuged at 16,100 x g for 20 minutes. Supernatant was saved and transferred into new tubes and 180 µl of 50 mM NH_4_CO_3_ was added. Detergent was removed from the eluates using detergent removal spin columns (Pierce™, cat#87777) according to vendor instructions.

Synthesis of peptide antigens and production of rabbit polyclonal antibodies**.** Rabbit polyclonal antibodies were raised against peptide antigens of the *B. microti* BmSA1 (BMR1_03g00785) and BMR1_03g00947 proteins. The selection of suitable peptide sequences for immunization, synthesis of peptide antigens, and the production of affinity purified antibodies were performed by GenScript Inc. Three peptide antigens with corresponding rabbit antisera were produced for each protein.

Protein extraction for Western blots**.** Protein extracts from infected erythrocytes were prepared by collecting 0.25 ml of blood from a *B. microti* infected hamster with ~40% parasitemia. The blood was mixed with 10 ml ice-cold PBS and centrifuged at 1,100 x g for 5 min at 4^o^C. The erythrocyte pellet was resuspended in 2 ml of 0.5% cold saponin in PBS and incubated for 10 min on ice. The suspension was transferred to a microcentrifuge tube and centrifuged at 1,900 x g for 15 min at 4^o^C. The pellet was resuspended in cold PBS and centrifuged as above for 5 min. This centrifugation step was repeated at least twice until the supernatant was clear. The pellet was resuspended in 0.2 ml of NET buffer (100 mM NaCl, 20 mM Tris-Cl, 1 mM EDTA) with 1% IGEPAL^®^ CA-630 and 1X protease inhibitor cocktail (Sigma). The suspension was incubated on ice for 30 min and centrifuged at 20,000 x g for 20 min at 4^o^C. The supernatant containing parasite extract was stored at -80^o^C until analysis.

Western blot analysis for Babesia proteins**.** Protein extracts were resolved by SDS-PAGE and transferred to polyvinylidene difluoride (PVDF) membranes. Membranes were blocked in PBS with 3% non-fat milk powder and 0.05% Tween 20 for 1 h. Membranes were then probed with 1:500 dilutions of rabbit polyclonal antibodies against peptide antigens of the BmSA1 (BMR1_03g00785) or BMR1_03g00947 proteins. Primary antibody binding was detected by a goat-anti-rabbit antibody conjugated to horseradish peroxidase (HRP) (1:2000 dilution; ThermoFisher). Signals were detected using a chemiluminescence substrate and the Azure c600 Imaging System.

Immunofluorescence analysis**.** A 0.5 ml aliquot of hamster blood with ~40% parasitemia was washed in 10 ml of PBS by centrifugation at 1,100 x g for 5 min. Erythrocytes were fixed in 4% paraformaldehyde and 0.0075% glutaraldehyde in PBS for 30 min as described (18). Fixed cells were washed in PBS as above and permeabilized with 0.1% Triton X-100/PBS for 10 min. Following another PBS wash, cells were blocked in 3% bovine serum albumin in PBS for 1 h. Primary rabbit antibodies to BmSA1 or BMR1_03g00947 were added to the cell suspension at final dilutions of 1:500 and incubated for 1 h at room temperature. Cells were washed three times in PBS and incubated for 1 h with Alexa Fluor^®^ 488-conjugated goat anti-rabbit secondary antibodies (Thermo Fisher) diluted 1:2000 in 3% BSA/PBS. Cells were suspended in mounting medium with DAPI nuclear counterstain and protein localization was visualized under 1000X magnification using a Zeiss Axioscope fluorescence microscope connected to a digital camera. Digital microscopic images were captured using Zen Imaging Software (Zeiss).

ELISA. Peptide antigens of BmSA1 and BMR1_03g00947 were diluted in PBS to a final concentration of 10 μg/ml. Each well of a 96-well microtiter plate was coated with 50 μl of peptide suspension overnight at 4°C. Liquid from the wells was aspirated and the plate was blocked for 30 min at room temperature with 5% non-fat milk in PBS with 0.05% Tween 20. Subsequently, the plates were incubated for 1 h with 100 μl of hamster serum diluted 1:200 in blocking buffer. After washing three times with PBS/0.05% Tween 20, secondary goat anti-hamster IgG antibody conjugated to HRP (1:3000 dilution; Thermo Fisher) was added into each well and incubated for 1 h at room temperature. The wells were washed again and the plate was incubated with 50 μl of peroxidase substrate (Sera Care) for up to 20 min until the development of a deep blue color. The enzyme-substrate reaction was stopped with 50 μl of 1N HCl and the absorbance was measured at 450 nm with a SpectraMax microplate reader (Molecular Devices). Data acquisition was performed with SoftMax Pro software and statistical analysis was performed with GraphPad Prism. One way ANOVA followed by Tukey’s multiple comparison test was performed on data collected from a minimum of three assays. A *P* of <0.05 was used to determine statistical significance for all analyses.

### Affinity Capture Harvesting: Mathematical model of the biomarker harvesting mechanism

The feasibility of using custom-designed affinity bait nanoparticles to harvest and sequester very low abundance urinary biomarkers, even though the biomarker is complexed with high abundance carrier proteins (e.g. urinary uroglobulins), can be demonstrated by the following mathematical analysis.

In the absence of a harvesting high-affinity bait molecule, the binding interactions between a free (uncomplexed) biomarker, *b*, a high-abundance urinary carrier protein, *C*, are described by the following reaction scheme

$b+C {}_{\overset{\leftarrow}{k-}}^{\underset{\to}{k+}}{bC}$,

where *bC* is the bound form of the biomarker, and where *k_+_* and *k_–_* are the forward (binding) and reverse (unbinding) rate constants for the reaction, respectively.

Since the carrier protein exists in such vast excess over the biomarker, its concentration, [C], may be considered a constant (ie. [C] + [bC] ≈ [C]). This very large constant, [C], may therefore be absorbed into the forward rate constant, ***thereby greatly amplifying it***. The equilibrium ratio is then given by

$$\frac{[bC]}{[b]}=\frac{k_{+}^{*}}{k_{-}}=K_{C}, (Eq. 1)$$

where *k_+_^*^* = [C]*k_+_* is the ‘effective’ (amplified) forward rate constant. The parameter *K_C_* is thus the effective affinity constant for the biomarker and carrier protein. Moreover, it follows that the proportion of biomarker in the bound form at steady-state is

$$\gamma_{C}= \frac{[bC]}{\left[ b \right]+[bC]}= \frac{K_{C}}{1+K_{C}}. (Eq. 2)$$

We have previously shown *(1)* that the high abundance of many resident proteins in body fluids (such as albumin in blood) can give rise to very large effective affinity constants (*K_C_* >>1) ***even for biomarkers with exceedingly low affinity for the resident proteins in question***. For instance, considering albumin as the carrier protein, with a blood concentration of 6x10^-4^ mol/L *(2)*, the very low-affinity binding constants (*k_+_*/*k_–_*) of 15x10^4^ L/mol (reported for flavonoid interaction with albumin *(3)*) and 9.34x10^6^ L/mol (reported for interactions between protoporphyrins and albumin *(4)*) correspond to effective affinity constants of *K_C_* = 90 (flavonoids) and *K_C_* = 5600 (protoporphyrins). This in turn corresponds to a probability of 98.9% (flavonoids) and 99.98% (protoporphyrins) that the molecule will exist in complexed form with albumin rather than free in solution.

This analysis demonstrates that although many biomarkers of clinical interest may be present in exceedingly low concentrations in a biofluid, the highly abundant resident proteins in that biofluid can readily conceal a vast reservoir of the biomarker that is several orders of magnitude higher in concentration than that of the free form in solution.

We can now consider how the addition of custom-designed nanoparticles containing a high-affinity bait will not only sequester the ‘free’ biomarker from the biofluid – in this case, urine - ***but but also rapidly harvest the vastly larger ‘hidden’ biomarker reservoir attached to urinary carrier proteins***. The reaction scheme involving the biomarker, *b*, the resident urinary carrier proteins, *C*, and the high-affinity bait, *H*, is depicted as follows


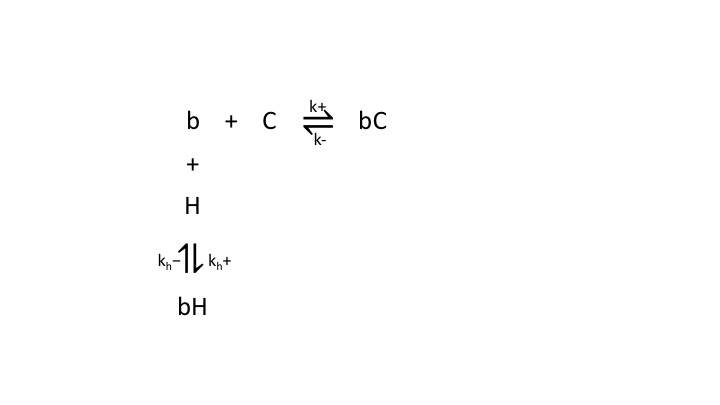


where *k_h+_* and *k_h–_* are the forward (binding) and reverse (unbinding) rate constants for the interactions between the biomarker and the harvesting bait. Note that these rate constants account for the rate of biomarker transit through the particle pores in addition to the affinity of the biomarker for the harvesting bait within the nanoparticle.

Here again, the harvesting baits are present in such vast excess over the low abundance biomarker that their (very large) concentration may be absorbed into the forward rate constant. That is,

$$\frac{[bH]}{[b]}=\frac{k_{h+}^{*}}{k_{h-}}=K_{H}, (Eq. 3)$$

where *k_h+_^*^* = [H]*k_h+_* is the ‘effective’ (amplified) forward rate constant, and *K_H_* is the effective affinity constant for the biomarker and harvesting bait.

The proportion of biomarker bound to the harvesting bait at steady-state is now given by

$$\gamma_{H}= \frac{[bH]}{\left[ b \right]+\left[ bC \right]+[bH]}= \frac{K_{H}}{1+K_{C}+K_{H}}\approx\frac{K_{H}}{K_{C}+K_{H}}, (Eq. 4)$$

noting from our earlier argument that *K_C_* >> 1. Now we note that within each nanoparticle the number of bait molecules is extremely large; in addition, the bait itself is selected to have a very high innate affinity for the biomarker (in stark contrast to the resident urinary proteins, whose innate affinity for the biomarker is very low). From this it follows that *K_H_* >> *K_C_*. Thus, if we assume – conservatively – that the effective affinity of the harvesting bait is 1,000-fold greater than that of the resident urinary proteins, then $\gamma_{H}$ ≈ 0.999, which means that 99.9% of all biomarker will be bound to the harvesting bait at steady-state.

To explain the mechanism by which the vast ‘hidden’ reservoir of biomarker (bound to resident carrier proteins) is fully harvested by the bait molecules inside the nanoparticles, we consider the time-dependent problem. The reaction rate equations for the system are

$$\frac{d\left[ b \right]}{dt}= -\left( k_{+}^{*}+k_{h+}^{*} \right)\left[ b \right]+ k_{-}\left[ bC \right]+k_{h-}\left[ bH \right], (Eq.5)$$

$$\frac{d\left[ bC \right]}{dt}= k_{+}^{*}\left[ b \right]- k_{-}\left[ bC \right], (Eq. 6)$$

$$\frac{d\left[ bH \right]}{dt}= k_{h+}^{*}\left[ b \right]- k_{h-}\left[ bH \right]. (Eq. 7)$$

At *t = 0*, the nanoparticles containing the high-affinity harvesting bait are introduced to the system, at which time an equilibrium is assumed to exist between the free biomarker, *b*, and the biomarker-carrier protein complex, *bC*. Denoting the total biomarker concentration in the system by $\beta,$ we have the following initial conditions

$$\left[ b \right]\left( 0 \right)= \frac{\beta}{1+K_{C}} , (IC. 1)$$

$$\left[ bC \right]\left( 0 \right)= \frac{K_{C}\beta}{1+K_{C}} , (IC. 2)$$

$$\left[ bH \right]\left( 0 \right)=0 . (IC. 3)$$

We illustrate below the solutions to Eqs (5-7), subject to initial conditions (1-3), with the parameter choices $K_{C}$ = 100 ($k_{+}^{*}$=10, $k_{-}$=0.1) and $K_{H}$ = 100,000 ($k_{h+}^{*}$=10,000, $k_{h-}$=0.1), corresponding to a harvesting bait with a 1000-fold higher effective affinity for the biomarker of interest than that of the resident urinary carrier proteins. (We choose $\beta$=100, so that all concentrations will be expressed as a percentage of total biomarker.)

As shown, the biomarker initially exists almost completely in complexed association with the highly abundant proteins in the urine, rather than in a free, uncomplexed form in solution, since the bound and free-phase biomarkers are in the ratio determined by *Eq, 1*, ie. $\left[ bC \right]=K_{C}[b]$. When the bait-containing nanoparticles are introduced at *t=0*, they immediately begin to sequester the free-phase biomarker, attempting to establish the corresponding ratio, $\left[ bH \right]=K_{H}[b]$, between the bait-complexed and free-phase forms. This removal of free-phase biomarker from the solution perturbs the equilibrium ratio for the natural carrier protein, so that now $K_{C}\left[ b \right]<[bC]$, which in turn generates a driving force for the transfer of biomarker from the natural carrier protein to the free form. This transfer sponsors the continued binding of free biomarker to the harvesting affinity nanoparticles. Biomarker transfer from the natural carrier protein to the harvesting particle via the free-phase form will continue in this way until an overall equilibrium is reached, with $\left[ bH \right]=K_{H}[b]$ and $\left[ bC \right]=K_{C}[b]$, at which stage, the proportion of biomarker bound to the harvesting particle will be${K_{H}}/{(1+}K_{C}+K_{H})\approx{K_{H}}/(K_{C}+K_{H})$, as previously described.

Thus, the greater the biomarker affinity for the harvesting particle, $K_{H}$, in comparison with its affinity for the resident urinary carrier protein, $K_{C}$, the closer this ratio will be to unity (ie. *all* biomarker in complexed association with the harvesting particle). For this study, nanoparticle bait chemistries were chosen to achieve virtually a 100 percent capture and a >95% yield of captured biomarker molecules, within the assay limits of detection.

References

(1) Araujo, R.P., Petricoin, E.F. and Liotta, L.A., 2008. Critical dependence of blood-borne biomarker concentrations on the half-lives of their carrier proteins. *Journal of theoretical biology*, *253*(3), pp.616-622.

(2) Corot, C., Violas, X. and Robert, P., 2003. Comparison of different types of blood pool agents (P792, MS325, USPIO) in a rabbit MR angiography-like protocol. *Investigative radiology*, *38*(6), pp.311-319.

(3) Dufour, C. and Dangles, O., 2005. Flavonoid–serum albumin complexation: determination of binding constants and binding sites by fluorescence spectroscopy. *Biochimica et Biophysica Acta (BBA)-General Subjects*, *1721*(1-3), pp.164-173.

(4) Ding, Y., Lin, B. and Huie, C.W., 2001. Binding studies of porphyrins to human serum albumin using affinity capillary electrophoresis. *Electrophoresis*, *22*(11), pp.2210-2216.

# **Supplementary Tables**

**Table S1**. Assessment of the analytical sensitivity of the experimental and bioinformatics workflow employed in this study in human urine. Affinity nanoparticle sample pre-processing and mass spectrometry analysis were applied on a dilution curve obtained by spiking *Borrelia burgdorferi* protein lysate at different concentrations (10, 5, 2.5, 1.25, 0.66, 0) in urine from a healthy participant. *Borrelia* burgdorferi peptides were detected at total protein concentrations ≥ 2.5 pg/mL.

| **Borrelia lysate concentration pg/ml** | **Number of Borrelia unique peptides identified** | **Protein identification number** |
| --- | --- | --- |
| 10 | 2 | A0A0H3C3V1 - A0A076UBU7 |
| 5 | 3 | Q9ZH89 - A0A0E1BXP4 - A0A0H3BZK9 |
| 2.5 | 1 | A0A0E1UAV1 |
| 1.25 | 0 | - |
| 0.66 | 0 | - |
| 0 | 0 | - |

**Table S2.** Demographic and clinical information of acute Lyme borreliosis (acute) and post treatment Lyme disease syndrome (PTLDS) and other non acute patients suspected of tick-borne illnesses (NA TBI) .

| **Sample ID - GMU** | **Sample type, acute=1, PTLDS=2, NA TBI=3** | **Sex** | **Age** | **Tick discovered** | **EM-Rash** | **Joint pain** | **Fatigue** | **Fever** | **Facial Palsy** | **Neurological** | **Serology_Lyme** |
| --- | --- | --- | --- | --- | --- | --- | --- | --- | --- | --- | --- |
| 771232 | 3 | 0 | - | - | - | - | - | - | - | - | - |
| 285020 | 3 | 1 | - | - | - | - | - | - | - | - | - |
| 193471 | 3 | 0 | - | - | - | - | - | - | - | - | - |
| 823161 | 3 | 0 | - | - | - | - | - | - | - | - | - |
| 756890 | 2 | 0 | 51 | 1 | 1 | 1 | 1 | 1 | 1 | 1 | - |
| 911453 | 3 | 0 | - | - | - | 1 | 1 | 0 | 0 | 0 | - |
| 723941 | 3 | 0 | 25 | 1 | - | 1 | 1 | 0 | 0 | 1 | - |
| 832374 | 3 | 1 | 63 | - | - | 0 | 1 | 0 | 0 | 1 | - |
| 562845 | 3 | 1 | 54 | 0 | 0 | 1 | 1 | 0 | 1 | 0 | - |
| 468087 | 3 | 1 | - | 0 | - | 1 | 1 | 0 | 0 | 0 | - |
| 781022 | 3 | 0 | 23 | 1 | 0 | 0 | 1 | 0 | 0 | 1 | - |
| 152946 | 3 | 0 | 8 | 1 | 0 | - | - | - | - | - | - |
| 812785 | 3 | 0 | 50 | 1 | 0 | 0 | 1 | 0 | 0 | 0 | - |
| 849011 | 3 | 0 | - | - | - | - | - | - | - | - | - |
| 228321 | 3 | 0 | - | - | - | - | - | - | - | - | - |
| 315854 | 3 | 1 | - | - | - | 1 | 1 | 1 | 0 | 1 | - |
| 732491 | 3 | 0 | 62 | - | - | 1 | 1 | 1 | 0 | 1 | - |
| 599861 | 3 | 0 | - | - | - | - | - | - | - | - | - |
| 917548 | 3 | 1 | - | - | - | - | - | - | - | - | - |
| 432450 | 3 | 0 | - | - | - | 0 | 1 | 0 | 0 | 1 | - |
| 365481 | 2 | 0 | - | - | 1 | 1 | 1 | 1 | 0 | 1 | 0 |
| 923856 | 3 | 1 | - | - | - | 0 | 1 | 0 | 0 | 0 | - |
| 531792 | 3 | 1 | - | - | - | - | - | - | - | - | - |
| 820444 | 2 | 0 | 33 | 0 | 1 | 0 | 1 | 0 | 0 | 0 | - |
| 823733 | 2 | 0 | - | 0 | 1 | 1 | 1 | 1 | 0 | 1 | 0 |
| 245023 | 2 | 1 | 12 | 0 | 0 | 1 | 0 | 0 | 0 | 0 | 1 |
| 994721 | 3 | 1 | - | - | - | - | - | - | - | - | - |
| 242666 | 3 | 1 | 46 | 0 | 0 | 1 | 0 | 0 | 0 | - | 0 |
| 238401 | 2 | 0 | 48 | 0 | 0 | 1 | 0 | 0 | 0 | 1 | 1 |
| 639032 | 3 | 1 | - | - | - | - | - | - | - | - | - |
| 114343 | 3 | 0 | 38 | 0 | 0 | 1 | 1 | 0 | 0 | 1 | 0 |
| 790907 | 1 | 0 | 61 | 1 | 1 | 1 | 1 | 1 | 0 | 0 | 1 |
| 889597 | 1 | 1 | 44 | 1 | 1 | 1 | 1 | 1 | 0 | 0 | 1 |
| 645608 | 2 | 1 | 64 | 1 | 1 | - | - | - | - | - | 1 |
| 682141 | 3 | 0 | 35 | 0 | 0 | - | - | - | - | - | 0 |
| 887691 | 3 | 1 | 33 | 0 | 0 | - | - | - | - | - | 0 |
| 453742 | 1 | 0 | 57 | 0 | 1 | 0 | 0 | 0 | 0 | 0 | 1 |
| 265499 | 3 | 1 | 58 | 1 | 0 | - | - | - | - | - | 0 |
| 893649 | 2 | 1 | 44 | 0 | 1 | 0 | 0 | 0 | 0 | 1 | 1 |
| 229879 | 2 | 0 | 63 | 1 | 1 | - | - | - | - | - | 1 |
| 460722 | 3 | 1 | 43 | 0 | 0 | - | - | - | - | - | 0 |
| 362346 | 3 | 0 | 59 | - | - | - | - | - | - | - | 1 |
| 687321 | 3 | 1 | 53 | - | - | - | - | - | - | - | 1 |
| 748990 | 3 | 0 | 68 | 0 | 0 | - | - | - | - | - | 0 |
| 213567 | 1 | 1 | 52 | 1 | 1 | 1 | 1 | 1 | 0 | 0 | 1 |
| 890217 | 3 | 1 | 63 | - | - | - | - | - | - | - | 1 |
| 964233 | 3 | 0 | 50 | 0 | 0 | - | - | - | - | - | 0 |
| 923481 | 3 | 1 | 53 | 0 | 0 | - | - | - | - | - | 0 |
| 611349 | 3 | 0 | 42 | 1 | 0 | - | - | - | - | - | - |
| 906453 | 3 | 0 | 12 | 1 | 0 | - | - | - | - | - | - |
| 534390 | 3 | 1 | 72 | - | - | - | - | - | - | - | 0 |
| 906534 | 3 | 1 | 52 | - | - | - | - | - | - | - | 1 |
| 463256 | 1 | 0 | 57 | 0 | 1 | 0 | 0 | 0 | 0 | 0 | 1 |
| 459235 | 1 | 0 | 57 | 0 | 1 | 0 | 0 | 0 | 0 | 0 | 1 |
| 634958 | 3 | 0 | 38 | 0 | 0 | - | - | - | - | - | 0 |
| 441278 | 3 | 1 | 28 | 0 | 0 | - | - | - | - | - | 0 |
| 987783 | 3 | 0 | 55 | 0 | 0 | - | - | - | - | - | 0 |
| 436842 | 2 | 0 | 57 | 0 | 1 | 0 | 0 | 0 | 0 | 0 | 1 |
| 324311 | 3 | 0 | - | - | - | - | - | - | - | - | - |
| 522431 | 3 | 0 | - | - | - | - | - | - | - | - | - |
| 123945 | 2 | 0 | 32 | 0 | 1 | 1 | 1 | 1 | 0 | 1 | 0 |
| 348119 | 3 | 1 | - | - | - | - | - | - | - | - | - |
| 775488 | 3 | 0 | 34 | - | - | - | - | - | - | - | - |
| 732902 | 3 | 0 | - | - | - | 1 | 1 | 1 | 0 | 1 | - |
| 104821 | 1 | 1 | 49 | 1 | 1 | 1 | 1 | 1 | 0 | 1 | 1 |
| 269504 | 3 | 1 | 2 | - | - | - | - | - | - | - | - |
| 108061 | 3 | 0 | - | - | - | - | - | - | - | - | - |
| 108962 | 3 | 1 | 65 | - | - | - | - | - | - | - | - |
| 109126 | 3 | 1 | 17 | - | - | - | - | - | - | - | - |
| 109366 | 3 | 1 | - | - | - | - | - | - | - | - | - |
| 643508 | 3 | 1 | - | - | - | - | - | - | - | - | - |
| 843502 | 3 | 0 | - | - | - | - | - | - | - | - | - |
| 105158 | 3 | 0 | 25 | - | - | - | - | - | - | - | - |
| 108369 | 2 | 1 | - | - | 1 | - | - | - | - | - | 1 |
| 343143 | 3 | 1 | - | - | - | - | - | - | - | - | - |
| 435576 | 3 | 1 | - | - | - | - | - | - | - | - | - |
| 196390 | 3 | 1 | 53 | - | - | 1 | 1 | 1 | 0 | 1 | - |
| 783249 | 3 | 0 | - | - | - | 1 | 1 | 1 | 0 | 1 | - |
| 214378 | 3 | 1 | - | - | - | 1 | 1 | 1 | 0 | 0 | - |
| 729340 | 1 | 0 | - | 0 | 1 | 1 | 1 | 1 | 0 | 1 | 1 |
| 108571 | 2 | 0 | - | - | 1 | - | - | - | - | - | 1 |
| 837682 | 3 | 1 | - | - | - | - | - | - | - | - | - |
| 108294 | 3 | 1 | - | - | - | - | - | - | - | - | - |
| 108785 | 3 | 1 | - | - | - | - | - | - | - | - | - |
| 354291 | 2 | 0 | - | - | 1 | 1 | 1 | 0 | 0 | 1 | - |
| 908230 | 2 | 0 | 40 | 1 | - | 1 | 1 | 1 | 0 | 1 | 1 |
| 108787 | 3 | 1 | - | - | - | - | - | - | - | - | 1 |
| 485701 | 2 | 1 | - | - | 1 | 1 | 1 | 1 | 0 | 1 | - |
| 108786 | 3 | 0 | - | - | - | - | - | - | - | 1 | 1 |
| 739123 | 2 | 1 | - | - | 1 | 1 | 0 | 0 | 0 | 1 | - |
| 343222 | 3 | 0 | - | - | - | - | - | - | - | - | - |
| 108124 | 3 | 0 | - | - | - | - | - | - | - | - | - |
| 788653 | 3 | 0 | - | - | - | - | - | - | - | - | - |
| 108512 | 2 | 0 | - | - | 1 | - | - | - | - | - | 1 |
| 108838 | 1 | 0 | 53 | 1 | 1 | 1 | 1 | 1 | 1 | 1 | 1 |
| 990543 | 3 | 1 | - | - | - | - | - | - | - | - | - |
| 3504713 | 3 | 1 | - | - | - | - | - | - | - | - | - |
| 178546 | 3 | 1 | - | - | - | - | - | - | - | - | - |
| 100981 | 3 | 1 | - | - | - | - | - | - | - | - | - |
| 102424 | 3 | 0 | - | - | - | - | - | - | - | - | - |
| 829147 | 3 | 0 | - | - | - | - | - | - | - | - | 1 |
| 810141 | 3 | 1 | - | - | - | - | - | - | - | - | - |
| 108319 | 3 | 0 | - | - | - | - | - | - | - | - | - |
| 108853 | 2 | 1 | 37 | 1 | 1 | 0 | 1 | 0 | 0 | 1 | - |
| 324344 | 3 | 0 | 69 | - | - | - | - | - | - | - | - |
| 102346 | 3 | 0 | - | - | - | - | - | - | - | - | - |
| 310741 | 1 | 1 | 38 | 1 | 1 | 1 | 1 | 1 | 0 | 1 | 1 |
| 881776 | 3 | 0 | - | - | - | - | - | - | - | - | 1 |
| 900019 | 3 | 1 | - | - | - | - | - | - | - | - | - |
| 974321 | 3 | 1 | - | - | - | - | - | - | - | - | - |
| 671129 | 3 | 1 | - | - | - | - | - | - | - | - | - |
| 964452 | 3 | 1 | - | - | - | - | - | - | - | - | 1 |
| 243325 | 3 | 0 | - | - | - | - | - | - | - | - | - |
| 1121119 | 3 | 0 | - | - | - | - | - | - | - | - | - |
| 562445 | 2 | 1 | 82 | - | 1 | - | - | - | - | - | 1 |
| 623456 | 3 | 0 | - | - | - | - | - | - | - | - | - |
| 980000 | 3 | 0 | - | - | - | - | - | - | - | - | - |
| 453653 | 3 | 0 | - | - | - | - | - | - | - | - | - |
| 648842 | 3 | 1 | - | - | - | - | - | - | - | - | - |
| 753564 | 3 | 1 | - | - | - | 1 | 1 | 1 | 1 | - | 1 |
| 163525 | 3 | 1 | - | - | - | - | - | - | - | - | - |
| 865111 | 3 | 1 | - | - | - | - | - | - | - | - | - |
| 899012 | 3 | 0 | - | - | - | - | - | - | - | - | - |
| 542019 | 3 | 1 | - | - | - | - | - | - | - | - | - |
| 816529 | 2 | 1 | - | 1 | 1 | 1 | 1 | 0 | 1 | 0 | - |
| 891284 | 2 | 0 | - | 1 | 1 | 1 | 1 | 1 | 0 | 1 | 1 |
| 413743 | 3 | 0 | - | - | - | - | - | - | - | - | 1 |
| 985819 | 2 | 1 | - | - | - | 1 | 1 | 1 | - | - | 1 |
| 170103 | 3 | 0 | - | - | - | - | - | - | - | - | 1 |
| 444356 | 2 | 0 | 54 | 0 | 0 | 1 | 1 | 0 | 0 | 1 | 1 |
| 822221 | 3 | 0 | - | - | - | - | - | - | - | - | - |
| 556555 | 3 | 0 | - | - | - | - | - | - | - | - | - |
| 246483 | 3 | 1 | - | - | - | - | - | - | - | - | - |
| 667829 | 2 | 1 | 28 | 1 | 1 | - | - | - | - | - | - |
| 957477 | 2 | 0 | - | - | - | 1 | - | - | - | - | 1 |
| 676765 | 2 | 0 | - | 0 | 1 | - | - | - | - | - | 1 |
| 991873 | 3 | 0 | 67 | - | - | - | - | - | - | - | - |
| 871679 | 2 | 1 | 74 | - | 0 | 1 | 1 | 1 | 0 | 0 | 1 |
| 321456 | 3 | 1 | - | - | - | - | - | - | - | - | - |
| 711111 | 2 | 0 | 63 | - | 1 | - | - | - | - | - | - |
| 887699 | 3 | 1 | 36 | 0 | 0 | 0 | 1 | 0 | 1 | 1 | - |
| 671900 | 3 | 1 | 6 | 1 | 0 | 0 | 0 | 0 | 0 | 0 | - |
| 158492 | 3 | 1 | 9 | 1 | 0 | 0 | 0 | 0 | 0 | 0 | - |
| 882710 | 3 | 0 | - | - | - | - | - | - | - | - | - |
| 886279 | 2 | 1 | - | 0 | 1 | 1 | 1 | 1 | 0 | 0 | - |
| 422311 | 3 | 0 | - | - | - | - | - | - | - | - | - |
| 889992 | 2 | 0 | - | 1 | 0 | 1 | 1 | 0 | 0 | 1 | 0 |
| 742665 | 3 | 1 | 54 | - | - | - | - | - | - | - | - |
| 662221 | 2 | 0 | - | - | - | 1 | - | - | - | - | 1 |
| 711123 | 3 | 0 | 62 | 1 | 0 | 1 | 0 | 0 | 0 | 1 | 0 |
| 280133 | 2 | 1 | 65 | - | 1 | 1 | 1 | 1 | 0 | 1 | 1 |
| 221347 | 3 | 0 | 26 | 0 | 0 | 1 | 1 | 0 | 0 | 1 | - |
| 555444 | 2 | 0 | - | - | - | - | - | - | - | - | - |
| 998999 | 2 | 0 | 42 | 1 | 1 | 1 | 1 | 0 | 0 | 1 | - |
| 226622 | 3 | 0 | 17 | 1 | 0 | 0 | 1 | 0 | 0 | 0 | - |
| 198887 | 2 | 0 | - | 1 | 1 | 1 | 0 | 0 | 1 | 0 | - |
| 111345 | 3 | - | - | - | - | - | - | - | - | - | - |
| 310742 | 3 | - | - | - | - | - | - | - | - | - | - |

**Table S3.** Demographic and disease status of the control samples analyzed in this study. Controls included healthy participants, diseased patients infected by non-tickborne pathogens from Peru, a geographic area with extremely low incidence of tick-borne diseases, and hospitalized US patients diagnosed with traumatic brain injury and acute respiratory distress syndrome.

| **Sample ID** | **Sex** | **Age** | **Health status** |
| --- | --- | --- | --- |
| 555296 | M | 78 | Healthy Control |
| 774584 | M | 78 | Healthy Control |
| 432959 | M | 18 | Healthy Control |
| 315546 | NA | 0.1 | Healthy Control |
| 339893 | NA | 0.1 | Healthy Control |
| 514304 | NA | 0.1 | Healthy Control |
| 860907 | NA | 0.1 | Healthy Control |
| 403413 | NA | 0.1 | Healthy Control |
| 149123 | NA | 0.1 | Healthy Control |
| 471038 | NA | 0.1 | Healthy Control |
| 817386 | NA | 0.1 | Healthy Control |
| 324521 | NA | 0.0 | Healthy Control |
| 466894 | NA | 0.0 | Healthy Control |
| 217271 | NA | 0.0 | Healthy Control |
| 494224 | M | 39 | Healthy control |
| 272335 | M | 44 | Healthy control |
| 270448 | F | 46 | Healthy control |
| 161729 | M | 55 | Healthy control |
| 16581 | F | 50 | Healthy control |
| 22232 | M | 53 | Healthy control |
| 109543 | F | 61 | Healthy control |
| 381072 | F | 60 | Healthy control |
| 764457 | M | 51 | Healthy Control |
| 335827 | M | 29 | Healthy Control |
| 819259 | M | 23 | Healthy Control |
| 21282 | M | 68 | Healthy Control |
| 999204 | M | 68 | Healthy Control |
| 274485 | M | 78 | Healthy Control |
| 929169 | M | 18 | Healthy Control |
| 433160 | M | 8 | Healthy Control |
| 679624 | M | 8 | Healthy Control |
| 737454 | M | 31 | Healthy Control |
| 243633 | M | 31 | Healthy Control |
| 32211 | F | 66 | Healthy Control |
| 84694 | F | 66 | Healthy Control |
| 459141 | M | 37 | Acute respiratory distress syndrome |
| 69449 | M | 37 | Acute respiratory distress syndrome |
| 416220 | M | 37 | Acute respiratory distress syndrome |
| 343935 | M | 69 | Acute respiratory distress syndrome |
| 111223 | M | 69 | Acute respiratory distress syndrome |
| 794453 | M | 69 | Acute respiratory distress syndrome |
| 464975 | F | 53 | Acute respiratory distress syndrome |
| 954443 | F | 53 | Acute respiratory distress syndrome |
| 524832 | F | 53 | Acute respiratory distress syndrome |
| 384827 | F | 53 | Acute respiratory distress syndrome |
| 337290 | M | 37 | Acute respiratory distress syndrome |
| 985343 | M | 37 | Acute respiratory distress syndrome |
| 410982 | M | 37 | Acute respiratory distress syndrome |
| 859866 | F | 62 | Acute respiratory distress syndrome |
| 17964 | F | 62 | Acute respiratory distress syndrome |
| 955912 | NA | 0.1 | Chagas disease |
| 499412 | NA | 0.1 | Chagas disease |
| 643408 | NA | 0.1 | Chagas disease |
| 301259 | NA | 0.0 | Chagas disease |
| 368879 | NA | 0.0 | Chagas disease |
| 622601 | NA | 0.0 | Chagas disease |
| 379048 | NA | 0.0 | Chagas disease |
| 923349 | NA | 0.0 | Chagas disease |
| 824354 | F | 84 | Traumatic Brain Injury |
| 528405 | F | 74 | Traumatic Brain Injury |
| 665440 | F | 84 | Traumatic Brain Injury |
| 844635 | F | 74 | Traumatic Brain Injury |
| 795522 | F | 84 | Traumatic Brain Injury |
| 587331 | M | 32 | Traumatic Brain Injury |
| 861835 | M | 32 | Traumatic Brain Injury |
| 862647 | M | 35 | Traumatic Brain Injury |
| 733726 | M | 35 | Traumatic Brain Injury |
| 116050 | M | 35 | Traumatic Brain Injury |
| 443541 | M | 20 | Traumatic Brain Injury |
| 494580 | M | 20 | Traumatic Brain Injury |
| 153158 | M | 20 | Traumatic Brain Injury |
| 986369 | M | NA | Traumatic Brain Injury |
| 870955 | M | NA | Traumatic Brain Injury |
| 722463 | M | NA | Traumatic Brain Injury |
| 821187 | M | 23 | Traumatic Brain Injury |
| 722581 | M | 23 | Traumatic Brain Injury |
| 345362 | M | 39 | Traumatic Brain Injury |
| 89413 | M | 39 | Traumatic Brain Injury |
| 110932 | M | 31 | Traumatic Brain Injury |
| 8191 | M | 31 | Traumatic Brain Injury |
| 755728 | M | 42 | Traumatic Brain Injury |
| 760773 | M | 42 | Traumatic Brain Injury |
| 169262 | M | 42 | Traumatic Brain Injury |
| 79792 | M | 21 | Traumatic Brain Injury |
| 844095 | M | 21 | Traumatic Brain Injury |
| 968871 | M | 21 | Traumatic Brain Injury |
| 15677 | M | 21 | Traumatic Brain Injury |
| 391738 | M | 67 | Traumatic Brain Injury |
| 500080 | M | 67 | Traumatic Brain Injury |
| 957176 | M | 67 | Traumatic Brain Injury |
| 156388 | M | 52 | Traumatic Brain Injury |
| 36456 | M | 52 | Traumatic Brain Injury |
| 637513 | M | 52 | Traumatic Brain Injury |
| 520414 | F | 58 | Traumatic Brain Injury |
| 175462 | F | 57 | Traumatic Brain Injury |
| 240826 | M | 55 | Traumatic Brain Injury |
| 528720 | M | 55 | Traumatic Brain Injury |
| 714311 | M | 68 | Traumatic Brain Injury |
| 443759 | F | 57 | Traumatic Brain Injury |
| 719220 | M | 55 | Traumatic Brain Injury |
| 709593 | M | 55 | Traumatic Brain Injury |
| 489335 | F | 41 | Traumatic Brain Injury |
| 989359 | F | 57 | Traumatic Brain Injury |
| 17339 | F | 41 | Traumatic Brain Injury |
| 410387 | F | 41 | Traumatic Brain Injury |
| 862813 | M | 48 | Traumatic Brain Injury |
| 303813 | M | 48 | Traumatic Brain Injury |
| 476444 | M | 48 | Traumatic Brain Injury |
| 400743 | M | 32 | Traumatic Brain Injury |
| 933442 | M | 32 | Traumatic Brain Injury |
| 781449 | M | 36 | Traumatic Brain Injury |
| 781173 | M | 36 | Traumatic Brain Injury |
| 186955 | M | 36 | Traumatic Brain Injury |
| 716786 | F | 31 | Traumatic Brain Injury |
| 654143 | F | 31 | Traumatic Brain Injury |
| 244095 | F | 31 | Traumatic Brain Injury |
| 136712 | M | 65 | Traumatic Brain Injury |
| 115494 | M | 65 | Traumatic Brain Injury |
| 399389 | M | 65 | Traumatic Brain Injury |
| 515199 | F | 19 | Traumatic Brain Injury |
| 556532 | F | 19 | Traumatic Brain Injury |
| 276462 | F | 19 | Traumatic Brain Injury |
| 350643 | M | 69 | Traumatic Brain Injury |
| 209949 | M | 69 | Traumatic Brain Injury |
| 50522 | M | 69 | Traumatic Brain Injury |
| 978291 | F | 26 | Traumatic Brain Injury |
| 670917 | F | 26 | Traumatic Brain Injury |
| 698169 | M | 69 | Traumatic Brain Injury |
| 393326 | M | 69 | Traumatic Brain Injury |
| 524749 | M | 19 | Traumatic Brain Injury |
| 603410 | M | 19 | Traumatic Brain Injury |
| 341832 | M | 19 | Traumatic Brain Injury |
| 5535 | M | 24 | Traumatic Brain Injury |
| 833924 | M | 24 | Traumatic Brain Injury |
| 740117 | M | 75 | Traumatic Brain Injury |
| 706781 | M | 75 | Traumatic Brain Injury |
| 357878 | M | 75 | Traumatic Brain Injury |
| 116942 | F | 79 | Traumatic Brain Injury |
| 742626 | F | 79 | Traumatic Brain Injury |
| 536578 | NA | NA | Traumatic Brain Injury |
| 768648 | F | 23 | Tuberculosis |
| 641192 | M | 26 | Tuberculosis |
| 350707 | F | 25 | Tuberculosis |
| 858333 | F | 35 | Tuberculosis |
| 798934 | M | 36 | Tuberculosis |
| 559522 | F | 24 | Tuberculosis |
| 216565 | M | 19 | Tuberculosis |
| 351897 | M | 33 | Tuberculosis |
| 716186 | F | 40 | Tuberculosis |
| 717328 | F | 30 | Tuberculosis |
| 469052 | F | 19 | Tuberculosis |
| 785090 | F | 19 | Tuberculosis |
| 377428 | M | 21 | Tuberculosis |
| 983211 | F | 56 | Tuberculosis |
| 433324 | M | 57 | Tuberculosis |
| 779907 | M | 33 | Tuberculosis |
| 266323 | F | 54 | Tuberculosis |
| 66142 | F | 30 | Tuberculosis |
| 604857 | NA | NA | Tuberculosis |
| 295761 | M | 57 | Tuberculosis |
| 184410 | M | 25 | Tuberculosis |
| 65207 | M | 56 | Tuberculosis |
| 603524 | M | 48 | Tuberculosis |
| 423090 | M | 23 | Tuberculosis |
| 930737 | F | 44 | Tuberculosis |
| 591595 | F | 21 | Tuberculosis |
| 357676 | F | 47 | Tuberculosis |
| 958417 | M | 44 | Tuberculosis |
| 879416 | M | 26 | Tuberculosis |
| 121944 | F | 34 | Tuberculosis |
| 631750 | M | 32 | Tuberculosis |
| 63494 | M | 44 | Tuberculosis |
| 738277 | M | 33 | Tuberculosis |
| 841317 | M | 30 | Tuberculosis |
| 481696 | M | 22 | Tuberculosis |
| 29801 | M | 35 | Tuberculosis |
| 229035 | M | 38 | Tuberculosis |
| 383425 | M | 33 | Tuberculosis |
| 22445 | F | 56 | Tuberculosis |
| 320755 | F | 28 | Tuberculosis |
| 451460 | F | 39 | Tuberculosis |
| 661723 | M | 35 | Tuberculosis |
| 520765 | M | 29 | Tuberculosis |
| 305835 | F | 28 | Tuberculosis |
| 33781 | M | 40 | Tuberculosis |
| 103078 | F | 22 | Tuberculosis |
| 203555 | M | 23 | Tuberculosis |
| 945927 | M | 56 | Tuberculosis |
| 34956 | M | 19 | Tuberculosis |
| 249975 | F | 29 | Tuberculosis |
| 831064 | M | 64 | Tuberculosis |
| 808684 | M | 36 | Tuberculosis |
| 747649 | F | 44 | Tuberculosis |
| 886529 | F | 39 | Tuberculosis |
| 905084 | M | 33 | Tuberculosis |
| 452000 | F | 33 | Tuberculosis |
| 797831 | M | 18 | Tuberculosis |
| 371670 | F | 30 | Tuberculosis |
| 230276 | M | 59 | Tuberculosis |
| 193585 | M | 20 | Tuberculosis |
| 266650 | F | 24 | Tuberculosis |
| 525733 | M | 23 | Tuberculosis |
| 716773 | M | 52 | Tuberculosis |
| 395576 | M | 23 | Tuberculosis |
| 319069 | M | 22 | Tuberculosis |
| 519678 | M | 21 | Tuberculosis |
| 407840 | F | 35 | Tuberculosis |
| 694187 | F | 61 | Tuberculosis |
| 401139 | M | 22 | Tuberculosis |
| 291026 | M | 28 | Tuberculosis |
| 187524 | M | 21 | Tuberculosis |
| 668454 | F | 24 | Tuberculosis |
| 51978 | M | 43 | Tuberculosis |
| 219155 | M | 25 | Tuberculosis |
| 243890 | NA | NA | Tuberculosis |
| 479198 | NA | NA | Tuberculosis |
| 773682 | NA | NA | Tuberculosis |
| 624493 | NA | NA | Tuberculosis |
| 330074 | NA | NA | Tuberculosis |
| 402461 | NA | NA | Tuberculosis |
| 452525 | NA | NA | Tuberculosis |
| 473191 | NA | NA | Tuberculosis |
| 141952 | NA | NA | Tuberculosis |
| 864502 | NA | NA | Tuberculosis |
| 318601 | NA | NA | Tuberculosis |
| 75082 | NA | NA | Tuberculosis |
| 962270 | NA | NA | Tuberculosis |
| 444399 | NA | NA | Tuberculosis |
| 593087 | NA | NA | Tuberculosis |
| 736547 | NA | NA | Tuberculosis |
| 359124 | NA | NA | Tuberculosis |
| 774021 | NA | NA | Tuberculosis |
| 993384 | NA | NA | Tuberculosis |
| 176771 | NA | NA | Tuberculosis |
| 984286 | NA | NA | Tuberculosis |
| 893507 | NA | NA | Tuberculosis |
| 775148 | NA | NA | Tuberculosis |
| 285764 | NA | NA | Tuberculosis |
| 776540 | NA | NA | Tuberculosis |
| 996835 | NA | NA | Tuberculosis |
| 210553 | NA | NA | Tuberculosis |
| 969267 | NA | NA | Tuberculosis |
| 138306 | NA | NA | Tuberculosis |
| 11531 | NA | NA | Tuberculosis |
| 965900 | NA | NA | Tuberculosis |
| 9401 | NA | NA | Tuberculosis |
| 161831 | NA | NA | Tuberculosis |
| 626332 | NA | NA | Tuberculosis |
| 811679 | NA | NA | Tuberculosis |
| 62367 | NA | NA | Tuberculosis |

**Table S4**. Amino acid sequence, protein accession number, protein description, and organism species of all urinary peptides identified in this study. All peptides are unambiguously associated with tick-borne organisms previously documented to be pathogenic in humans^115–134^. All the species that have 100% match with the peptide are presented in the column “Pathogenic species presenting 100% match with query”.

| Sample_ID | RefSeq_ID | Protein | Peptide_sequence | Species 100% | Species 95% |
| --- | --- | --- | --- | --- | --- |
| 771232 | WP_011181309 | ABC transporter permease | SFPIFTSNNQTSTAIIPSD | *Bartonella henselae* | |
| 771232 | WP_117375034 | N-acetylmannosamine-6-phosphate 2-epimerase | LLMADISSLEEAINADR | *Borrelia miyamotoi* | |
| 285020 | XP_012649696 | hypothetical protein BmR1_04g05450 | MHISQMTNSILLSFTYSY | *Babesia microti* | |
| 285020 | WP_025434152 | excinuclease ABC subunit UvrA | LSESIETALSV | *Borrelia hermsii* | |
| 285020 | WP_064536561 | hypothetical protein | GNWGGALIQR | *Borrelia hermsii* | |
| 542019 | WP_106019052 | ParA family protein | ESLDLIINR | *Borrelia burgdorferi* | |
| 542019 | WP_014023373 | PTS mannose transporter subunit IIB | VDVVNTELVDDDLLK | *Borrelia sp.* | |
| 542019 | XP_021338817 | Uncharacterized protein YqeH | QSLIIGALAR | *Babesia microti* | |
| 542019 | XP_012647552 | Macrophage erythroblast attacher | ITNDYVHKR | *Babesia microti* | |
| 891284 | XP_012649113 | LCCL domain-containing protein (CCp2) | TEKPSIVTLTLDLMEAYK | *Babesia microti* | |
| 891284 | XP_012647719 | NOC4, UTP19, U3 small nucleolar RNA | SLFYIATSGNISVEESNLYEAK | *Babesia microti* | |
| 413743 | WP_014023195 | DUF685 domain-containing protein | FGGLHTIVDDATILEFQYK | *Borrelia bavariensis* | |
| 413743 | XP_021337885 | hypothetical protein BmR1_04g08630 | VDLNGEHSLDK | *Babesia microti* | |
| 413743 | XP_012650305 | hypothetical protein BmR1_04g08600 | IGFNVANSLAITCTNITINFN | *Babesia microti* | |
| 413743 | XP_012649063 | aarF domain-containing kinase | VAVLEAANLGLLNLNSPVSNYWPMFGHK | *Babesia microti* | |
| 413743 | WP_031558227 | ParA family protein | VFINDLLEPSLK | *Borrelia burgdorferi, Borrelia garinii, Borrelia mayonii, Borrelia bavariensis* | |
| 413743 | WP_020954596 | UTP--glucose-1-phosphate uridylyltransferase | YALQKLMAENK | *Borrelia miyamotoi* | |
| 985819 | WP_106017219 | lipase | EIDNYLLKEELQ | *Borrelia burgdorferi, Borrelia mayonii, Borrelia afzelii, Borrelia bavariensis, Borrelia garinii* | |
| 985819 | WP_008882406 | flagellar hook assembly protein FlgD | MNGVENVFNLNIS | *Borrelia burgdorferi* | |
| 985819 | AAX17020 | hypothetical membrane associated protein | NYDSFTSSIR | *Borrelia hermsii* | |
| 280133 | WP_070258649 | bifunctional DNA | LVMQLDSGAKLVFHMGMSGR | *Anaplasma phagocytophilum* | |
| 444356 | WP_045804957 | transcription elongation factor GreA | GNTDEVVYQIVGEYEADISK | *Ehrlichia chaffeensis* | |
| 822221 | WP_025443599 | chemotaxis protein | FNNLNNDLGEFEFSR | *Borrelia miyamotoi* | |
| 822221 | WP_020732484 | hypothetical protein | IENLNEATLK | *Borrelia hermsii* | |
| 822221 | WP_106022675 | undecaprenyldiphospho-muramoylpentapeptide beta-N-acetylglucosaminyltransferase | DFFKVILGIIK | *Borrelia garinii, Borrelia burgdorferi, Borrelia bavariensis* | |
| 822221 | WP_011180023 | hypothetical protein | GVGLYTIEFEK | *Bartonella henselae* | |
| 556555 | WP_011181350 | SapC family protein | NGFLGLIFAHLMSTHN | *Bartonella henselae, Bartonella quintana* | |
| 667829 | WP_106011467 | hypothetical protein | DFVFAINNLIFTLNNL | *Borrelia burgdorferi, Borrelia mayonii* | |
| 667829 | WP_044147846 | hypothetical protein | IGLDNATLTFENSMK | *Ehrlichia chaffeensis* | |
| 991873 | WP_003020451 | succinylglutamate desuccinylase/aspartoacylase family protein | SYMQRYAYRITQEIIK | *Francisella tularensis* | |
| 991873 | XP_028871981 | uncharacterized protein BXIN_2443 | SITHSNGYCKPCVFANK | *Babesia sp.* | |
| 711111 | WP_011181378 | class I SAM-dependent methyltransferase | VQETLCGQLNLCWPD | *Barbatonella henselae* | |
| 711111 | AFU75021 | penicillin-binding protein | PVATRIEMIDFLSSTLDIDK | *Borrelia afzelii, Borrelia garinii, Borrelia bavariensis* | *Borrelia burgdorferi* |
| 671900 | XP_012649780 | hypothetical protein BmR1_04g05858 | QPINSYISDDAESQINNASNMMYK | *Babesia microti* | |
| 158492 | XP_021337180 | RuvB-like protein 1 (pontin 52) | IAQELSLHDLDTANAKPMSGNDVVSILGQYL | *Babesia microti* | |
| 886279 | WP_020732373 | hypothetical protein | ELATEDEINNATFQLREL | *Borrelia hermsii* | |
| 886279 | WP_144033213 | Variable large protein | NDTPISPEQNTRKATAN | *Borrelia hermsii* | |
| 889992 | WP_143485395 | hypothetical protein | DLQDVASHESGVSDQPA | *Ehrlichia chaffeensis* | |
| 742665 | WP_085065713 | hypothetical protein | YVILINGGDIK | *Rickettsia parkeri* | |
| 711123 | XP_021337280 | hypothetical protein BMR1_01G00446 | HFGEPETYISALALK | *Babesia microti* | |
| 711123 | XP_012647489 | FYVE and coiled-coil domain-containing protein (FCP) | ILELESQNLE | *Babesia microti* | |
| 555444 | WP_014023569 | 1-acyl-sn-glycerol-3-phosphate acyltransferase | LLESGSTIVGMQNILEL | *Borrelia sp.* | *Borrelia burgdorferi* |
| 998999 | WP_044052226 | hypothetical protein | NPYVYLEDFNK | *Borrelia afzelii, Borrelia garinii* | |
| 998999 | XP_021337692 | U4/U6.U5 tri-snRNP-associated protein 1 | QTTSTSDTVSHSPTVVTK | *Babesia microti* | |
| 998999 | WP_025406847 | excinuclease ABC subunit B | SDGQMEDLYNEVQK | *Borrelia hermsii* | |
| 193471 | A0A0A7UWS0 | Uncharacterized protein | ILGYAIEGK | *Borrelia burgdorferi* | |
| 756890 | Q6UR22 | OspC (Fragment) | LKNSHAELGVAGNGATTDENAQK | *Borrelia afzelii* | |
| 756890 | W5SCW8 | Exodeoxyribonuclease V alpha chain | EDANLIDLQLNVNK | *Borrelia miyamotoi* | |
| 756890 | A0A0R9QEI9 | Uncharacterized protein | FHSNALEVQVQAEIEAR | *Borrelia turicatae* | |
| 723941 | Q6UR22 | OspC (Fragment) | LKNSHAELGVAGNGATTDENAQK | *Borrelia afzelii* | |
| 832374 | Q6UR22 | OspC (Fragment) | LKNSHAELGVAGNGATTDENAQK | *Borrelia afzelii* | |
| 562845 | A0A0A7UWS0 | Uncharacterized protein | ILGYAIEGK | *Borrelia burgdorferi* | |
| 781022 | WP_044052160 | hypothetical protein | KIIIKFPEYT | *Borrelia burgdorferi, Borrelia mayonii, Borrelia afzelii, Borrelia bavariensis, Borrelia garinii* | |
| 781022 | AJA90394 | chemotaxis protein | QISILHNSLVK | *Borrelia sp.* | *Borrelia burgdorferi* |
| 781022 | WP_038363604 | WP_038363604 - translation initiation factor IF-2 - [Borrelia persica] | KKENLANPIPK | *Borrelia miyamotoi, Borrelia hermsii, Borrelia turicatae* | |
| 781022 | WP_011179070 | 2'-deoxycytidine 5'-triphosphate deaminase | LPKNLSAVANPK | *Bartonella quintana* | |
| 781022 | WP_111738306 | M23 family metallopeptidase | IIGTPPRNLGTI | *Bartonella quintana* | |
| 781022 | WP_031489998 | redox-regulated ATPase YchF | LNAGIVGLPNVGK | *Borrelia burgdorferi, Borrelia mayonii, Borrelia garinii, Borrelia afzelii, Borrelia bavariensis* | |
| 781022 | WP_025408373 | hypothetical protein | QVKIPLNVNLN | *Borrelia miyamotoi* | |
| 781022 | WP_075552597 | chromosome replication/partitioning protein | NISKQNPIKPL | *Borrelia burgdorferi, Borrelia mayonii* | |
| 152946 | WP_071983715 | excinuclease ABC subunit B | KDNSKLVEAER | *Borrelia sp.* | *Borrelia burgdorferi* |
| 152946 | WP_028328339 | adenylosuccinate synthase | ITAATGCGIPISK | *Borrelia hermsii* | |
| 152946 | WP_012622204 | hypothetical protein | KIENKVGSNAAGT | *Borrelia garinii* | |
| 152946 | WP_029346894 | glutamine-hydrolyzing GMP synthase | DKILKNFVFK | *Borrelia garinii* | |
| 152946 | ABV75938 | multidrug resistance protein A | IGRSSLEQAAEK | *Rickettsia rickettsii* | |
| 152946 | WP_014696474 | PTS glucose transporter subunit IIB | VGTLIYGIFLR | *Borrelia hermsii, Borrelia turicatae, Borrelia duttonii, Borrelia recurrentis* | |
| 812785 | W5SQT1 | Uncharacterized protein | QNGEQSKEVIK | *Borrelia sp.* | *Borrelia duttoni* |
| 812785 | WP_071983560 | exodeoxyribonuclease V subunit alpha | YKKIPTFNLK | *Borrelia mayonii* | |
| 812785 | AHX09459 | putative membrane protein | AALFIGYAVILT | *Ehrlichia chaffeensis* | |
| 812785 | EEH00288 | conserved hypothetical protein (plasmid) | KIDFNKVFIK | *Borrelia sp.* | *Borrelia burgdorferi* |
| 812785 | WP_111738296 | autotransporter outer membrane beta-barrel domain-containing protein | QGIVFLKKATF | *Bartonella quintana* | |
| 812785 | XP_012647923 | hypothetical protein BMR1_02g00745 | LVHLILFTQPA | *Babesia microti* | |
| 812785 | ANA43971 | VlpA70 (plasmid) | VKAAKGAGVPEPK | *Borrelia hermsii* | |
| 812785 | AHH12137 | Phosphatidate cytidylyltransferase | LTFIARLGTFL | *Borrelia hermsii, Borrelia miyamotoi* | |
| 812785 | AJA67228 | uracil-xanthine permease (plasmid) | GIVSILVFEFK | *Borrelia miyamotoi* | |
| 228321 | A0A0A7UYW5 | DNA mismatch repair protein MutS | EILENDADVNVK | *Borrelia sp.* | |
| 315854 | WP_031541693 | septal ring lytic transglycosylase RlpA family protein | KTKQSDENISVENKPK | *Borrelia burgdorferi* | |
| 315854 | W5STS8 | Variable large protein | DTAANKTEAEAAKGVAANAVNK | *Borrelia sp.* | *Borrelia turicatae* |
| 315854 | A0A1D8TDG3 | Uncharacterized protein | KDEAVQMIIKAADVDK | *Borrelia miyamotoi* | |
| 315854 | WP_040236434.1 | Bax inhibitor-1/YccA family protein | MLELTQDK | *Borrelia sp.* | |
| 315854 | WP_045245604 | chemotaxis protein CheB | NETNNLHEEIIDEKAL | *Borrelia hermsii* | |
| 315854 | WP_012737743 | cell surface protein | QQAIDQSKKVGS | *Rickettsia sp.* | |
| 315854 | WP_117374741 | DUF327 family protein | KEQKGGTNGEIK | *Borrelia miyamotoi* | |
| 315854 | WP_119024258 | DNA primase | GGRYLGRNNGPK | *Borrelia turicatae* | |
| 315854 | WP_025407406 | DNA polymerase III subunit beta | FNGGGILKLSDPA | *Borrelia sp.* | *Borrelia turicatae* |
| 315854 | XP_012649006 | TAF9, FAP7, transcription initiation factor TFIID subunit 9 / adenylate kinase | VLNCDIKELGK | *Babesia microti* | |
| 315854 | XP_012650394 | peptide alpha-N-acetyltransferase | LLVTLDVCNEL | *Babesia microti* | |
| 315854 | WP_031541621 | endopeptidase La | KVPLDLAMTGEV | *Borrelia burgdorferi, Borrelia mayonii, Borrelia afzelii, Borrelia garinii, Borrelia bavariensis, Borrelia hermsii, Borrelia miyamotoi* | |
| 315854 | BAA22351 | outer surface protein B, partial | KNDGSGGKLEGVK | *Borrelia garinii* | |
| 315854 | XP_012647924 | Importin-5 | QAVNDSVKNSVK | *Babesia microti* | |
| 732491 | Q6ASN6 | Oligopeptide ABC transporter, periplasmic oligopeptide-binding protein | VNESDLGIKALDEK | *Borrelia garinii, Borrelia afzelii, Borrelia bavariensis* | |
| 732491 | A0A0E1U9X9 | Uncharacterized protein | RNSNSTIIVNNK | *Borrelia burgdorferi* | |
| 599861 | WP_012149474 | apolipoprotein N-acyltransferase | MLNATNAILITGG | *Rickettsia akari* | |
| 599861 | WP_006009763 | 4-hydroxy-tetrahydrodipicolinate synthase | RVPIIAGSSSNST | *Ehrlichia chaffeensis* | |
| 599861 | XP_021337329 | ATP-dependent RNA helicase DHX8/PRP22 | QDKQALSDQKK | *Babesia microti* | |
| 599861 | WP_010889724 | UDP-N-acetylmuramoyl-tripeptide--D-alanyl-D-alanine ligase | ELGELAYKTHK | *Borrelia burgdorferi* | |
| 599861 | WP_141631485 | hypothetical protein, partial | VKANTTAAKGGEAA | *Borrelia sp.* | |
| 599861 | XP_021338144 | T-complex protein 1 subunit alpha | SLPMLSGIIGER | *Babesia microti* | |
| 599861 | WP_156110316 | glycoside hydrolase family 3 protein | EGGLVTRVSENK | *Borrelia miyamotoi* | |
| 599861 | WP_071983718 | arginine deiminase | KVDIIKCAGGDL | *Borrelia sp.* | |
| 365481 | A0A0E0SSC4 | Replicative DNA helicase | CATDKSTNLEEVFDVLQKK | *Borrelia burgdorferi* | |
| 365481 | Q6UR22 | OspC (Fragment) | LKNSHAELGVAGNGATTDENAQK | *Borrelia afzelii* | |
| 365481 | Q49579 | Outer surface protein C | LKDNHAQLGIQNGASLDDEAK | *Borrelia garinii* | |
| 923856 | XP_021337827 | DUSP12, YVH1, dual specificity phosphatase 12 | MNLGDTLQIGSLNFAETHF | *Babesia microti* | |
| 923856 | YP_009363174 | hypothetical protein (apicoplast) | YNNNYIINK | *Babesia microti* | |
| 923856 | WP_011452810 | 6,7-dimethyl-8-ribityllumazine synthase | HHAVPMGFGVITAGSLEIAMAR | *Ehrlichia chaffeensis* | |
| 531792 | A0A0E0SSC4 | Replicative DNA helicase | CATDKSTNLEEVFDVLQKK | *Borrelia burgdorferi* | |
| 123945 | Q0SN88 | His Kinase A domain protein | IDMLNTNTIDSNLDNIFNIK | *Borrelia afzelii* | |
| 123945 | A0A1D8TDG3 | Uncharacterized protein | KDEAVQMIIKAADVDK | *Borrelia miyamotoi* | |
| 123945 | W6THA6 | Putative membrane spanning protein | LGSLSGSEVK | *Borrelia sp.* | |
| 123945 | B5RP22 | Vsp protein | GLETTDNISDKLKR | *Borrelia sp.* | |
| 123945 | EED30070 | ATP-dependent Clp protease, subunit A | DILSEELGIQK | *Borrelia garinii, Borrelia bavariensis* | |
| 123945 | WP_044051985 | hypothetical protein | EIEENLSLIGK | *Borrelia afzelii* | |
| 823733 | A0A218PF23 | VlpB9 | LNELSSEISEAK | *Borrelia turicatae* | |
| 823733 | AGJ81566 | acetate kinase, partial | YIGSYLAILEF | *Borrelia hermsii* | |
| 823733 | WP_080947527 | hypothetical protein | SVVNLPAIDMLT | *Anaplasma phagocytophilum* | |
| 823733 | WP_088895085 | hypothetical protein | EGLTIGNAKLCL | *Borrelia turicatae* | |
| 823733 | XP_021337167 | transcription elongation factor SPT6 | QIVAQLCDTLK | *Babesia microti* | |
| 823733 | XP_012648084 | BMN1-5B | GDVSVSRAQLEK | *Babesia microti* | |
| 823733 | XP_021337985 | conserved Plasmodium protein, unknown function | LDGLVLMNREI | *Babesia microti* | |
| 823733 | WP_002658307 | rRNA maturation RNase YbeY | EGMLILQENIL | *Borrelia burgdorferi, Borrelia mayonii, Borrelia afzelii* | |
| 245023 | B7XS60 | CheA | VYLNNISDLK | *Borrelia garinii* | |
| 245023 | WP_044170479 | hypothetical protein | MGVFLLMLSLLMCVR | *Ehrlichia chaffeensis* | |
| 245023 | WP_024072397 | bifunctional demethylmenaquinone methyltransferase/2 | QNITSGSLLDVASGTGDIAIK | *Ehrlichia sp.* | *Ehrlichia chaffeensis* |
| 994721 | WP_025407311 | tetratricopeptide repeat protein | SAILKISAYDQGNVNFAYYK | *Borrelia sp.* | *Borrelia hermsii, Borrelia miyamotoi* |
| 994721 | XP_021338357 | Protein dopey-1 | LMPCLVGLLLCILPGLEDDK | *Babesia microti* | |
| 994721 | XP_021337625 | plasmepsin V (PMV) | RPEIDNIFSICLSD | *Babesia microti* | |
| 994721 | XP_012648602 | translation initiation factor IF-2 | ILVVAADDGIMPQTVESYK | *Babesia microti* | |
| 242666 | Q6UR22 | OspC (Fragment) | LKNSHAELGVAGNGATTDENAQK | *Borrelia afzelii* | |
| 639032 | WP_044160877 | hypothetical protein | SSLLADDSGGNIAR | *Ehrlichia chaffeensis* | |
| 639032 | WP_117375036 | alpha/beta fold hydrolase | IPNTLEEIDQYL | *Borrelia miyamotoi* | |
| 639032 | XP_012647349 | translation initiation factor EIF-2B subunit related | VSMESADNITTI | *Babesia microti* | |
| 639032 | WP_106022834 | ATP-dependent RNA helicase | EIANVQMQLENIISK | *Borrelia burgdorferi* | |
| 114343 | A0A1L6VVD6 | Porin | DQVNQALDTINKVAEDVSNK | *Borrelia mayonii* | *Borrelia afzelii* |
| 114343 | Q6UR22 | OspC (Fragment) | LKNSHAELGVAGNGATTDENAQK | *Borrelia afzelii* | |
| 790907 | WP_106017067 | immunogenic protein P37 | SNQDNQTLLLSLHQAIAK | *Borrelia burgdorferi* | |
| 790907 | Q6UR22 | OspC (Fragment) | LKNSHAELGVAGNGATTDENAQK | *Borrelia afzelii* | |
| 889597 | A0A1L8Z945 | Uncharacterized protein | LATVNEAEGK | *Borrelia bissettii* | |
| 889597 | 3HJA_A | Chain A, Glyceraldehyde-3-phosphate dehydrogenase | GGYLDHVNHAGAKKVILTVPAK | *Borrelia burgdorferi, Borrelia afzelii, Borrelia garinii, Borrelia mayonii* | |
| 645608 | WP_008882360 | 16S rRNA (cytosine(1402)-N(4))-methyltransferase RsmH | HFLSIFEER | *Borrelia sp* | *Borrelia burgdorferi* |
| 682141 | A0A218PF40 | Uncharacterized protein | IREQTQEEIQNIK | *Borrelia turicatae* | |
| 682141 | WP_081719344 | type 2 isopentenyl-diphosphate Delta-isomerase | IAMPIFISSMTGGVK | *Borrelia turicatae* | |
| 682141 | WP_041078759 | phosphomannomutase/phosphoglucomutase | QFMPAGSIMVTGSHN | *Rickettsia akari* | |
| 682141 | WP_006433983 | competence protein | YTNALNNFFTSL | *Borrelia spielmanii* | *Borrelia burgdorferi* |
| 887691 | A0A1L8Z945 | Uncharacterized protein | LATVNEAEGK | *Borrelia bissettii* | |
| 887691 | WP_012150208 | YdcF family protein | EYNYNMPFMLNK | *Rickettsia akari* | |
| 453742 | WP_008882341 | transcript cleavage factor | VVILNEATGK | *Borrelia sp.* | *Borrelia burgdorferi* |
| 453742 | A0A1L8Z945 | Uncharacterized protein | LATVNEAEGK | *Borrelia bissettii* | |
| 453742 | WP_029346900 | UTP--glucose-1-phosphate uridylyltransferase | FVYIGNVDNMGYTINFK | *Borrelia garinii* | |
| 265499 | G0ANW1 | Uncharacterized protein | NIEEELEKLAEPQNIEDK | *Borrelia burgdorferi* | |
| 265499 | A0A1L8Z945 | Uncharacterized protein | LATVNEAEGK | *Borrelia bissettii* | |
| 893649 | WP_108728866 | aminopeptidase | FMIGSNDINVIGIDK | *Borrelia hermsii, Borrelia turicatae* | |
| 460722 | A0A1D8TDG3 | Uncharacterized protein | KDEAVQMIIKAADVDK | *Borrelia miyamotoi* | |
| 460722 | WP_008882409 | hypothetical protein | DGPVSLAISR | *Borrelia burgdorferi* | |
| 460722 | WP_014023462 | DNA mismatch repair endonuclease MutL | TEDDHLIEMINEPENFNK | *Borrelia sp.* | *Borrelia burgdorferi* |
| 362346 | A0A1D8TDG3 | Uncharacterized protein | KDEAVQMIIKAADVDK | *Borrelia miyamotoi* | |
| 748990 | WP_034447395.1 | molecular chaperone SurA | MTQLQETADGIEAIAVCKIK | *Bartonella henselae* | |
| 748990 | H6PWA1 | DNA-directed RNA polymerase subunit beta | IGEMITADMLNVINDLK | *Rickettsia sp.* | |
| 748990 | BmR1_04g06597 | hypothetical protein | FSSEPDDLTEK | *Babesia microti* | |
| 213567 | A0A1L8Z945 | Uncharacterized protein | LATVNEAEGK | *Borrelia bissettii* | |
| 213567 | WP_014696160 | MULTISPECIES: flagellar motor switch protein FliN | NDVSEEKPEIK | *Borrelia sp* | *Borrelia hermsii* |
| 890217 | WP_011181139.1 | type IV secretion system-coupling protein virD4 | MIQETTPSPFIR | *Bartonella henselae* | |
| 890217 | WP_011181071.1 | membrane protein insertase YidC | LIQPMLLEIK | *Bartonella henselae* | |
| 890217 | BMR1_02g00565 | glycerol-3-phosphate O-acyltransferase | GLGNIIFENTR | *Babesia microti* | |
| 890217 | XP_012649545 | conserved Plasmodium protein, unknown function | DFIVNIEDINPSVYSDSIK | *Babesia microti* | |
| 890217 | WP_151060615 | iron-sulfur cluster assembly scaffold protein | SLENYKPLR | *Borrelia mayonii, Borrelia afzelii* | |
| 964233 | WP_014695942 | signal peptidase I | QVNNSYYLNGNLMIGYK | *Borrelia sp.* | |
| 923481 | H6PWA1 | DNA-directed RNA polymerase subunit beta | IGEMITADMLNVINDLK | *Rickettsia sp.* | |
| 923481 | BMR1_02g00565 | glycerol-3-phosphate O-acyltransferase | GLGNIIFENTR | *Babesia microti* | |
| 923481 | BMR1_03g04565 | Protein kinase domain | LSEEVTEFDNAIAQDK | *Babesia microti* | |
| 923481 | XP_012649617 | mitochondrial ribosomal protein S17 precursor, putative | DEQSQASLGDIVR | *Babesia microti* | |
| 611349 | Q660X6 | DNA polymerase I | ELELIENEIIK | *Borrelia burgdorferi, Borrelia garinii, Borrelia bavariensis, Borrelia afzelii* | |
| 611349 | T1ECH2 | Uncharacterized protein | FEEIKIEAR | *Borrelia turicatae* | |
| 611349 | BMR1_03g04565 | Protein kinase domain | LSEEVTEFDNAIAQDK | *Babesia microti* | |
| 611349 | YP_170186.1 | prolipoprotein diacylglyceryl transferase | YLVLGLFMFLYGCAR | *Francisella tularensis, Francisella hispaniensis* | |
| 611349 | WP_106011477 | DNA topoisomerase (ATP | YMLDNENASIFDLLK | *Borrelia burgdorferi, Borrelia afzelii, Borrelia garinii, Borrelia bavariensis* | |
| 611349 | WP_010883900 | ParA family protein | NYVHNVEIYNTYSMLK | *Borrelia burgdorferi* | |
| 906453 | T1ECH2 | Uncharacterized protein | FEEIKIEAR | *Borrelia turicatae* | |
| 906453 | A0A1D8TDR2 | Peptidoglycan-binding protein LysM | YKMYDIEDSLEMYSK | *Borrelia miyamotoi* | |
| 906453 | BMR1_03g00020 | BMN2 family | NIDESNIEHLK | *Babesia microti* | |
| 111345 | WP_006433838 | PleD family two-component system response regulator | DLVLLDVGLPDINGYEVCR | *Borrelia spielmanii* | |
| 111345 | WP_025406568 | HAD family hydrolase | PEMGINLVMFPEFLK | *Borrelia hermsii* | |
| 534390 | W5SJU8 | tRNA (guanosine(18)-2'-O)-methyltransferase | IVATSLNNQSVSLEDFPIDNK | *Borrelia miyamotoi* | |
| 534390 | A1R0L1 | tRNA(Ile)-lysidine synthase | NNLMPVIK | *Borrelia turicatae* | |
| 906534 | B7XS64 | Uncharacterized protein | LELYIENNVEPIK | *Borrelia garinii* | |
| 463256 | A0A0A7UVW0 | Nicotinate phosphoribosyltransferase | NLSLFTDFYEISMMNAYFIK | *Borrelia spielmanii* | *Borrelia burgdorferi, Borrelia afzelii, Borrelia garinii, Borrelia bavariensis, Borrelia mayonii, Borrelia turicatae* |
| 463256 | WP_145940815.1 | uncharacterized protein | QKATGAINAVSGEQIL | *Borrelia mayonii* | |
| 459235 | WP_008882425 | YigZ family protein | SAKEVINNTSTMEK | *Borrelia sp.* | *Borrelia burgdorferi* |
| 459235 | WP_015026969 | molecular chaperone HtpG | SSSVDGFVSFKEYKER | *Borrelia burgdorferi, Borrelia mayonii, Borrelia garinii, Borrelia bavariensis, Borrelia afzelii* | |
| 634958 | O50616 | Outer surface protein A (Fragment) | GTSDKSNGSGILEGEK | *Borrelia garinii* | |
| 441278 | A0A0E1C250 | Uncharacterized protein | MPLFPIDK | *Borrelia hermsii, Borrelia turicatae* | |
| 441278 | WP_106019189 | hypothetical protein | FDLVLYQYHLVLK | *Borrelia burgdorferi, Borrelia mayonii* | |
| 441278 | WP_110503430 | alanine racemase | IPQNINNNFYFLINNQK | *Borrelia garinii, Borrelia bavariensis* | |
| 441278 | WP_146124657 | DUF244 domain-containing protein, partial | FFPSNIAFANSVSVPM | *Borrelia burgdorferi* | |
| 436842 | A0A0E1C0C2 | Uncharacterized protein | NEIFIPTR | *Borrelia hermsii* | |
| 324311 | Q661Y3 | Flagellar protein | IVGEGNGSVEL | *Borrelia bavariensis* | |
| 324311 | A0A1D8TDG3 | Uncharacterized protein | KDEAVQMIIKAADVDK | *Borrelia miyamotoi* | |
| 324311 | A0A1D8TDQ0 | DNA protecting protein DprA | IYDPPFAIYYK | *Borrelia burgdorferi, Borrelia mayonii, Borrelia garinii, Borrelia afzelii, Borrelia spielmanii, Borrelia miyamotoi, Borrelia hermsii* | |
| 676765 | XP_012648506 | Protein tyrosine kinase | SLDNSNDSTFVIQDVANMIK | *Babesia microti* | |
| 957477 | WP_011452888 | phosphatidylglycerophosphatase A | ISGTTGIILDDVLAG | *Ehrlichia chaffeensis* | |
| 957477 | WP_044170504 | ATP-dependent chaperone ClpB | QPVFVSESTVNDTISILR | *Ehrlichia chaffeensis* | |
| 957477 | WP_006011115 | OmpA family protein | YPDTNIIIVGHTDTR | *Ehrlichia chaffeensis* | |
| 522431 | H6VGK4 | Outer surface protein C | AILKTNGDKTLGAAELEK | *Borrelia burgdorferi, Borrelia afzelii* | |
| 310741 | Q6ASN6 | Oligopeptide ABC transporter, periplasmic oligopeptide-binding protein | VNESDLGIKALDEK | *Borrelia bavariensis, Borrelia garinii, Borrelia afzelii* | |
| 310741 | Q0SN56 | Mechanosensitive ion channel family protein | FNVEACFPTLIVEK | *Borreliella afzelii* | |
| 310742 | Q0SN56 | Mechanosensitive ion channel family protein | FNVEACFPTLIVEK | *Borrelia afzelii* | |
| 348119 | C0AP43 | Transcription elongation factor GreA | VVILNEATGK | *Borrelia sp.* | *Borrelia burgdorferi* |
| 348119 | WP_028328185 | Variable large protein 15/16 | TLNTLIIAIR | *Borrelia turicatae* | |
| 348119 | H6PTL6 | Diaminopimelate epimerase | EENGNIIMQGAATLVAR | *Rickettsia parkeri* | |
| 348119 | BMR1_03g04055 | Babesia microti strain RI | YILDPEDYDSGIYNLKQTLK | *Babesia microti* | |
| 732902 | A1QYV0 | Superoxide dismutase | HHNAYTVNLNSVLEK | *Borrelia hermsii, orrelia turicatae, Borrelia miyamotoi* | |
| 732902 | H6PWA1 | DNA-directed RNA polymerase subunit beta | IGEMITADMLNVINDLK | *Rickettsia sp.* | |
| 732902 | WP_011450270.1 | hypothetical protein | FSHEAVVTR | *Anaplasma phagocytophilum* | |
| 104821 | H6VGK4 | Outer surface protein C | AILKTNGDKTLGAAELEK | *Borrelia afzelii, Borrelia burgdorferi* | |
| 104821 | WP_075552323 | Chemotaxis CheA | NNFCLSKSDLEEIR | *Borrelia mayonii* | |
| 881776 | XP_012647479 | actin II | EAPEIIFNPHLNNK | *Babesia microti* | |
| 881776 | BAA82630 | flagellin protein | GAQQEGAQQPAPA | *Borrelia burgdorferi, Borrelia mayonii, Borrelia bavariensis, Borrelia garinii* | |
| 881776 | BAA19222 | Outer surface protein A | NISKSGEITVALDDTASAN | *Borrelia garinii* | |
| 881776 | Q49579 | Outer surface protein C | LKDNHAQLGIQNGASLDDEAK | *Borrelia garinii* | |
| 108061 | WP_031541693 | septal ring lytic transglycosylase RlpA family protein | KTKQSDENISVENKPK | *Borreliella burgdorferi* | |
| 108061 | W6TH26 | Uncharacterized protein | ELETINTIK | *Borrelia sp., Borrelia duttoni* | |
| 108962 | C0AP43 | Transcription elongation factor GreA | VVILNEATGK | *Borrelia sp.* | *Borrelia burgdorferi* |
| 108962 | BmR1_04g06010 | phosphoglycerate mutase | MISKLVLLR | *Babesia microti* | |
| 109366 | EOA79850.1 | hypothetical protein BBUCA8_03787 | VSLYSFMFVIADYLHSNYVVENFPQK | *Borrelia burgdorferi* | |
| 109366 | AFT83983 | chemotaxis histidine kinase | IMSLEDDPNNSDTIDEIFR | *Borrelia burgdorferi, Borrelia mayonii, Borrelia garinii, Borrelia bavariensis, Borrelia afzelii, Borrelia spielmanii* | |
| 643508 | WP_106017067 | immunogenic protein P37 | SNQDNQTLLLSLHQAIAK | *Borrelia burgdorferi* | |
| 643508 | YP_169211.1 | DNA-directed RNA polymerase subunit beta | DAAILSPCDGMVR | *Francisella tularensis, Francisella philomiragia, Francisella novicida, Francisella persica* | |
| 843502 | Q0SMZ6 | Borrelia Bdr family protein | NLEDRIFELNQK | *Borrelia afzelii* | |
| 843502 | Q50E31 | p-553 (Fragment) | DTINNNIQR | *Borrelia hermsii* | |
| 105158 | WP_011194102 | murein biosynthesis integral membrane protein MurJ | IMGFIKVK | *Borrelia bavariensis* | |
| 105158 | YP_169406.1 | hypothetical protein FTT_0359 | EIQLQQQQNAANQDK | *Francisella tularensis, Francisella hispaniensis* | |
| 108369 | H6VGK4 | Outer surface protein C | AILKTNGDKTLGAAELEK | *Borrelia afzelii, Borrelia burgdorferi* | |
| 108369 | A0A0E0SSC4 | Replicative DNA helicase | CATDKSTNLEEVFDVLQKK | *Borrelia burgdorferi* | |
| 108369 | A0A0E1U7Y5 | UDP-N-acetylmuramate--L-alanine ligase | NYEALEEAFLQYINNLK | *Borrelia burgdorferi* | |
| 435576 | WP_011181071.1 | membrane protein insertase YidC | LIQPMLLEIK | *Bartonella henselae* | |
| 435576 | WP_044104199.1 | GTPase Era | GADMVILVLDAKR | *Anaplasma phagocytophilum* | |
| 196390 | A0A0E1U8C0 | Acriflavine resistance protein | VTSNLDVEK | *Borrelia burgdorferi* | |
| 196390 | A0A0H3C216 | Flagellar hook capping protein | EFIAQMAQFSALEQMANMSR | *Borrelia burgdorferi* | |
| 196390 | C0AP43 | Transcription elongation factor GreA | VVILNEATGK | *Borrelia sp.* | *Borrelia burgdorferi* |
| 196390 | A0A1D8TEF9 | Lon protease | DDLPVIFLNK | *Borrelia miyamotoi* | |
| 196390 | A0A218PF23 | VlpB9 | LNELSSEISEAK | *Borrelia turicatae* | |
| 196390 | BMR1_03g04055 | tetratricopeptide repeat family protein | YILDPEDYDSGIYNLKQTLK | *Babesia microti* | |
| 196390 | BMR1_03g00540 | 1-deoxy-D-xylulose-5-phosphate synthase | QTSLPTGTKCQGTGLSLLIDR | *Babesia microti* | |
| 783249 | Q6ASF6 | Uncharacterized protein | ESELLMNLESNLK | *Borrelia bavariensis* | |
| 783249 | A0A1D8TE94 | CoA-disulfide reductase | TGLTEEAALK | *Borrelia miyamotoi* | |
| 783249 | W6TH26 | Uncharacterized protein | ELETINTIK | *Borrelia sp., Borrelia duttoni* | |
| 214378 | H6VGK4 | Outer surface protein C | AILKTNGDKTLGAAELEK | *Borrelia afzelii, Borrelia burgdorferi* | |
| 214378 | A0A0A7V1T0 | Pyruvate kinase | IENQEGIDNIEEIVK | *Borrelia burgdorferi, Borrelia garinii, Borrelia afzelii, Borrelia spielmanii, Borrelia bavariensis* | |
| 214378 | WP_011181038.1 | tRNA 2-thiouridine(34) synthase MnmA | AAETEMMLK | *Bartonella henselae* | |
| 729340 | I0FF14 | VmpS protein | DGVDSIIAEVK | *Borrelia burgdorferi, Borrelia garinii, Borrelia bavariensis, Borrelia mayonii* | |
| 108571 | WP_002661838 | Chemotaxis protein MotA (Motility protein A) | IDTEEAAVK | *Borrelia burgdorferi* | |
| 562445 | WP_117374824 | S41 family peptidase | EHVDEEALLEGALK | *Borrelia miyamotoi* | |
| 562445 | WP_006433391 | hypothetical protein | NLNTSEVILTQ | *Borrelia spielmanii* | |
| 562445 | WP_106017307 | V-type ATP synthase subunit E | NIGLHYDFSAETIADILFDY | *Borrelia burgdorferi* | |
| 562445 | XP_012648003 | mitogen-activated protein kinase | DMDQLNMIFNVLGTPW | *Babesia microti* | |
| 562445 | XP_021338751 | hypothetical protein BMR1_03g03870 | TSSFTGHLYNYMGFR | *Babesia microti* | |
| 837682 | C0ANF9 | Uncharacterized protein | FNNNYSNLNNNINAK | *Borrelia sp.* | *Borrelia burgdorferi* |
| 837682 | WP_012621115 | chromosome partitioning protein | IELNGNEIKKNR | *Borrelia garinii* | |
| 108294 | YP_169600.1 | hypothetical protein FTT_0576 | EYPIKIVESK | *Francisella hispaniensis* | |
| 108785 | A0A172XCQ6 | Phage portal protein | LSMSFNDLVILSDEQK | *Borrelia turicatae* | |
| 354291 | H6VGK4 | Outer surface protein C | AILKTNGDKTLGAAELEK | *Borrelia burgdorferi, Borrelia afzelii* | |
| 354291 | YP_169211.1 | DNA-directed RNA polymerase subunit beta | DAAILSPCDGMVR | *Francisella tularensis, Francisella philomiragia, Francisella novicida, Francisella persica* | |
| 908230 | G0ALZ3 | DNA ligase | VQQEIANLK | *Borrelia sp.* | *Borrelia spielmani* |
| 908230 | A0A0E1UAY2 | Magnesium transporter MgtE | IVFFVAPHHSDK | *Borrelia burgdorferi, Borrelia mayonii* | |
| 908230 | A0A0E1U8W9 | tRNA N6-adenosine threonylcarbamoyltransferase | HYDMGFPGGPNIEQISK | *Borrelia burgdorferi, Borrelia mayonii, Borrelia afzelii, Borrelia spielmanii, Borrelia garinii* | |
| 908230 | A0A0E1BWH2 | Chromosome partitioning protein parB | MVDINLLDVDK | *Borrelia hermsii* | |
| 908230 | A0A218PF75 | VlpD4 | SADSTTPADAATVGQQQ | *Borrelia turicatae* | |
| 908230 | BMR1_01G02630 | hypothetical protein | QISQFDISR | *Babesia microti* | |
| 108787 | BMR1_02g01770 | 26S proteasome non-ATPase regulatory subunit 10 | LLIDAGADVK | *Babesia microti* | |
| 108787 | Q5NG03 | Dual-specificity RNA methyltransferase RlmN | NLSAAEVIAQLWIAAR | *Francisella tularensis, Francisella hispaniensis, Francisella persica* | |
| 485701 | W5SD80 | Flagellar basal-body rod protein flgG | DSIAISEEGIISVK | *Borrelia miyamotoi* | |
| 485701 | BMR1_01G01220 | DNA topoisomerase II | SKIMDSVIAWAR | *Babesia microti* | |
| 485701 | WP_044104199.1 | GTPase Era | GADMVILVLDAKR | *Anaplasma phagocytophilum* | |
| 485701 | XP_012647760 | conserved Plasmodium protein, unknown function | HPYSNVSHYTNTSALMLQIK | *Babesia microti* | |
| 485701 | XP_021338380 | protein transport protein SEC24 | KPGPMGSGYGPPPK | *Babesia microti* | |
| 623456 | Q6UR22 | OspC (Fragment) | LKNSHAELGVAGNGATTDENAQK | *Borrelia afzelii* | |
| 623456 | XP_012649029 | hypothetical protein BMR1_03g02095 | YYIETMDFALGLLLPMACIMI | *Babesia microti* | |
| 623456 | WP_073999095 | DUF3996 domain-containing protein | FYDFNLLAIAALDFI | *Borrelia burgdorferi, Borrelia mayonii, Borrelia afzelii, Borrelia spielmanii* | |
| 623456 | XP_012647795 | Ribonuclease Z | QFNVNDILHFYDVPLSEN | *Babesia microti* | |
| 623456 | WP_006433624 | hypothetical protein | VDILISQLGISSDVQK | *Borrelia spielmanii* | |
| 1121119 | KJV66666 | prolipodiacylglyceryl transferase family protein | MSFHGGFIGSITCTFAVCK | *Anaplasma phagocytophilum* | |
| 1121119 | WP_075552323 | chemotaxis protein CheA | NNFCLSKSDLEEIR | *Borrelia mayonii* | |
| 1121119 | XP_012648074 | hypothetical protein BMR1_02g01520 | AASAAMQNSISLQQQK | *Babesia microti* | |
| 1121119 | WP_011181086 | transcriptional repressor | QMTQEIGFHANKSTIEV | *Bartonella henselae* | |
| 1121119 | WP_011179859 | leucine--tRNA ligase | TGHQGAALELSK | *Bartonella quintana* | |
| 739123 | A0A0E1UAQ1 | DNA-directed DNA polymerase | GAIDIVVFTESYER | *Borrelia burgdorferi, Borrelia garinii, Borrelia bavariensis, Borrelia afzelii* | |
| 739123 | A0A0E1U7Y5 | UDP-N-acetylmuramate--L-alanine ligase | NYEALEEAFLQYINNLK | *Borrelia burgdorferi* | |
| 108124 | I0FEL2 | Mlp lipoprotein family-containing protein | MIENEKKNITNYDK | *Borrelia sp.* | |
| 788653 | A1QZY3 | Chemotaxis protein CheY | INEVNEKLAR | *Borrelia turicatae* | |
| 108512 | WP_012421786 | UTP--glucose-1-phosphate uridylyltransferase | ILFNCATGLFNLEYLIENIGR | *Borrelia hermsii* | |
| 900019 | XP_021338125 | DNA-directed RNA polymerase III subunit C2 | LTNEILDQVIAG | *Babesia microti* | |
| 900019 | WP_091971958 | arginine--tRNA ligase | IESIMPEITQK | *Borrelia garinii, Borrelia bavariensis* | |
| 108838 | A0A0A7UVQ9 | ABC transporter substrate-binding protein | NAGNFEIIR | *Borrelia chilensis* | |
| 108838 | WP_012421970 | peptide chain release factor N(5)-glutamine methyltransferase | VTLSDISTK | *Borrelia hermsii, Borrelia garinii* | |
| 108838 | WP_002661838 | Chemotaxis protein MotA (Motility protein A) | IDTEEAAVK | *Borrelia burgdorferi* | |
| 990543 | WP_014695942 | signal peptidase I | QVNNSYYLNGNLMIGYK | *Borrelia sp.* | *Borrelia duttonii, Borrelia recurrentis* |
| 990543 | WP_088895116 | hypothetical protein | NEDLNEMKNIVK | *Borrelia turicatae* | |
| 178546 | WP_028328185 | Variable large protein 15/16 | TLNTLIIAIR | *Borrelia turicatae* | |
| 100981 | WP_106405031 | nicotinate (nicotinamide) nucleotide adenylyltransferase | NDKLFLIIGDDLFQNFDSWK | *Borrelia burgdorferi* | *Borrelia mayonii, Borrelia garinii, Borrelia bavariensis, Borrelia spielmanii, Borrelia afzelii* |
| 109126 | ACL35186.1 | antigen, S2 | GNSIEELKGIK | *Borrelia garinii* | |
| 109126 | AAL99373.1 | flagellin, partial | DAIMTDEIVASTTNSILTQSAMAMIAQANQV | *Borrelia burgdorferi, Borrelia miyamotoi* | *Borrelia turicatae* |
| 109126 | WP_106022635 | response regulator | FQSVNGDNK | *Borrelia burgdorferi* | |
| 671129 | WP_012622036 | outer surface protein C (OspC) | DGGQNGSLLAGAYAISTVIIEK | *Borrelia burgdorferi* | |
| 671129 | AHH03598 | P66 protein precursor | GIGLAWNTDEGEK | *Borrelia hermsii* | |
| 671129 | ANC34866 | hypothetical protein P029_04030 | LGNASVEIVNESDNHIGHLGSAGY | *Anaplasma phagocytophilum* | |
| 810141 | C0AP43 | Transcription elongation factor GreA | VVILNEATGK | *Borrelia sp.* | *Borrelia burgdorferi* |
| 810141 | BMR1_03g00675 | hypothetical protein | QAVLMGLLK | *Babesia microti* | |
| 108319 | O50616 | Outer surface protein A (Fragment) | GTSDKSNGSGILEGEK | *Borrelia garinii* | |
| 108319 | W5SEH9 | Endonuclease MutS2 | QNSIISVEEIK | *Borrelia miyamotoi* | |
| 108319 | I0FEW4 | Variable large protein | VFLSDMVNLGK | *Borrelia sp.* | *Borrelia garinii* |
| 108319 | W5SLA2 | Variable large protein VlpA21H | VLAAGANAGAAVGEK | *Borrelia sp., Borrelia duttoni* | |
| 108853 | H6VGK4 | Outer surface protein C | AILKTNGDKTLGAAELEK | *Borrelia afzelii, Borrelia burgdorferi* | |
| 964452 | XP_012648916 | hypothetical protein BMR1_03g01530 | VSFGSIGGIGK | *Babesia microti* | |
| 964452 | XP_012650249 | guanine nucleotide | QLYDEEINGK | *Babesia microti* | |
| 964452 | XP_012650305 | hypothetical protein BmR1_04g08600 | LVGLDLIGAR | *Babesia microti* | |
| 964452 | XP_012649063 | aarF domain | DQLNLANISNNVMSR | *Babesia microti* | |
| 964452 | XP_021338538 | lysophospholipase | DENLPIPPNIFPFLNFR | *Babesia microti* | |
| 243325 | CAG47088 | outer surface protein C | DLGTAGGNATDDHAK | *Borrelia spielmanii* | |
| 243325 | WP_025407384 | HAD family hydrolase | LYPEININLIMFPEFLK | *Borrelia turicatae* | |
| 243325 | WP_002656071 | PQQ-like beta-propeller repeat protein | ILIPLANEK | *Borrelia burgdorferi* | |
| 243325 | XP_012649462 | hypothetical protein BMR1_03g04255 | SPGNPPPINNSAINTVLTSLNK | *Babesia microti* | |
| 980000 | XP_012650119 | protein phosphatase 3, catalytic subunit | GLENALENFNAAK | *Babesia microti* | |
| 980000 | WP_042995410 | protease modulator HflC | FLFMSSTIVFVLMVFW | *Bartonella quintana* | |
| 980000 | AGR80811 | hypothetical protein WSQ_04230 | SAPAIPVMMLLSNDLAAR | *Anaplasma phagocytophilum* | |
| 980000 | WP_119024075 | hypothetical protein | NEIQTEIVKINNEIK | *Borrelia turicatae* | |
| 980000 | WP_011178961 | Vomp family autotransporter | AFGGVDSSLSNLNDR | *Bartonella quintana* | |
| 980000 | EOA62502 | HlyD family type I secretion membrane fusion protein | MEVVPIDDDFIIDAK | *Anaplasma phagocytophilum* | |
| 980000 | XP_021338832 | rhoptry neck protein 2 | NLESAFLDLLQDLVIMVTNPGK | *Babesia microti* | |
| 980000 | XP_012649420 | hypothetical protein BMR1_03g04045 | MDGPKTNEIVTAALENLLLQLT | *Babesia microti* | |
| 980000 | XP_021338135 | conserved Plasmodium protein, unknown function | NDIYDIGDAFFSDISEK | *Babesia microti* | |
| 980000 | XP_012649985 | Germinal-center associated nuclear protein | SPVIVNTNIPAN | *Babesia microti* | |

**Table S5.** List of acute LB and non acute tick-borne disease patients and results of the mass spectrometry analysis. Sample ID = patient identification number, *Borrelia* = amino acid sequence of peptides unambiguously attributed to at least one pathogenic species of *Borrelia*, *Bartonella* = amino acid sequence of peptides unambiguously attributed to at least one pathogenic species of *Bartonella*, *Rickettsia* = amino acid sequence of peptides unambiguously attributed to at least one pathogenic species of *Rickettsia*, *Babesia* = amino acid sequence of peptides unambiguously attributed to at least one pathogenic species of *Babesia*, *Anaplasma* = amino acid sequence of peptides unambiguously attributed to at least one pathogenic species of *Anaplasma*, *Francisella* = amino acid sequence of peptides unambiguously attributed to *Francisella sp*, *Ehrlichia* = amino acid sequence of peptides unambiguously attributed to at least one pathogenic species of *Ehrlichia.* Blue font indicates findings in an acute LB patient. Black font indicates findings in a non acute patient with clinical suspicion of tick-borne diseases.

| **Sample_ID** | **Anaplasma** | **Babesia** | **Bartonella** | **Borrelia** | **Ehrlichia** | **Francisella** | **Rickettsia** |
| --- | --- | --- | --- | --- | --- | --- | --- |
| **100981** | **-** | **-** | **-** | **NDKLFLIIGDDLFQNFDSWK (WP_106405031)** | **-** | **-** | **-** |
| **102346** | **-** | **-** | **-** | **-** | **-** | **-** | **-** |
| **102424** | **-** | **-** | **-** | **-** | **-** | **-** | **-** |
| **104821** | **-** | **-** | **-** | **AILKTNGDKTLGAAELEK (H6VGK4) - NNFCLSKSDLEEIR (WP_075552323)** | **-** | **-** | **-** |
| **105158** | **-** | **-** | **-** | **ImGFIKVK (WP_011194102)** | **-** | **EIQLQQQQNAANQDK (YP_169406.1)** | **-** |
| **108061** | **-** | **-** | **-** | **KTKQSDENISVENKPK (WP_031541693) - ELETINTIK (W6TH26)** | **-** | **-** | **-** |
| **108124** | **-** | **-** | **-** | **mIENEKKNITNYDK (I0FEL2)** | **-** | **-** | **-** |
| **108294** | **-** | **-** | **-** | **-** | **-** | **EYPIKIVESK (YP_169600.1)** | **-** |
| **108319** | **-** | **-** | **-** | **GTSDKSNGSGILEGEK (O50616) - QNSIISVEEIK (W5SEH9) - VFLSDMVNLGK (I0FEW4) - VLAAGANAGAAVGEK (W5SLA2)** | **-** | **-** | **-** |
| **108369** | **-** | **-** | **-** | **AILKTNGDKTLGAAELEK (H6VGK4) - cATDKSTNLEEVFDVLQKK (A0A0E0SSC4) - NYEALEEAFLQYINNLK (A0A0E1U7Y5)** | **-** | **-** | **-** |
| **108512** | **-** | **-** | **-** | **ILFNcATGLFNLEYLIENIGR (WP_012421786)** | **-** | **-** | **-** |
| **108571** | **-** | **-** | **-** | **IDTEEAAVK (WP_002661838)** | **-** | **-** | **-** |
| **108785** | **-** | **-** | **-** | **LSmSFNDLVILSDEQK (A0A172XCQ6)** | **-** | **-** | **-** |
| **108786** | **-** | **-** | **-** | **-** | **-** | **-** | **-** |
| **108787** | **-** | **LLIDAGADVK (BMR1_02g01770)** | **-** | **-** | **-** | **NLSAAEVIAQLWIAAR (Q5NG03)** | **-** |
| **108838** | **-** | **-** | **-** | **NAGNFEIIR (A0A0A7UVQ9) - VTLSDISTK (WP_012421970) - IDTEEAAVK (WP_002661838)** | **-** | **-** | **-** |
| **108853** | **-** | **-** | **-** | **AILKTNGDKTLGAAELEK (H6VGK4)** | **-** | **-** | **-** |
| **108962** | **-** | **mISKLVLLR (BmR1_04g06010)** | **-** | **VVILNEATGK (C0AP43)** | **-** | **-** | **-** |
| **109126** | **-** | **-** | **-** | **GNSIEELKGIK (ACL35186.1) - DAIMTDEIVASTTNSILTQSAMAMIAQANQV (AAL99373.1) - FQSVNGDNK (WP_106022635)** | **-** | **-** | **-** |
| **109366** | **-** | **-** | **-** | **VSLYSFMFVIADYLHSNYVVENFPQK (EOA79850.1) - IMSLEDDPNNSDTIDEIFR (AFT83983)** | **-** | **-** | **-** |
| **111345** | **-** | **-** | **-** | **DLVLLDVGLPDINGYEVCR (WP_006433838) - PEMGINLVMFPEFLK (WP_025406568)** | **-** | **-** | **-** |
| **1121119** | **MSFHGGFIGSITCTFAVCK (KJV66666)** | **AASAAMQNSISLQQQK (XP_012648074)** | **QMTQEIGFHANKSTIEV (WP_011181086) - TGHQGAALELSK (WP_011179859)** | **NNFCLSKSDLEEIR (WP_075552323)** | **-** | **-** | **-** |
| **114343** | **-** | **-** | **-** | **DQVNQALDTINKVAEDVSNK (A0A1L6VVD6) - LKNSHAELGVAGNGATTDENAQK (Q6UR22)** | **-** | **-** | **-** |
| **123945** | **-** | **-** | **-** | **IDMLNTNTIDSNLDNIFNIK (Q0SN88) - KDEAVQmIIKAADVDK (A0A1D8TDG3) - LGSLSGSEVK (W6THA6) - GLETTDNISDKLKR (B5RP22) - DILSEELGIQK (EED30070) - EIEENLSLIGK (WP_044051985)** | **-** | **-** | **-** |
| **152946** | **-** | **-** | **-** | **KDNSKLVEAER (WP_071983715)**  **ITAATGCGIPISK (WP_028328339)**  **KIENKVGSNAAGT (WP_012622204)**  **DKILKNFVFK (WP_029346894)**  **VGTLIYGIFLR (WP_014696474)** | **-** | **-** | **IGRSSLEQAAEK (ABV75938)** |
| **158492** | **-** | **IAQELSLHDLDTANAKPMSGNDVVSILGQYL (XP_021337180)** | **-** | **-** | **-** | **-** | **-** |
| **163525** | **-** | **-** | **-** | **-** | **-** | **-** | **-** |
| **170103** | **-** | **-** | **-** | **-** | **-** | **-** | **-** |
| **178546** | **-** | **-** | **-** | **TLNTLIIAIR (WP_028328185)** | **-** | **-** | **-** |
| **193471** | **-** | **-** | **-** | **ILGYAIEGK (A0A0A7UWS0)** | **-** | **-** | **-** |
| **196390** | **-** | **YILDPEDYDSGIYNLKQTLK (BMR1_03g04055) - QTSLPTGTKcQGTGLSLLIDR (BMR1_03g00540)** | **-** | **VTSNLDVEK (A0A0E1U8C0) - EFIAQMAQFSALEQmANmSR (A0A0H3C216) - VVILNEATGK (C0AP43) - DDLPVIFLNK (A0A1D8TEF9) - LNELSSEISEAK (A0A218PF23)** | **-** | **-** | **-** |
| **198887** | **-** | **-** | **-** | **-** | **-** | **-** | **-** |
| **213567** | **-** | **-** | **-** | **LATVNEAEGK (A0A1L8Z945) - NDVSEEKPEIK (WP_014696160)** | **-** | **-** | **-** |
| **214378** | **-** | **-** | **AAETEMmLK (WP_011181038.1)** | **AILKTNGDKTLGAAELEK (H6VGK4) - IENQEGIDNIEEIVK (A0A0A7V1T0)** | **-** | **-** | **-** |
| **221347** | **-** | **-** | **-** | **-** | **-** | **-** | **-** |
| **226622** | **-** | **-** | **-** | **-** | **-** | **-** | **-** |
| **228321** | **-** | **-** | **-** | **EILENDADVNVK (A0A0A7UYW5)** | **-** | **-** | **-** |
| **229879** | **-** | **-** | **-** | **-** | **-** | **-** | **-** |
| **238401** | **-** | **-** | **-** | **-** | **-** | **-** | **-** |
| **242666** | **-** | **-** | **-** | **LKNSHAELGVAGNGATTDENAQK (Q6UR22)** | **-** | **-** | **-** |
| **243325** | **-** | **SPGNPPPINNSAINTVLTSLNK (XP_012649462)** | **-** | **DLGTAGGNATDDHAK (CAG47088) - LYPEININLIMFPEFLK (WP_025407384) - ILIPLANEK (WP_002656071)** | **-** | **-** | **-** |
| **245023** | **-** | **-** | **-** | **VYLNNISDLK (B7XS60)** | **MGVFLLMLSLLMCVR (WP_044170479) - QNITSGSLLDVASGTGDIAIK (WP_024072397)** | **-** | **-** |
| **246483** | **-** | **-** | **-** | **-** | **-** | **-** | **-** |
| **265499** | **-** | **-** | **-** | **NIEEELEKLAEPQNIEDK (G0ANW1) - LATVNEAEGK (A0A1L8Z945)** | **-** | **-** | **-** |
| **269504** | **-** | **-** | **-** | **-** | **-** | **-** | **-** |
| **280133** | **LVMQLDSGAKLVFHMGMSGR (WP_070258649)** | **-** | **-** | **-** | **-** | **-** | **-** |
| **285020** | **-** | **MHISQMTNSILLSFTYSY (XP_012649696)** | **-** | **LSESIETALSV (WP_025434152) - GNWGGALIQR (WP_064536561)** | **-** | **-** | **-** |
| **310741** | **-** | **-** | **-** | **VNESDLGIKALDEK (Q6ASN6) – FNVEACFPTLIVEK (Q0SN56)** | **-** | **-** | **-** |
| **310742** | **-** | **-** | **-** | **FNVEAcFPTLIVEK (Q0SN56)** | **-** | **-** | **-** |
| **315854** | **-** | **VLNCDIKELGK (XP_012649006) - LLVTLDVCNEL (XP_012650394) - QAVNDSVKNSVK (XP_012647924)** | **-** | **KTKQSDENISVENKPK (WP_031541693) - DTAANKTEAEAAKGVAANAVNK (W5STS8) - KDEAVQmIIKAADVDK (A0A1D8TDG3) - mLELTQDK (WP_040236434.1) - NETNNLHEEIIDEKAL (WP_045245604) - KEQKGGTNGEIK (WP_117374741) - GGRYLGRNNGPK (WP_119024258) - FNGGGILKLSDPA (WP_025407406) - KVPLDLAMTGEV (WP_031541621) - KNDGSGGKLEGVK (BAA22351)** | **-** | **-** | **QQAIDQSKKVGS (WP_012737743)** |
| **321456** | **-** | **-** | **-** | **-** | **-** | **-** | **-** |
| **324311** | **-** | **-** | **-** | **IVGEGNGSVEL (Q661Y3) - KDEAVQmIIKAADVDK (A0A1D8TDG3) - IYDPPFAIYYK (A0A1D8TDQ0)** | **-** | **-** | **-** |
| **324344** | **-** | **-** | **-** | **-** | **-** | **-** | **-** |
| **343143** | **-** | **-** | **-** | **-** | **-** | **-** | **-** |
| **343222** | **-** | **-** | **-** | **-** | **-** | **-** | **-** |
| **348119** | **-** | **YILDPEDYDSGIYNLKQTLK (BMR1_03g04055)** | **-** | **VVILNEATGK (C0AP43) - TLNTLIIAIR (WP_028328185)** | **-** | **-** | **EENGNIImQGAATLVAR (H6PTL6)** |
| **3504713** | **-** | **-** | **-** | **-** | **-** | **-** | **-** |
| **354291** | **-** | **-** | **-** | **AILKTNGDKTLGAAELEK (H6VGK4)** | **-** | **DAAILSPcDGMVR (YP_169211.1)** | **-** |
| **362346** | **-** | **-** | **-** | **KDEAVQmIIKAADVDK (A0A1D8TDG3)** | **-** | **-** | **-** |
| **365481** | **-** | **-** | **-** | **cATDKSTNLEEVFDVLQKK (A0A0E0SSC4) - LKNSHAELGVAGNGATTDENAQK (Q6UR22) - LKDNHAQLGIQNGASLDDEAK (Q49579)** | **-** | **-** | **-** |
| **413743** | **-** | **VDLNGEHSLDK (XP_021337885) - IGFNVANSLAITCTNITINFN (XP_012650305) - VAVLEAANLGLLNLNSPVSNYWPMFGHK (XP_012649063)** | **-** | **FGGLHTIVDDATILEFQYK (WP_014023195) - VFINDLLEPSLK (WP_031558227) - YALQKLMAENK (WP_020954596)** | **-** | **-** | **-** |
| **422311** | **-** | **-** | **-** | **-** | **-** | **-** | **-** |
| **432450** | **-** | **-** | **-** | **-** | **-** | **-** | **-** |
| **435576** | **GADmVILVLDAKR (WP_044104199.1)** | **-** | **LIQPMLLEIK (WP_011181071.1)** | **-** | **-** | **-** | **-** |
| **436842** | **-** | **-** | **-** | **NEIFIPTR (A0A0E1C0C2)** | **-** | **-** | **-** |
| **441278** | **-** | **-** | **-** | **mPLFPIDK (A0A0E1C250) - FDLVLYQYHLVLK (WP_106019189) - IPQNINNNFYFLINNQK (WP_110503430) - FFPSNIAFANSVSVPM (WP_146124657)** | **-** | **-** | **-** |
| **444356** | **-** | **-** | **-** | **-** | **GNTDEVVYQIVGEYEADISK (WP_045804957)** | **-** | **-** |
| **453653** | **-** | **-** | **-** | **-** | **-** | **-** | **-** |
| **453742** | **-** | **-** | **-** | **VVILNEATGK (WP_008882341) - LATVNEAEGK (A0A1L8Z945) - FVYIGNVDNMGYTINFK (WP_029346900)** | **-** | **-** | **-** |
| **459235** | **-** | **-** | **-** | **SAKEVINNTSTMEK (WP_008882425) – SSSVDGFVSFKEYKER (WP_015026969)** | **-** | **-** | **-** |
| **460722** | **-** | **-** | **-** | **DGPVSLAISR (WP_008882409) - TEDDHLIEMINEPENFNK (WP_014023462) - KDEAVQMIIKAADVDK (WP_117374895)** | **-** | **-** | **-** |
| **463256** | **-** | **-** | **-** | **NLSLFTDFYEISmmNAYFIK (A0A0A7UVW0) – QKATGAINAVSGEQIL (WP_145940815.1)** | **-** | **-** | **-** |
| **468087** | **-** | **-** | **-** | **-** | **-** | **-** | **-** |
| **485701** | **GADmVILVLDAKR (WP_044104199.1)** | **SKIMDSVIAWAR (BMR1_01G01220) - HPYSNVSHYTNTSALMLQIK (XP_012647760) - KPGPMGSGYGPPPK (XP_021338380)** | **-** | **DSIAISEEGIISVK (W5SD80)** | **-** | **-** | **-** |
| **522431** | **-** | **-** | **-** | **AILKTNGDKTLGAAELEK (H6VGK4)** | **-** | **-** | **-** |
| **531792** | **-** | **-** | **-** | **cATDKSTNLEEVFDVLQKK (A0A0E0SSC4)** | **-** | **-** | **-** |
| **534390** | **-** | **-** | **-** | **IVATSLNNQSVSLEDFPIDNK (W5SJU8) - NNLmPVIK (A1R0L1)** | **-** | **-** | **-** |
| **542019** | **-** | **QSLIIGALAR (XP_021338817) - ITNDYVHKR (XP_012647552)** | **-** | **ESLDLIINR (WP_106019052) - VDVVNTELVDDDLLK (WP_014023373)** | **-** | **-** | **-** |
| **555444** | **-** | **-** | **-** | **LLESGSTIVGMQNILEL (WP_014023569)** | **-** | **-** | **-** |
| **556555** | **-** | **-** | **NGFLGLIFAHLMSTHN (WP_011181350)** | **-** | **-** | **-** | **-** |
| **562445** | **-** | **DMDQLNMIFNVLGTPW (XP_012648003) - TSSFTGHLYNYMGFR (XP_021338751)** | **-** | **EHVDEEALLEGALK (WP_117374824) - NLNTSEVILTQ (WP_006433391) - NIGLHYDFSAETIADILFDY (WP_106017307)** | **-** | **-** | **-** |
| **562845** | **-** | **-** | **-** | **ILGYAIEGK (A0A0A7UWS0)** | **-** | **-** | **-** |
| **599861** | **-** | **QDKQALSDQKK (XP_021337329) - SLPMLSGIIGER (XP_021338144)** | **-** | **ELGELAYKTHK (WP_010889724) - VKANTTAAKGGEAA (WP_141631485) - EGGLVTRVSENK (WP_156110316) - KVDIIKCAGGDL (WP_071983718)** | **RVPIIAGSSSNST (WP_006009763)** | **-** | **MLNATNAILITGG (WP_012149474)** |
| **611349** | **-** | **LSEEVTEFDNAIAQDK (BMR1_03g04565)** | **-** | **ELELIENEIIK (Q660X6) - FEEIKIEAR (T1ECH2) - YMLDNENASIFDLLK (WP_106011477) - NYVHNVEIYNTYSMLK (WP_010883900)** | **-** | **YLVLGLFMFLYGcAR (YP_170186.1)** | **-** |
| **623456** | **-** | **YYIETMDFALGLLLPMACIMI (XP_012649029) - QFNVNDILHFYDVPLSEN (XP_012647795)** | **-** | **LKNSHAELGVAGNGATTDENAQK (AAR18256) - FYDFNLLAIAALDFI (WP_073999095) - VDILISQLGISSDVQK (WP_006433624)** | **-** | **-** | **-** |
| **634958** | **-** | **-** | **-** | **GTSDKSNGSGILEGEK (O50616)** | **-** | **-** | **-** |
| **639032** | **-** | **VSMESADNITTI (XP_012647349)** | **-** | **IPNTLEEIDQYL (WP_117375036) - EIANVQMQLENIISK (WP_106022834)** | **SSLLADDSGGNIAR (WP_044160877)** | **-** | **-** |
| **643508** | **-** | **-** | **-** | **SNQDNQTLLLSLHQAIAK (WP_106017067)** | **-** | **DAAILSPcDGMVR (YP_169211.1)** | **-** |
| **645608** | **-** | **-** | **-** | **HFLSIFEER (WP_008882360)** | **-** | **-** | **-** |
| **648842** | **-** | **-** | **-** | **-** | **-** | **-** | **-** |
| **662221** | **-** | **-** | **-** | **-** | **-** | **-** | **-** |
| **667829** | **-** | **-** | **-** | **DFVFAINNLIFTLNNL (WP_106011467)** | **IGLDNATLTFENSMK (WP_044147846)** | **-** | **-** |
| **671129** | **LGNASVEIVNESDNHIGHLGSAGY (ANC34866)** | **-** | **-** | **DGGQNGSLLAGAYAISTVIIEK (WP_012622036) - GIGLAWNTDEGEK (AHH03598)** | **-** | **-** | **-** |
| **671900** | **-** | **QPINSYISDDAESQINNASNMMYK (XP_012649780)** | **-** | **-** | **-** | **-** | **-** |
| **676765** | **-** | **SLDNSNDSTFVIQDVANMIK (XP_012648506)** | **-** | **-** | **-** | **-** | **-** |
| **682141** | **-** | **-** | **-** | **IREQTQEEIQNIK (A0A218PF40) - IAMPIFISSMTGGVK (WP_081719344) - YTNALNNFFTSL (WP_006433983)** | **-** | **-** | **QFMPAGSIMVTGSHN (WP_041078759)** |
| **687321** | **-** | **-** | **-** | **-** | **-** | **-** | **-** |
| **711111** | **-** | **-** | **VQETLCGQLNLCWPD (WP_011181378)** | **PVATRIEMIDFLSSTLDIDK (AFU75021)** | **-** | **-** | **-** |
| **711123** | **-** | **HFGEPETYISALALK (XP_021337280) - ILELESQNLE (XP_012647489)** | **-** | **-** | **-** | **-** | **-** |
| **723941** | **-** | **-** | **-** | **LKNSHAELGVAGNGATTDENAQK (Q6UR22)** | **-** | **-** | **-** |
| **729340** | **-** | **-** | **-** | **FEDAIVLRDK (WP_038377001)** | **-** | **-** | **-** |
| **732491** | **-** | **-** | **-** | **VNESDLGIKALDEK (Q6ASN6) - RNSNSTIIVNNK (A0A0E1U9X9)** | **-** | **-** | **-** |
| **732902** | **FSHEAVVTR (WP_011450270.1)** | **-** | **-** | **HHNAYTVNLNSVLEK (A1QYV0)** | **-** | **-** | **IGEMITADmLNVINDLK (H6PWA1)** |
| **739123** | **-** | **-** | **-** | **GAIDIVVFTESYER (A0A0E1UAQ1) - NYEALEEAFLQYINNLK (A0A0E1U7Y5)** | **-** | **-** | **-** |
| **742665** | **-** | **-** | **-** | **-** | **-** | **-** | **YVILINGGDIK (WP_085065713)** |
| **748990** | **-** | **FSSEPDDLTEK (BmR1_04g06597)** | **MTQLQETADGIEAIAVcKIK (WP_034447395.1)** | **-** | **-** | **-** | **IGEMITADmLNVINDLK (H6PWA1)** |
| **753564** | **-** | **-** | **-** | **-** | **-** | **-** | **-** |
| **756890** | **-** | **-** | **-** | **LKNSHAELGVAGNGATTDENAQK (Q6UR22) - EDANLIDLQLNVNK (W5SCW8) - FHSNALEVQVQAEIEAR (A0A0R9QEI9)** | **-** | **-** | **-** |
| **771232** | **-** | **-** | **SFPIFTSNNQTSTAIIPSD (WP_011181309)** | **LLMADISSLEEAINADR (WP_117375034)** | **-** | **-** | **-** |
| **775488** | **-** | **-** | **-** | **-** | **-** | **-** | **-** |
| **781022** | **-** | **-** | **LPKNLSAVANPK (WP_011179070) - IIGTPPRNLGTI (WP_111738306)** | **KIIIKFPEYT (WP_044052160) - QISILHNSLVK (AJA90394) - KKENLANPIPK (WP_038363604) - LNAGIVGLPNVGK (WP_031489998) - QVKIPLNVNLN (WP_025408373) - NISKQNPIKPL (WP_075552597)** | **-** | **-** | **-** |
| **783249** | **-** | **-** | **-** | **ESELLMNLESNLK (Q6ASF6) - TGLTEEAALK (A0A1D8TE94) - ELETINTIK (W6TH26)** | **-** | **-** | **-** |
| **788653** | **-** | **-** | **-** | **INEVNEKLAR (A1QZY3)** | **-** | **-** | **-** |
| **790907** | **-** | **-** | **-** | **SNQDNQTLLLSLHQAIAK (WP_106017067) - LKNSHAELGVAGNGATTDENAQK (Q6UR22)** | **-** | **-** | **-** |
| **810141** | **-** | **QAVLMGLLK (BMR1_03g00675)** | **-** | **VVILNEATGK (C0AP43)** | **-** | **-** | **-** |
| **812785** | **-** | **LVHLILFTQPA (XP_012647923)** | **QGIVFLKKATF (WP_111738296)** | **QNGEQSKEVIK (W5SQT1) - YKKIPTFNLK (WP_071983560) - KIDFNKVFIK (EEH00288) - VKAAKGAGVPEPK (ANA43971) - LTFIARLGTFL (AHH12137) - GIVSILVFEFK (AJA67228)** | **AALFIGYAVILT (AHX09459)** | **-** | **-** |
| **816529** | **-** | **-** | **-** | **-** | **-** | **-** | **-** |
| **820444** | **-** | **-** | **-** | **-** | **-** | **-** | **-** |
| **822221** | **-** | **-** | **GVGLYTIEFEK (WP_011180023)** | **FNNLNNDLGEFEFSR (WP_025443599) - IENLNEATLK (WP_020732484) - DFFKVILGIIK (WP_106022675)** | **-** | **-** | **-** |
| **823161** | **-** | **-** | **-** | **-** | **-** | **-** | **-** |
| **823733** | **SVVNLPAIDMLT (WP_080947527)** | **QIVAQLCDTLK (XP_021337167) - GDVSVSRAQLEK (XP_012648084) - LDGLVLMNREI (XP_021337985)** | **-** | **LNELSSEISEAK (A0A218PF23) - YIGSYLAILEF (AGJ81566) - EGLTIGNAKLCL (WP_088895085) - EGMLILQENIL (WP_002658307)** | **-** | **-** | **-** |
| **829147** | **-** | **-** | **-** | **-** | **-** | **-** | **-** |
| **832374** | **-** | **-** | **-** | **LKNSHAELGVAGNGATTDENAQK (Q6UR22)** | **-** | **-** | **-** |
| **837682** | **-** | **-** | **-** | **FNNNYSNLNNNINAK (C0ANF9) - IELNGNEIKKNR (WP_012621115)** | **-** | **-** | **-** |
| **843502** | **-** | **-** | **-** | **NLEDRIFELNQK (Q0SMZ6) - DTINNNIQR (Q50E31)** | **-** | **-** | **-** |
| **849011** | **-** | **-** | **-** | **-** | **-** | **-** | **-** |
| **865111** | **-** | **-** | **-** | **-** | **-** | **-** | **-** |
| **871679** | **-** | **-** | **-** | **-** | **-** | **-** | **-** |
| **881776** | **-** | **EAPEIIFNPHLNNK (XP_012647479)** | **-** | **GAQQEGAQQPAPA (BAA82630) - NISKSGEITVALDDTASAN (BAA19222) - LKDNHAQLGIQNGASLDDEAK (Q49579)** | **-** | **-** | **-** |
| **882710** | **-** | **-** | **-** | **-** | **-** | **-** | **-** |
| **886279** | **-** | **-** | **-** | **ELATEDEINNATFQLREL (WP_020732373) - NDTPISPEQNTRKATAN (WP_144033213)** | **-** | **-** | **-** |
| **887691** | **-** | **-** | **-** | **LATVNEAEGK (A0A1L8Z945)** | **-** | **-** | **EYNYNMPFMLNK (WP_012150208)** |
| **887699** | **-** | **-** | **-** | **-** | **-** | **-** | **-** |
| **889597** | **-** | **-** | **-** | **LATVNEAEGK (A0A1L8Z945) - GGYLDHVNHAGAKKVILTVPAK (3HJA_A)** | **-** | **-** | **-** |
| **889992** | **-** | **-** | **-** | **-** | **DLQDVASHESGVSDQPA (WP_143485395)** | **-** | **-** |
| **890217** | **-** | **GLGNIIFENTR (BMR1_02g00565) - DFIVNIEDINPSVYSDSIK (XP_012649545)** | **mIQETTPSPFIR (WP_011181139.1) - LIQPMLLEIK (WP_011181071.1)** | **SLENYKPLR (WP_151060615)** | **-** | **-** | **-** |
| **891284** | **-** | **TEKPSIVTLTLDLMEAYK (XP_012649113) - SLFYIATSGNISVEESNLYEAK (XP_012647719)** | **-** | **-** | **-** | **-** | **-** |
| **893649** | **-** | **-** | **-** | **FMIGSNDINVIGIDK (WP_108728866)** | **-** | **-** | **-** |
| **899012** | **-** | **-** | **-** | **-** | **-** | **-** | **-** |
| **900019** | **-** | **LTNEILDQVIAG (XP_021338125)** | **-** | **IESIMPEITQK (WP_091971958)** | **-** | **-** | **-** |
| **906453** | **-** | **NIDESNIEHLK (BMR1_03g00020)** | **-** | **FEEIKIEAR (T1ECH2) - YKMYDIEDSLEMYSK (A0A1D8TDR2)** | **-** | **-** | **-** |
| **906534** | **-** | **-** | **-** | **LELYIENNVEPIK (B7XS64)** | **-** | **-** | **-** |
| **908230** | **-** | **QISQFDISR (BMR1_01G02630)** | **-** | **VQQEIANLK (G0ALZ3) - IVFFVAPHHSDK (A0A0E1UAY2) - HYDMGFPGGPNIEQISK (A0A0E1U8W9) - MVDINLLDVDK (A0A0E1BWH2) - SADSTTPADAATVGQQQ (A0A218PF75)** | **-** | **-** | **-** |
| **911453** | **-** | **-** | **-** | **-** | **-** | **-** | **-** |
| **917548** | **-** | **-** | **-** | **-** | **-** | **-** | **-** |
| **923481** | **-** | **GLGNIIFENTR (BMR1_02g00565) - LSEEVTEFDNAIAQDK (BMR1_03g04565) - DEQSQASLGDIVR (XP_012649617)** | **-** | **-** | **-** | **-** | **IGEMITADmLNVINDLK (H6PWA1)** |
| **923856** | **-** | **MNLGDTLQIGSLNFAETHF (XP_021337827) - YNNNYIINK (YP_009363174)** | **-** | **-** | **HHAVPMGFGVITAGSLEIAMAR (WP_011452810)** | **-** | **-** |
| **957477** | **-** | **-** | **-** | **-** | **ISGTTGIILDDVLAG (WP_011452888) - QPVFVSESTVNDTISILR (WP_044170504) - YPDTNIIIVGHTDTR (WP_006011115)** | **-** | **-** |
| **964233** | **-** | **-** | **-** | **QVNNSYYLNGNLmIGYK (WP_014695942)** | **-** | **-** | **-** |
| **964452** | **-** | **VSFGSIGGIGK (XP_012648916) - QLYDEEINGK (XP_012650249) - LVGLDLIGAR (XP_012650305) - DQLNLANISNNVMSR (XP_012649063) - DENLPIPPNIFPFLNFR (XP_021338538)** | **-** | **-** | **-** | **-** | **-** |
| **974321** | **-** | **-** | **-** | **-** | **-** | **-** | **-** |
| **980000** | **SAPAIPVMMLLSNDLAAR (AGR80811) - MEVVPIDDDFIIDAK (EOA62502)** | **GLENALENFNAAK (XP_012650119) - NLESAFLDLLQDLVIMVTNPGK (XP_021338832) - MDGPKTNEIVTAALENLLLQLT (XP_012649420) - NDIYDIGDAFFSDISEK (XP_021338135) - SPVIVNTNIPAN (XP_012649985)** | **FLFMSSTIVFVLMVFW (WP_042995410) - AFGGVDSSLSNLNDR (WP_011178961)** | **NEIQTEIVKINNEIK (WP_119024075)** | **-** | **-** | **-** |
| **985819** | **-** | **-** | **-** | **EIDNYLLKEELQ (WP_106017219) - MNGVENVFNLNIS (WP_008882406) - NYDSFTSSIR (AAX17020)** | **-** | **-** | **-** |
| **987783** | **-** | **-** | **-** | **-** | **-** | **-** | **-** |
| **990543** | **-** | **-** | **-** | **QVNNSYYLNGNLmIGYK (WP_014695942) - NEDLNEMKNIVK (WP_088895116)** | **-** | **-** | **-** |
| **991873** | **-** | **SITHSNGYCKPCVFANK (XP_028871981)** | **-** | **-** | **-** | **SYMQRYAYRITQEIIK (WP_003020451)** | **-** |
| **994721** | **-** | **LMPCLVGLLLCILPGLEDDK (XP_021338357) - RPEIDNIFSICLSD (XP_021337625) - ILVVAADDGIMPQTVESYK (XP_012648602)** | **-** | **SAILKISAYDQGNVNFAYYK (WP_025407311)** | **-** | **-** | **-** |
| **998999** | **-** | **QTTSTSDTVSHSPTVVTK (XP_021337692)** | **-** | **NPYVYLEDFNK (WP_044052226) - SDGQMEDLYNEVQK (WP_025406847)** | **-** | **-** | **-** |

**Table S6**. Peptides from tick-borne pathogens were identified in pediatric patients suspected of tick-borne illness. Sample ID = patient identification number, Sex = sex of patient, Age = age of patient, Tick-borne pathogen = organism that had 100% match with the identified peptide, Peptides identified = amino acid sequence of identified peptides.

| **Sample_ID** | **Sex** | **Age** | **Tick-borne pathogen** | **Peptides identified** |
| --- | --- | --- | --- | --- |
| 671900 | 1 | 6 | *Babesia* | QPINSYISDDAESQINNASNMMYK (XP_012649780) |
| 158492 | 1 | 9 | *Babesia* | IAQELSLHDLDTANAKPMSGNDVVSILGQYL (XP_021337180) |
| 226622 | 0 | 17 | *-* | - |
| 152946 | 0 | 8 | *Borrelia* | KDNSKLVEAER (WP_071983715) |
|  |  |  | *Borrelia* | ITAATGCGIPISK (WP_028328339) |
|  |  |  | *Borrelia* | KIENKVGSNAAGT (WP_012622204) |
|  |  |  | *Borrelia* | DKILKNFVFK (WP_029346894) |
|  |  |  | *Rickettsia* | IGRSSLEQAAEK (ABV75938) |
|  |  |  | *Borrelia* | VGTLIYGIFLR (WP_014696474) |
| 245023 | 1 | 12 | *Borrelia* | VYLNNISDLK (B7XS60) |
|  |  |  | *Ehrlichia* | MGVFLLMLSLLMCVR (WP_044170479) |
|  |  |  | *Ehrlichia* | QNITSGSLLDVASGTGDIAIK (WP_024072397) |
| 906453 | 0 | 12 | *Borrelia* | FEEIKIEAR (T1ECH2) |
| 906453 |  |  | *Borrelia* | YKMYDIEDSLEMYSK (A0A1D8TDR2) |
| 906453 |  |  | *Babesia* | NIDESNIEHLK (BMR1_03g00020) |
| 269504 | 1 | 2 | *-* | - |
| 109126 | 1 | 17 | *Borrelia* | GNSIEELKGIK (ACL35186.1) |
| 109126 |  |  | *Borrelia* | DAIMTDEIVASTTNSILTQSAMAMIAQANQV (AAL99373.1) |
| 109126 |  |  | *Borrelia* | FQSVNGDNK (WP_106022635) |

**Table S7.** *Francisella* species and endosymbionts to which six peptides were attributed.

| **Peptide: YLVLGLFMFLYGCAR** |  |
| --- | --- |
| Organism | Peptide percentage identity |
| *Francisella tularensis* | 100% |
| *Francisella hispaniensis* | 100% |
| *Endosymbiont Ornithodoros Moubata* | 93% |
| *Endosymbiont Amblyomma Maculatum* | 93% |
| *Francisella philomiragia* | 87% |
| *Francisella persica* | 87% |
| **Peptide: DAAILSPCDGMVR** |  |
| Organism | Peptide percentage identity |
| *Francisella tularensis* | 100% |
| *Francisella novicida* | 100% |
| *Francisella hispaniensis* | 100% |
| *Francisella philomiragia* | 100% |
| *Francisella persica* | 100% |
| *Endosymbiont Ornithodoros Moubata* | 92% |
| *Endosymbiont Amblyomma Maculatum* | 92% |
| **Peptide: EIQLQQQQNAANQDK** |  |
| Organism | Peptide percentage identity |
| *Francisella tularensis* | 100% |
| *Francisella hispaniensis* | 100% |
| *Endosymbiont Ornithodoros Moubata* | 80% |
|  |  |
| **Peptide: SYMQRYAYRITQEIIK** |  |
| Organism | Peptide percentage identity |
| *Francisella tularensis* | 100% |
| *Francisella philomiragia* | 94% |
| *Francisella hispaniensis* | 94% |
| *Francisella noatunensis* | 94% |
| *Francisella novicida* | 90% |
| *Francisella halioticida* | 88% |
| *Francisella ulginis* | 88% |
| *Francisella adeliensis* | 88% |
| *Allofrancisella inopinata* | 81% |
| *Allofrancisella guangzhouensis* | 81% |
| **Peptide: NLSAAEVIAQLWIAAR** |  |
| Organism | Peptide percentage identity |
| *Francisella tularensis* | 100% |
| *Francisella hispaniensis* | 100% |
| *Francisella persica* | 100% |
| *Francisella philomiragia* | 88% |
|  |  |
| **Peptide: DLEIIDNVGK** |  |
| Organism | Peptide percentage identity |
| *Francisella novicida* | 100% |
| *Francisella hispaniensis* | 100% |
| *Francisella persica* | 100% |
| *Francisella philomiragia* | 90% |
| *Endosymbiont Ornithodoros Moubata* | 90% |
| *Endosymbiont Amblyomma Maculatum* | 90% |
|  |  |
| **Peptide: EYPIKIVESK** |  |
| Organism | Peptide percentage identity |
| *Francisella hispaniensis* | 100% |
| *Endosymbiont Ornithodoros Moubata* | 90% |
| *Endosymbiont Amblyomma Maculatum* | 90% |
| *Francisella persica* | 90% |
| *Francisella philomiragia* | 87% |

**Table S8.** Gene Ontology annotation of 160 proteins from *Borrelia* species. Proteins are grouped according to the biological function.

| **Classification of Borrelia protein according to biological function** | |  |
| --- | --- | --- |
|  |  |  |
| **Biological_function** | **Protein description** | **Number of proteins** |
| Biosynthesis | Adenylosuccinate synthase (WP_028328339), Glutamine-hydrolyzing gmp synthase (WP_029346894), Atp-dependent clp protease, subunit a (EED30070), Rrna maturation rnase ybey (WP_002658307), 16s rrna (cytosine(1402)-n(4))-methyltransferase rsmh (WP_008882360), Type 2 isopentenyl-diphosphate delta-isomerase (WP_081719344), Iron-sulfur cluster assembly scaffold protein (WP_151060615), Nicotinate phosphoribosyltransferase (A0A0A7UVW0), Alanine racemase (WP_110503430), Udp-n-acetylmuramate--l-alanine ligase (A0A0E1U7Y5), Nicotinate (nicotinamide) nucleotide adenylyltransferase (WP_106405031) | 12 |
| Cell cycle | Para family protein (WP_106019052), Para family protein (WP_031558227), Penicillin-binding protein (AFU75021), Septal ring lytic transglycosylase rlpa family protein (WP_031541693), Para family protein (WP_010883900), Chromosome partitioning protein (WP_012621115) | 6 |
| Cell wall organization/biogenesis | Undecaprenyldiphospho-muramoylpentapeptide beta-n-acetylglucosaminyltransferase (WP_106022675), Udp-n-acetylmuramoyl-tripeptide--d-alanyl-d-alanine ligase (WP_010889724), Murein biosynthesis integral membrane protein murj (WP_011194102) | 3 |
| Chemotaxis/motility | Flagellar hook assembly protein flgd (WP_008882406), Chemotaxis protein (WP_025443599), Chemotaxis protein cheb (WP_045245604), Chea (B7XS60), Multispecies: flagellar motor switch protein flin (WP_014696160), Chemotaxis protein chey (A1QZY3), Flagellar protein (Q661Y3), Flagellin protein (BAA82630), Chemotaxis histidine kinase (AFT83983), Flagellar hook capping protein (A0A0H3C216), Chemotaxis protein mota (motility protein a) (WP_002661838), Flagellar basal-body rod protein flgg (W5SD80), Chemotaxis protein chea (WP_075552323), Flagellin, partial (AAL99373.1) | 15 |
| DNA replication/recombination/repair | Excinuclease abc subunit uvra (WP_025434152), Excinuclease abc subunit b (WP_025406847), Exodeoxyribonuclease v alpha chain (W5SCW8), Chromosome replication/partitioning protein (WP_075552597), Excinuclease abc subunit b (WP_071983715), Exodeoxyribonuclease v subunit alpha (WP_071983560), Dna mismatch repair protein muts (A0A0A7UYW5), Dna primase (WP_119024258), Dna polymerase iii subunit beta (WP_025407406), Replicative dna helicase (A0A0E0SSC4), Dna mismatch repair endonuclease mutl (WP_014023462), Dna polymerase i (Q660X6), Dna topoisomerase (atp (WP_106011477), Dna protecting protein dpra (A0A1D8TDQ0), Dna ligase (G0ALZ3), Chromosome partitioning protein parb (A0A0E1BWH2), Dna-directed dna polymerase (A0A0E1UAQ1), Endonuclease muts2 (W5SEH9) | 18 |
| Immune evasion | Variable large protein (WP_144033213), Ospc (fragment) (Q6UR22), Variable large protein (WP_012622204), Vlpa70 (plasmid) (ANA43971), Variable large protein (W5STS8), Outer surface protein b, partial (BAA22351), Vsp protein (B5RP22), Vlpb9 (A0A218PF23), Immunogenic protein p37 (WP_106017067), Variable large protein (W5T0W0), Outer surface protein a (fragment) (O50616), Outer surface protein c (H6VGK4), Variable large protein 15/16 (WP_028328185), Outer surface protein a (BAA19222), Vmps protein (I0FF14), Vlpd4 (A0A218PF75), Outer surface protein c (ospc) (WP_012622036), Variable large protein (I0FEW4), Variable large protein vlpa21h (W5SLA2) | 20 |
| Metabolism | N-acetylmannosamine-6-phosphate 2-epimerase (WP_117375034), Pts mannose transporter subunit iib (WP_014023373), Utp--glucose-1-phosphate uridylyltransferase (WP_020954596), Lipase (WP_106017219), 1-acyl-sn-glycerol-3-phosphate acyltransferase (WP_014023569), Pts glucose transporter subunit iib (WP_014696474), Endopeptidase la (WP_031541621), Glycoside hydrolase family 3 protein (WP_156110316), Arginine deiminase (WP_071983718), Acetate kinase, partial (AGJ81566), Alpha/beta fold hydrolase (WP_117375036), Utp--glucose-1-phosphate uridylyltransferase (WP_029346900), Aminopeptidase (WP_108728866), Hypothetical protein (WP_008882409), Signal peptidase i (WP_014695942), Had family hydrolase (WP_025406568), Superoxide dismutase (A1QYV0), Coa-disulfide reductase (A0A1D8TE94), Pyruvate kinase (A0A0A7V1T0), V-type atp synthase subunit e (WP_106017307), Utp--glucose-1-phosphate uridylyltransferase (WP_012421786), Had family hydrolase (WP_025407384) | 23 |
| Signal transduction | Chemotaxis protein (AJA90394), Phosphatidate cytidylyltransferase (AHH12137), His kinase a domain protein (Q0SN88), Pled family two-component system response regulator (WP_006433838) | 4 |
| Transcription | Atp-dependent rna helicase (WP_106022834), Competence protein (WP_006433983), Transcript cleavage factor (WP_008882341) | 3 |
| Translation/protein processing | Wp_038363604 - translation initiation factor if-2 - [borrelia persica] (WP_038363604), Trna (guanosine(18)-2'-o)-methyltransferase (W5SJU8), Trna(ile)-lysidine synthase (A1R0L1), P-553 (fragment) (Q50E31), Lon protease (A0A1D8TEF9), S41 family peptidase (WP_117374824), Trna n6-adenosine threonylcarbamoyltransferase (A0A0E1U8W9), Arginine--trna ligase (WP_091971958), Peptide chain release factor n(5)-glutamine methyltransferase (WP_012421970) | 9 |
| Transmembrane transport/lipid transport/ion transport | Uracil-xanthine permease (plasmid) (AJA67228), Oligopeptide abc transporter, periplasmic oligopeptide-binding protein (Q6ASN6), Porin (A0A1L6VVD6), Mechanosensitive ion channel family protein (Q0SN56), Borrelia bdr family protein (Q0SMZ6), Acriflavine resistance protein (A0A0E1U8C0), Phage portal protein (A0A172XCQ6), Magnesium transporter mgte (A0A0E1UAY2), Abc transporter substrate-binding protein (A0A0A7UVQ9), P66 protein precursor (AHH03598) | 11 |
| Unknown | Hypothetical protein (WP_064536561), Duf685 domain-containing protein (WP_014023195), Hypothetical membrane associated protein (AAX17020), Hypothetical protein (WP_020732484), Hypothetical protein (WP_106011467), Hypothetical protein (WP_020732373), Hypothetical protein (WP_044052226), Uncharacterized protein (A0A0A7UWS0), Uncharacterized protein (A0A0R9QEI9), Hypothetical protein (WP_044052160), Redox-regulated atpase ychf (WP_031489998), Hypothetical protein (WP_025408373), Uncharacterized protein (W5SQT1), Conserved hypothetical protein (plasmid) (EEH00288), Uncharacterized protein (A0A1D8TDG3), Bax inhibitor-1/ycca family protein (WP_040236434.1), Duf327 family protein (WP_117374741), Uncharacterized protein (A0A0E1U9X9), Hypothetical protein, partial (WP_141631485), Outer surface protein c (Q49579), Putative membrane spanning protein (W6THA6), Hypothetical protein (WP_044051985), Hypothetical protein (WP_088895085), Tetratricopeptide repeat protein (WP_025407311), Uncharacterized protein (A0A1L8Z945), Uncharacterized protein (A0A0E1C0C2), Uncharacterized protein (A0A218PF40), Duf814 domain-containing protein (A0A0E1BXD9), Uncharacterized protein (G0ANW1), Hypothetical protein (WP_117374895), Uncharacterized protein (T1ECH2), Peptidoglycan-binding protein lysm (A0A1D8TDR2), Uncharacterized protein (B7XS64), Bdrc1 (Q1CNV9), Uncharacterized protein (I0FF13), Yigz family protein (WP_008882425), Uncharacterized protein (A0A0E1C250), Hypothetical protein (WP_106019189), Duf244 domain-containing protein, partial (WP_146124657), Uncharacterized protein (W6TH26), Hypothetical protein (WP_002660444), Uncharacterized protein (Q6ASF6), Hypothetical protein (WP_006433391), Uncharacterized protein (C0ANF9), Mlp lipoprotein family-containing protein (I0FEL2), Duf3996 domain-containing protein (WP_073999095), Hypothetical protein (WP_006433624), Hypothetical protein (WP_088895116), Antigen, s2 (ACL35186.1), Response regulator (WP_106022635), Outer surface protein c (CAG47088), Pqq-like beta-propeller repeat protein (WP_002656071), Hypothetical protein (WP_119024075) | 56 |

**Table S9.** Gene Ontology annotation of 160 proteins from *Borrelia* species. Proteins are grouped according to the cellular compartment.

| **Classification of Borrelia protein according to cell compartment** | |  |
| --- | --- | --- |
|  |  |  |
| **Cellular_compartment** | **Protein description** | **Number of proteins** |
| Membrane | Pts mannose transporter subunit iib (WP_014023373), Hypothetical membrane associated protein (AAX17020), Chemotaxis protein (WP_025443599), Undecaprenyldiphospho-muramoylpentapeptide beta-n-acetylglucosaminyltransferase (WP_106022675), Penicillin-binding protein (AFU75021), Variable large protein (WP_144033213), 1-acyl-sn-glycerol-3-phosphate acyltransferase (WP_014023569), Ospc (fragment) (Q6UR22), Chemotaxis protein (AJA90394), Adenylosuccinate synthase (WP_028328339), Pts glucose transporter subunit iib (WP_014696474), Vlpa70 (plasmid) (ANA43971), Phosphatidate cytidylyltransferase (AHH12137), Uracil-xanthine permease (plasmid) (AJA67228), Variable large protein (W5STS8), Oligopeptide abc transporter, periplasmic oligopeptide-binding protein (Q6ASN6), Outer surface protein c (Q49579), Putative membrane spanning protein (W6THA6), Vsp protein (B5RP22), Vlpb9 (A0A218PF23), Porin (A0A1L6VVD6), Immunogenic protein p37 (WP_106017067), Variable large protein (W5T0W0), Hypothetical protein (WP_008882409), Iron-sulfur cluster assembly scaffold protein (WP_151060615), Signal peptidase i (WP_014695942), Peptidoglycan-binding protein lysm (A0A1D8TDR2), Bdrc1 (Q1CNV9), Outer surface protein a (fragment) (O50616), Outer surface protein c (H6VGK4), Mechanosensitive ion channel family protein (Q0SN56), Variable large protein 15/16 (WP_028328185), Outer surface protein a (BAA19222), Borrelia bdr family protein (Q0SMZ6), Murein biosynthesis integral membrane protein murj (WP_011194102), Vmps protein (I0FF14), V-type atp synthase subunit e (WP_106017307), Phage portal protein (A0A172XCQ6), Outer surface protein c (ospc) (WP_012622036), Outer surface protein c (CAG47088) | 41 |
| Outer membrane | Mlp lipoprotein family-containing protein (I0FEL2), Variable large protein (WP_012622204), Septal ring lytic transglycosylase rlpa family protein (WP_031541693), Outer surface protein b, partial (BAA22351), Acriflavine resistance protein (A0A0E1U8C0), Vlpd4 (A0A218PF75), P66 protein precursor (AHH03598), Variable large protein (I0FEW4), Variable large protein vlpa21h (W5SLA2) | 10 |
| Inner membrane | Chemotaxis protein cheb (WP_045245604), His kinase a domain protein (Q0SN88), Magnesium transporter mgte (A0A0E1UAY2), Abc transporter substrate-binding protein (A0A0A7UVQ9) | 4 |
| Cytoplasm | N-acetylmannosamine-6-phosphate 2-epimerase (WP_117375034), Excinuclease abc subunit uvra (WP_025434152), Para family protein (WP_106019052), Para family protein (WP_031558227), Utp--glucose-1-phosphate uridylyltransferase (WP_020954596), Lipase (WP_106017219), Excinuclease abc subunit b (WP_025406847), Exodeoxyribonuclease v alpha chain (W5SCW8), Wp_038363604 - translation initiation factor if-2 - [borrelia persica] (WP_038363604), Chromosome replication/partitioning protein (WP_075552597), Excinuclease abc subunit b (WP_071983715), Glutamine-hydrolyzing gmp synthase (WP_029346894), Exodeoxyribonuclease v subunit alpha (WP_071983560), Dna mismatch repair protein muts (A0A0A7UYW5), Dna primase (WP_119024258), Dna polymerase iii subunit beta (WP_025407406), Endopeptidase la (WP_031541621), Udp-n-acetylmuramoyl-tripeptide--d-alanyl-d-alanine ligase (WP_010889724), Glycoside hydrolase family 3 protein (WP_156110316), Arginine deiminase (WP_071983718), Replicative dna helicase (A0A0E0SSC4), Acetate kinase, partial (AGJ81566), Rrna maturation rnase ybey (WP_002658307), Chea (B7XS60), Atp-dependent rna helicase (WP_106022834), 16s rrna (cytosine(1402)-n(4))-methyltransferase rsmh (WP_008882360), Competence protein (WP_006433983), Transcript cleavage factor (WP_008882341), Dna mismatch repair endonuclease mutl (WP_014023462), Dna polymerase i (Q660X6), Dna topoisomerase (atp (WP_106011477), Para family protein (WP_010883900), Pled family two-component system response regulator (WP_006433838), Had family hydrolase (WP_025406568), Trna (guanosine(18)-2'-o)-methyltransferase (W5SJU8), Trna(ile)-lysidine synthase (A1R0L1), Nicotinate phosphoribosyltransferase (A0A0A7UVW0), Chemotaxis protein chey (A1QZY3), Alanine racemase (WP_110503430), Dna protecting protein dpra (A0A1D8TDQ0), Superoxide dismutase (A1QYV0), P-553 (fragment) (Q50E31), Udp-n-acetylmuramate--l-alanine ligase (A0A0E1U7Y5), Lon protease (A0A1D8TEF9), Coa-disulfide reductase (A0A1D8TE94), Pyruvate kinase (A0A0A7V1T0), S41 family peptidase (WP_117374824), Chromosome partitioning protein (WP_012621115), Dna ligase (G0ALZ3), Trna n6-adenosine threonylcarbamoyltransferase (A0A0E1U8W9), Chromosome partitioning protein parb (A0A0E1BWH2), Dna-directed dna polymerase (A0A0E1UAQ1), Utp--glucose-1-phosphate uridylyltransferase (WP_012421786), Arginine--trna ligase (WP_091971958), Chemotaxis protein chea (WP_075552323), Peptide chain release factor n(5)-glutamine methyltransferase (WP_012421970), Nicotinate (nicotinamide) nucleotide adenylyltransferase (WP_106405031) | 58 |
| Extracellular | Duf814 domain-containing protein (A0A0E1BXD9) | 1 |
| Flagellum | Flagellar hook assembly protein flgd (WP_008882406), Multispecies: flagellar motor switch protein flin (WP_014696160), Flagellar protein (Q661Y3), Flagellin protein (BAA82630), Flagellar hook capping protein (A0A0H3C216), Chemotaxis protein mota (motility protein a) (WP_002661838), Flagellar basal-body rod protein flgg (W5SD80), Flagellin, partial (AAL99373.1) | 9 |
| Nucleoid | Endonuclease muts2 (W5SEH9) | 1 |
| Unknown | Hypothetical protein (WP_064536561), Duf685 domain-containing protein (WP_014023195), Hypothetical protein (WP_020732484), Hypothetical protein (WP_106011467), Hypothetical protein (WP_020732373), Hypothetical protein (WP_044052226), Uncharacterized protein (A0A0A7UWS0), Uncharacterized protein (A0A0R9QEI9), Hypothetical protein (WP_044052160), Redox-regulated atpase ychf (WP_031489998), Hypothetical protein (WP_025408373), Uncharacterized protein (W5SQT1), Conserved hypothetical protein (plasmid) (EEH00288), Uncharacterized protein (A0A1D8TDG3), Bax inhibitor-1/ycca family protein (WP_040236434.1), Duf327 family protein (WP_117374741), Uncharacterized protein (A0A0E1U9X9), Hypothetical protein, partial (WP_141631485), Atp-dependent clp protease, subunit a (EED30070), Hypothetical protein (WP_044051985), Hypothetical protein (WP_088895085), Tetratricopeptide repeat protein (WP_025407311), Alpha/beta fold hydrolase (WP_117375036), Uncharacterized protein (A0A1L8Z945), Uncharacterized protein (A0A0E1C0C2), Uncharacterized protein (A0A218PF40), Type 2 isopentenyl-diphosphate delta-isomerase (WP_081719344), Utp--glucose-1-phosphate uridylyltransferase (WP_029346900), Uncharacterized protein (G0ANW1), Aminopeptidase (WP_108728866), Hypothetical protein (WP_117374895), Uncharacterized protein (T1ECH2), Uncharacterized protein (B7XS64), Uncharacterized protein (I0FF13), Yigz family protein (WP_008882425), Uncharacterized protein (A0A0E1C250), Hypothetical protein (WP_106019189), Duf244 domain-containing protein, partial (WP_146124657), Uncharacterized protein (W6TH26), Hypothetical protein (WP_002660444), Chemotaxis histidine kinase (AFT83983), Uncharacterized protein (Q6ASF6), Hypothetical protein (WP_006433391), Uncharacterized protein (C0ANF9), Duf3996 domain-containing protein (WP_073999095), Hypothetical protein (WP_006433624), Hypothetical protein (WP_088895116), Antigen, s2 (ACL35186.1), Response regulator (WP_106022635), Had family hydrolase (WP_025407384), Pqq-like beta-propeller repeat protein (WP_002656071), Hypothetical protein (WP_119024075) | 56 |

**Table S10:** Number of urinary peptides of non-acute tick-borne disease patients positively correlates with presence or absence of symptoms (p-value <0.01).

| **Model Fitting Information - Correlation of number of positive peptides with symptom severity** | | | | | |
| --- | --- | --- | --- | --- | --- |
| Model | -2 Log Likelihood | Chi-Square | df | Sig. |  |
| Intercept Only | 56.084 |  |  |  |  |
| Final | 44.698 | 11.385 | 3 | 0.01 |  |
| Link function: Logit. |  |  |  |  |  |


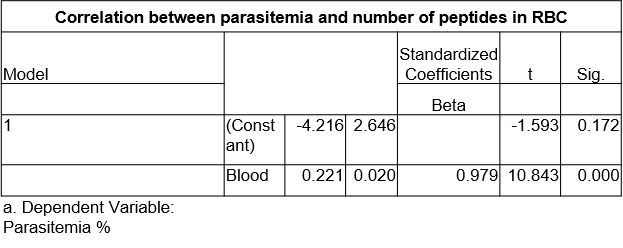

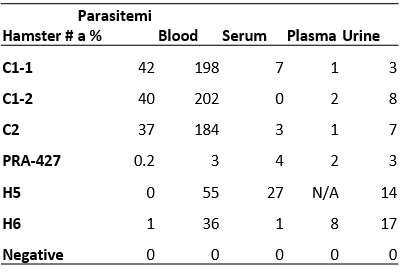
**Table S11.** The number of *Babesia* peptides identified in the hamster RBCs correlated with levels of parasitemia as determined by PCR and IFA. Number of peptides identified in RBC, plasma, serum and urine of C1-1, C1-2, C2, C2-2, H5, and H6 hamsters with different level of parasitemia are reported (left panel). There was a statistically significant correlation between parasitemia levels and number of peptides identified (p < 0.01) (right panel).

**Table S12**. List of *Babesia* peptides identified in hamsters infected with *Babesia microti* and their functional annotation. Gene symbol = protein identifier, Function = function of the protein that unambiguously matched with the identified peptide.

| **Accession** | **Biofluid** | **Translation/ Protein Processing** | **Chaperone** | **Transport** | **Trafficking** | **Metabolism** | **Biosynthesis** | **Membrane/Secreted antigens** | **Motility/Invasion** | **DNA replication/recombination/repair** | **Signal transduction/transcription** | **Transcription** | **Ribosomes** | **Unkown** |
| --- | --- | --- | --- | --- | --- | --- | --- | --- | --- | --- | --- | --- | --- | --- |
| Bmr1_04g08821 | Urine | - |  | - | - | - | - | - | - | - | ✔ | - | - | - |
| Bmr1_02g00395 | Urine | ✔ | - | - | - | - | - | - | - | - | - | - | - | - |
| Bmr1_01g00755 | Urine | - | - | - | - | - | - | - | - | - | - | - | ✔ | - |
| B5rmm2 | Plasma | - | - | - | - | - | - | - | - | - | - | - | - | ✔ |
| Bmr1_03g03551 | Plasma | - | - | - | - | - | - | - | - | - | - | - | - | ✔ |
| Bmr1_01g01220 | RBC | - | - | - | - | - | - | - | - | ✔ | - | - | - | - |
| Bmr1_02g00630 | RBC | ✔ | - | - | - | - | - | - | - | - | - | - | - | - |
| Bmr1_02g01115 | RBC | ✔ | - | - | - | - | - | - | - | - | - | - | - | - |
| Bmr1_03g00785 | Serum | - | - | - | - | - | - | ✔ | - | - | - | - | - | - |
| Bmr1_01g02545 | Serum | - | ✔ | - | - | - | - | - | - | - | - | - | - | - |
| Bmr1_02g02410 | Serum | - | - | - | - | - | - | ✔ | - | - | - | - | - | - |
| Bmr1_01g01945 | Serum | - | - | - | - | - | - | ✔ | - | - | - | - | - | - |
| Bmr1_03g00050 | RBC | - | - | - | - | - | - | - | - | - | - | - | - | ✔ |
| Bmr1_04g08775 | RBC | - | ✔ | - | - | - | - | - | - | - | - | - | - | - |
| Bmr1_04g06050 | RBC | - | ✔ | - | - | - | - | - | - | - | - | - | - | - |
| Bmr1_03g03315 | RBC | - | ✔ | - | - | - | - | - | - | - | - | - | - | - |
| Bmr1_03g04636 | RBC | - | - | ✔ | - | - | - | - | - | - | - | - | - | - |
| Bmr1_03g03720 | RBC | - | - | - | - | - | - | - | ✔ | - | - | - | - | - |
| Bmr1_03g00960 | RBC | - | - | - | - | - | - | ✔ | - | - | - | - | - | - |
| Bmr1_03g00947 | RBC | - | - | - | - | - | - | ✔ | - | - | - | - | - | - |
| Bmr1_04g05965 | RBC | - | - | - | - | ✔ | - | - | - | - | - | - | - | - |
| Bmr1_04g09955 | RBC | ✔ | - | - | - | - | - | - | - | - | - | - | - | - |
| Bmr1_04g07535 | RBC | - | - | - | - | - | - | ✔ | - | - | - | - | - | - |
| Bmr1_01g00230 | RBC | - | - | - | - | ✔ | - | - | - | - | - | - | - | - |
| Bmr1_03g00420 | RBC | - | - | - | - | - | - | - | - | ✔ | - | - | - | - |
| Bmr1_03g01425 | RBC | - | - | - | - | - | - | - | - | - | - | ✔ | - | - |
| Bmr1_02g01750 | RBC | ✔ | - | - | - | - | - | - | - | - | - | - | - | - |
| Bmr1_03g03490 | RBC | ✔ | - | - | - | - | - | - | - | - | - | - | - | - |
| Bmr1_02g01245 | RBC | - | - | ✔ | - | - | - | - | - | - | - | - | - | - |
| Bmr1_02g00655 | RBC | ✔ | - | - | - | - | - | - | - | - | - | - | - | - |
| Bmr1_03g02390 | RBC | - | - | - | - | - | - | - | ✔ | - | - | - | - | - |
| Bmr1_01g03345 | RBC | - | - | - | - | ✔ | - | - | - | - | - | - | - | - |
| Bmr1_01g02927 | RBC | - | - | - | - | - | - | - | - | ✔ | - | - | - | - |
| Bmr1_04g09925 | RBC | ✔ | - | - | - | - | - | - | - | - | - | - | - | - |
| Bmr1_02g01010 | RBC | - | - | - | - | ✔ | - | - | - | - | - | - | - | - |
| Bmr1_03g04270 | RBC | - | ✔ | - | - | - | - | - | - | - | - | - | - | - |
| Bmr1_04g08040 | RBC | ✔ | - | - | - | - | - | - | - | - | - | - | - | - |
| Bmr1_03g00625 | RBC | - | - | - | - | ✔ | - | - | - | - | - | - | - | - |
| Bmr1_04g09475 | RBC | ✔ | - | - | - | - | - | - | - | - | - | - | - | - |
| Bmr1_03g00410 | RBC | - | - | - | - | - | - | - | - | ✔ | - | - | - | - |
| Bmr1_02g02790 | RBC | - | - | - | - | - | - | - | - | - | - | - | ✔ | - |
| Bmr1_04g05540 | RBC | - | - | - | - | - | - | - | - | - | ✔ | - | - | - |
| Bmr1_03g01515 | RBC | ✔ | - | - | - | - | - | - | - | - | - | - | - | - |
| Bmr1_02g03070 | RBC | - | - | - | - | - | - | - | - | - | - | ✔ | - | - |
| Bmr1_02g00670 | RBC | ✔ | - | - | - | - | - | - | - | - | - | - | - | - |
| Bmr1_01g02590 | RBC | - | - | - | - | - | - | - | ✔ | - | - | - | - | - |
| Bmr1_01g02928 | RBC | - | - | - | - | - | - | - | - | ✔ | - | - | - | - |
| Bmr1_03g04240 | RBC | ✔ | - | - | - | - | - | - | - | - | - | - | - | - |
| Bmr1_01g00020 | RBC | - | - | - | - | ✔ | - | - | - | - | - | - | - | - |
| Bmr1_04g10010 | RBC | - | - | - | - | ✔ | - | - | - | - | - | - | - | - |
| Bmr1_02g01635 | RBC | - | - | - | - | - | - | - | - | - | - | - | ✔ | - |
| Bmr1_02g01945 | RBC | - | - | - | - | - | - | - | - | - | - | - | - | ✔ |
| Bmr1_04g06145 | RBC | - | - | - | - | - | - | - | - | ✔ | - | - | - | - |
| Bmr1_02g04275 | RBC | - | - | - | - | - | - | ✔ | - | - | - | - | - | - |
| Bmr1_03g03380 | RBC | - | - | - | - | ✔ | - | - | - | - | - | - | - | - |
| Bmr1_03g00965 | RBC | - | - | - | - | ✔ | - | - | - | - | - | - | - | - |
| Bmr1_04g07427 | RBC | - | - | - | - | - | - | - | - | - | - | ✔ | - | - |
| Bmr1_02g00385 | RBC | - | - | - | - | ✔ | - | - | - | - | - | - | - | - |
| Bmr1_01g01790 | RBC | ✔ | - | - | - | - | - | - | - | - | - | - | - | - |
| Bmr1_02g01925 | RBC | - | - | - | - | - | - | - | - | ✔ | - | - | - | - |
| Bmr1_01g01785 | RBC | - | - | - | - | - | - | - | - | ✔ | - | - | - | - |
| Bmr1_01g02245 | RBC | - | - | - | - | - | - | - | - | - | - | - | - | ✔ |
| Bmr1_04g06985 | RBC | ✔ | - | - | - | - | - | - | - | - | - | - | - | - |
| Bmr1_02g04155 | RBC | - | - | - | - | - | - | - | - | - | - | - | - | ✔ |
| Bmr1_03g03645 | RBC | - | - | - | - | - | - | - | - | - | ✔ | - | - | - |
| Bmr1_02g02475 | RBC | - | - | - | - | - | - | - | - | - | - | - | ✔ | - |
| Bmr1_02g01375 | RBC | - | - | - | - | - | - | - | - | - | - | - | ✔ | - |
| Bmr1_01g02020 | RBC | ✔ | - | - | - | - | - | - | - | - | - | - | - | - |
| Bmr1_03g04460 | RBC | - | - | - | - | - | - | - | - | - | - | ✔ | - | - |
| Bmr1_03g04665 | RBC | - | - | - | - | - | ✔ | - | - | - | - | - | - | - |
| Bmr1_01g02100 | RBC | - | - | - | - | - | - | ✔ | - | - | - | - | - | - |
| Bmr1_04g05600 | RBC | - | - | - | - | - | - | - | - | - | - | ✔ | - | - |
| Bmr1_02g02960 | RBC | - | - | - | ✔ | - | - | - | - | - | - | - | - | - |
| Bmr1_02g02405 | RBC | - | - | ✔ | - | - | - | - | - | - | - | - | - | - |
| Bmr1_01g00920 | RBC | - | - | - | - | - | - | - | - | - | - | - | - | ✔ |
| Bmr1_04g05895 | RBC | - | - | - | - | ✔ | - | - | - | - | - | - | - | - |
| Bmr1_04g08780 | RBC | - | - | - | - | - | - | - | - | ✔ | - | - | - | - |
| Bmr1_04g08505 | RBC | - | - | - | - | ✔ | - | - | - | - | - | - | - | - |
| Bmr1_03g00020 | RBC | - | - | - | - | - | - | ✔ | - | - | - | - | - | - |
| Bmr1_04g07390 | RBC | ✔ | - | - | - | - | - | - | - | - | - | - | - | - |
| Bmr1_02g02745 | RBC | - | - | - | - | - | - | - | ✔ | - | - | - | - | - |
| Bmr1_03g00120 | RBC | - | - | ✔ | - | - | - | - | - | - | - | - | - | - |
| Bmr1_01g02170 | RBC | - | - | - | - | - | - | - | - | - | - | - | ✔ | - |
| Bmr1_03g00105 | RBC | - | - | - | - | - | - | - | - | - | - | - | ✔ | - |
| Bmr1_01g02266 | RBC | - | - | - | - | - | - | - | - | - | - | - | ✔ | - |
| Bmr1_04g09230 | RBC | - | - | - | - | - | ✔ | - | - | - | - | - | - | - |
| Bmr1_04g06010 | RBC | - | - | - | - | ✔ | - | - | - | - | - | - | - | - |
| Bmr1_04g08355 | RBC | - | - | - | - | - | - | - | - | - | - | - | ✔ | - |
| Bmr1_03g03070 | RBC | - | - | - | - | - | - | - | - | - | - | - | ✔ | - |
| Bmr1_04g05045 | RBC | ✔ | - | - | - | - | - | - | - | - | - | - | - | - |
| Bmr1_01g02150 | RBC | ✔ | - | - | - | - | - | - | - | - | - | - | - | - |
| Bmr1_02g01730 | RBC | - | - | - | - | - | - | - | - | - | - | - | ✔ | - |
| Bmr1_01g02125 | RBC | - | - | - | - | ✔ | - | - | - | - | - | - | - | - |
| Bmr1_01g02145 | RBC | - | - | - | - | - | - | - | - | - | - | - | ✔ | - |
| Bmr1_01g01670 | RBC | ✔ | - | - | - | - | - | - | - | - | - | - | - | - |
| Bmr1_04g04887 | RBC | - | ✔ | - | - | - | - | - | - | - | - | - | - | - |
| Bmr1_04g07370 | RBC | - | - | - | - | - | - | - | - | - | - | - | ✔ | - |
| Bmr1_03g01945 | RBC | - | - | ✔ | - | - | - | - | - | - | - | - | - | - |
| Bmr1_02g04210 | RBC | - | - | - | - | - | - | - | - | - | - | - | ✔ | - |
| Bmr1_04g05880 | RBC | - | - | - | - | - | ✔ | - | - | - | - | - | - | - |
| Bmr1_01g00545 | RBC | - | - | - | - | - | - | - | - | - | - | - | ✔ | - |
| Bmr1_02g02535 | RBC | - | - | - | - | - | ✔ | - | - | - | - | - | - | - |
| Bmr1_02g01170 | RBC | - | - | - | - | - | - | - | - | - | - | - | - | ✔ |
| Bmr1_02g01800 | RBC | - | - | - | - | - | - | - | - | - | ✔ | - | - | - |
| Bmr1_04g05335 | RBC | - | - | - | - | - | - | - | - | - | - | - | ✔ | - |
| Bmr1_02g03065 | RBC | - | - | - | - | - | - | - | - | - | - | - | ✔ | - |
| Bmr1_02g01525 | RBC | ✔ | - | - | - | - | - | - | - | - | - | - | - | - |
| Bmr1_04g04880 | RBC | - | - | - | - | - | - | - | - | - | - | - | ✔ | - |
| Bmr1_01g01876 | RBC | - | - | - | - | - | - | - | - | ✔ | - | - | - | - |
| Bmr1_03g03010 | RBC | - | ✔ | - | - | - | - | - | - | - | - | - | - | - |
| Bmr1_03g03885 | RBC | - | - | - | ✔ | - | - | - | - | - | - | - | - | - |
| Bmr1_01g00950 | RBC | - | - | - | - | - | - | - | - | - | - | - | ✔ | - |
| Bmr1_04g05710 | RBC | ✔ | - | - | - | - | - | - | - | - | - | - | - | - |
| Bmr1_04g08767 | RBC | ✔ | - | - | - | - | - | - | - | - | - | - | - | - |
| Bmr1_04g05340 | RBC | - | - | - | - | - | - | - | - | - | - | - | - | ✔ |
| Bmr1_04g08390 | RBC | - | - | - | - | - | - | - | - | - | - | - | - | ✔ |
| Bmr1_01g02090 | RBC | - | - | - | - | - | - | - | - | - | - | - | ✔ | - |
| Bmr1_04g06330 | RBC | - | - | - | - | ✔ | - | - | - | - | - | - | - | - |
| Bmr1_03g00820 | RBC | - | - | - | - | - | - | ✔ | - | - | - | - | - | - |
| Bmr1_04g06937 | RBC | - | - | - | - | - | - | - | - | - | - | - | ✔ | - |
| Bmr1_03g04810 | RBC | ✔ | - | - | - | - | - | - | - | - | - | - | - | - |
| Bmr1_03g00980 | RBC | - | - | - | - | - | - | - | - | - | - | ✔ | - | - |
| Bmr1_03g00800 | RBC | - | - | - | - | - | - | - | ✔ | - | - | - | - | - |
| Bmr1_02g01630 | RBC | - | - | - | - | - | - | - | - | - | - | - | ✔ | - |
| Bmr1_04g05480 | RBC | ✔ | - | - | - | - | - | - | - | - | - | - | - | - |
| Bmr1_01g01665 | RBC | - | - | - | - | - | - | - | - | - | - | ✔ | - | - |
| Bmr1_04g05465 | RBC | - | - | - | - | - | - | - | - | - | - | - | - | ✔ |
| Bmr1_03g03035 | RBC | - | - | - | - | - | - | - | - | - | - | - | ✔ | - |
| Bmr1_04g07556 | RBC | - | - | - | - | - | - | ✔ | - | - | - | - | - | - |
| Bmr1_04g06300 | RBC | - | - | - | - | - | - | ✔ | - | - | - | - | - | - |
| Bmr1_04g08675 | RBC | - | - | - | - | - | - | - | - | - | - | ✔ | - | - |
| Bmr1_02g01100 | RBC | - | - | - | - | - | - | ✔ | - | - | - | - | - | - |
| Bmr1_04g09385 | RBC | - | - | - | - | ✔ | - | - | - | - | - | - | - | - |
| Bmr1_01g02591 | RBC | - | - | - | - | - | - | - | - | - | - | - | - | ✔ |
| Bmr1_03g04695 | RBC | - | - | - | - | - | - | - | ✔ | - | - | - | - | - |
| Bmr1_01g01705 | RBC | - | - | - | - | - | - | - | - | - | - | - | ✔ | - |
| Bmr1_02g01020 | RBC | - | - | - | - | - | - | - | - | - | - | - | ✔ | - |
| Bmr1_03g00745 | RBC | - | - | - | - | - | - | - | - | - | - | ✔ | - | - |
| Bmr1_01g02075 | RBC | - | - | - | - | ✔ | - | - | - | - | - | - | - | - |
| Bmr1_04g06165 | RBC | - | - | - | - | - | - | - | - | - | - | - | ✔ | - |
| Bmr1_04g06615 | RBC | - | - | - | - | - | - | - | - | - | - | ✔ | - | - |
| Bmr1_02g04195 | RBC | - | - | - | - | - | - | - | - | - | - | ✔ | - | - |
| Bmr1_04g06555 | RBC | - | - | - | - | - | - | - | - | - | - | - | ✔ | - |
| Bmr1_02g02330 | RBC | - | - | - | - | - | - | - | - | ✔ | - | - | - | - |
| Bmr1_03g00665 | RBC | - | ✔ | - | - | - | - | - | - | - | - | - | - | - |
| Bmr1_04g07429 | RBC | ✔ | - | - | - | - | - | - | - | - | - | - | - | - |
| Bmr1_04g05060 | RBC | - | - | - | - | - | - | - | - | - | ✔ | - | - | - |
| Bmr1_04g05265 | RBC | - | - | - | - | - | - | - | - | ✔ | - | - | - | - |
| Bmr1_04g06705 | RBC | - | - | - | - | - | - | ✔ | - | - | - | - | - | - |
| Bmr1_02g02830 | RBC | - | - | - | - | ✔ | - | - | - | - | - | - | - | - |
| Bmr1_03g01040 | RBC | - | - | - | - | - | - | ✔ | - | - | - | - | - | - |
| Bmr1_02g03605 | RBC | - | - | - | - | - | - | - | - | ✔ | - | - | - | - |
| Bmr1_03g00570 | RBC | - | - | - | - | - | - | - | ✔ | - | - | - | - | - |
| Bmr1_03g03605 | RBC | - | - | - | - | - | ✔ | - | - | - | - | - | - | - |
| Bmr1_02g00865 | RBC | - | - | - | - | - | - | - | - | - | - | - | ✔ | - |
| Bmr1_03g02981 | RBC | - | - | - | - | - | - | - | - | - | - | - | ✔ | - |
| Bmr1_03g03280 | RBC | - | - | - | - | - | - | - | - | - | - | ✔ | - | - |
| Bmr1_03g00862 | RBC | - | - | - | - | - | - | - | - | - | ✔ | - | - | - |
| Bmr1_02g01560 | RBC | - | - | - | - | ✔ | - | - | - | - | - | - | - | - |
| Bmr1_02g00870 | RBC | - | - | - | - | - | - | - | - | - | - | - | ✔ | - |
| Bmr1_02g02945 | RBC | - | - | - | - | ✔ | - | - | - | - | - | - | - | - |
| Bmr1_03g00840 | RBC | ✔ | - | - | - | - | - | - | - | - | - | - | - | - |
| Bmr1_04g08340 | RBC | - | - | - | - | - | - | - | - | - | - | - | ✔ | - |
| Bmr1_04g08285 | RBC | - | - | - | - | - | - | - | - | ✔ | - | - | - | - |
| Bmr1_02g00480 | RBC | - | - | - | - | - | - | - | - | - | - | - | ✔ | - |
| Bmr1_03g02466 | RBC | - | - | - | - | ✔ | - | - | - | - | - | - | - | - |
| Bmr1_02g02525 | RBC | - | - | - | - | ✔ | - | - | - | - | - | - | - | - |
| Bmr1_04g06310 | RBC | - | - | - | - | - | - | - | - | - | - | - | - | ✔ |
| Bmr1_03g01380 | RBC | - | - | - | - | ✔ | - | - | - | - | - | - | - | - |
| Bmr1_04g06405 | RBC | - | - | - | - | ✔ | - | - | - | - | - | - | - | - |
| Bmr1_02g01021 | RBC | - | - | - | - | - | - | - | - | - | - | - | ✔ | - |
| Bmr1_02g02185 | RBC | ✔ | - | - | - | - | - | - | - | - | - | - | - | - |
| Bmr1_04g09097 | RBC | - | - | - | - | - | ✔ | - | - | - | - | - | - | - |
| Bmr1_03g04765 | RBC | - | - | - | - | - | - | - | ✔ | - | - | - | - | - |
| Bmr1_03g00640 | RBC | - | - | - | - | - | ✔ | - | - | - | - | - | - | - |
| Bmr1_03g00260 | RBC | - | - | - | - | - | - | ✔ | - | - | - | - | - | - |
| Bmr1_02g01901 | RBC | - | - | - | - | - | - | ✔ | - | - | - | - | - | - |
| Bmr1_04g06445 | RBC | - | - | - | - | - | - | - | - | - | - | ✔ | - | - |
| Bmr1_01g01445 | RBC | ✔ | - | - | - | - | - | - | - | - | - | - | - | - |
| Bmr1_02g02895 | RBC | - | - | - | - | - | - | - | - | ✔ | - | - | - | - |
| Bmr1_04g08980 | RBC | - | - | - | - | - | - | - | - | - | - | - | ✔ | - |
| Bmr1_04g08660 | RBC | - | - | - | - | - | - | - | - | - | - | - | ✔ | - |
| Bmr1_03g04551 | RBC | - | - | - | - | - | - | - | - | ✔ | - | - | - | - |
| Bmr1_01g02200 | RBC | - | - | - | - | - | - | - | - | - | - | - | ✔ | - |
| Bmr1_02g01830 | RBC | - | - | - | - | - | ✔ | - | - | - | - | - | - | - |
| Bmr1_03g03440 | RBC | - | - | - | - | ✔ | - | - | - | - | - | - | - | - |
| Bmr1_01g03005 | RBC | - | - | - | - | - | - | - | - | ✔ | - | - | - | - |
| Bmr1_04g06170 | RBC | - | - | - | ✔ | - | - | - | - | - | - | - | - | - |
| Bmr1_03g03345 | RBC | - | - | - | - | - | - | - | - | ✔ | - | - | - | - |
| Bmr1_02g00945 | RBC | ✔ | - | - | - | - | - | - | - | - | - | - | - | - |
| Bmr1_04g04886 | RBC | - | - | - | - | - | - | - | - | - | - | - | ✔ | - |
| Bmr1_03g04196 | RBC | - | - | - | - | - | - | ✔ | - | - | - | - | - | - |
| Bmr1_01g01920 | RBC | - | - | - | ✔ | - | - | - | - | - | - | - | - | - |
| Bmr1_03g04795 | RBC | - | - | ✔ | - | - | - | - | - | - | - | - | - | - |
| Bmr1_01g01580 | RBC | - | - | - | - | - | - | - | - | - | - | ✔ | - | - |
| Bmr1_03g02445 | RBC | - | - | - | - | - | - | - | - | - | - | - | - | ✔ |
| Bmr1_02g04180 | RBC | - | - | - | - | - | - | - | ✔ | - | - | - | - | - |
| Bmr1_03g01800 | RBC | - | - | - | - | ✔ | - | - | - | - | - | - | - | - |
| Bmr1_03g00550 | RBC | - | - | - | - | - | - | - | - | - | - | ✔ | - | - |
| Bmr1_01g00375 | RBC | - | - | - | - | - | - | - | - | - | - | - | - | ✔ |
| Bmr1_03g00685 | RBC | - | - | - | - | - | - | - | - | - | - | ✔ | - | - |
| Bmr1_03g02665 | RBC | - | - | - | - | - | - | - | - | - | - | - | - | ✔ |
| Bmr1_03g01320 | RBC | - | - | - | - | - | - | ✔ | - | - | - | - | - | - |
| Bmr1_04g05975 | RBC | - | - | - | - | - | - | - | - | - | - | - | - | ✔ |
| Bmr1_03g03990 | RBC | - | - | - | - | ✔ | - | - | - | - | - | - | - | - |
| Bmr1_04g10000 | RBC | - | - | - | - | - | ✔ | - | - | - | - | - | - | - |
| Bmr1_02g04285 | Serum | - | - | - | - | - | - | - | - | - | - | - | - | ✔ |
| Bmr1_03g04683 | Serum | - | - | - | - | - | - | - | - | - | - | - | - | ✔ |
| Bmr1_03g04160 | Serum | - | - | - | - | - | ✔ | - | - | - | - | - | - | - |
| Bmr1_03g02890 | Urine | - | - | - | - | - | - | - | - | - | - | - | - | ✔ |
| Bmr1_01g01585 | Urine | - | - | - | - | ✔ | - | - | - | - | - | - | - | - |
| Bmr1_03g04255 | Urine | - | - | - | - | - | - | - | - | - | - | - | - | ✔ |
| Bmr1_04g07140 | Urine | - | - | - | - | - | - | ✔ | - | - | - | - | - | - |
| Bmr1_02g00750 | RBC | - | - | - | ✔ | - | - | - | - | - | - | - | - | - |
| Bmr1_03g04150 | RBC | - | - | - | - | - | - | - | - | - | - | - | - | ✔ |
| Bmr1_04g08010 | RBC | - | - | - | - | - | - | - | - | - | - | - | ✔ | - |
| Bmr1_04g09685 | RBC | - | - | - | - | - | - | - | - | - | - | - | ✔ | - |
| Bmr1_04g08315 | RBC | - | - | - | - | - | - | - | - | - | - | - | ✔ | - |
| Bmr1_02g03935 | RBC | - | - | - | ✔ | - | - | - | - | - | - | - | - | - |
| Bmr1_04g05605 | RBC | - | - | ✔ | - | - | - | - | - | - | - | - | - | - |
| Bmr1_04g05565 | RBC | - | - | - | - | - | - | - | - | - | ✔ | - | - | - |
| Bmr1_03g00720 | RBC | - | - | - | - | - | - | - | - | ✔ | - | - | - | - |
| Bmr1_04g08896 | RBC | - | - | - | - | - | - | - | - | - | - | - | - | ✔ |
| Bmr1_04g05530 | RBC | - | - | - | - | ✔ | - | - | - | - | - | - | - | - |
| Bmr1_03g04165 | RBC | ✔ | - | - | - | - | - | - | - | - | - | - | - | - |
| Bmr1_03g01090 | RBC | - | - | - | - | - | - | - | - | - | - | - | - | ✔ |
| Bmr1_03g01335 | RBC | - | - | - | - | - | - | ✔ | - | - | - | - | - | - |
| Bmr1_03g04600 | RBC | - | - | - | - | ✔ | - | - | - | - | - | - | - | - |
| Bmr1_02g01320 | RBC | - | - | - | - | - | - | - | - | - | ✔ | - | - | - |
| Bmr1_03g02172 | RBC | - | - | - | - | - | - | - | - | - | - | - | - | ✔ |
| Bmr1_04g08155 | RBC | - | - | - | - | - | - | ✔ | - | - | - | - | - | - |
| Bmr1_02g04221 | RBC | ✔ | - | - | - | - | - | - | - | - | - | - | - | - |
| Bmr1_02g02985 | RBC | - | - | - | - | ✔ | - | - | - | - | - | - | - | - |
| Bmr1_01g01500 | RBC | - | - | - | - | - | - | - | - | - | - | - | - | ✔ |
| Bmr1_03g00680 | RBC | - | - | - | - | - | - | - | - | - | ✔ | - | - | - |
| Bmr1_01g01325 | RBC | - | - | - | - | - | - | ✔ | - | - | - | - | - | - |
| Bmr1_02g01597 | RBC | - | - | - | - | - | - | - | - | - | - | - | ✔ | - |
| Bmr1_04g07490 | RBC | - | - | - | - | - | - | - | - | - | ✔ | - | - | - |
| Bmr1_02g01585 | RBC | - | - | - | - | - | - | - | - | - | ✔ | - | - | - |
| Bmr1_03g00768 | RBC | - | - | - | - | ✔ | - | - | - | - | - | - | - | - |
| Bmr1_04g09805 | RBC | ✔ | - | - | - | - | - | - | - | - | - | - | - | - |
| Bmr1_03g04625 | RBC | ✔ | - | - | - | - | - | - | - | - | - | - | - | - |
| Bmr1_03g02980 | RBC | - | - | - | ✔ | - | - | - | - | - | - | - | - | - |
| Bmr1_04g08375 | RBC | - | - | ✔ | - | - | - | - | - | - | - | - | - | - |
| Bmr1_03g02990 | RBC | ✔ | - | - | - | - | - | - | - | - | - | - | - | - |
| Bmr1_03g03625 | RBC | - | - | - | - | - | - | - | - | - | ✔ | - | - | - |
| Bmr1_01g03370 | RBC | - | - | - | - | - | - | - | - | - | - | - | - | ✔ |
| Bmr1_02g00850 | RBC | - | - | - | - | - | - | - | - | - | - | ✔ | - | - |
| Bmr1_04g09650 | RBC | ✔ | - | - | - | - | - | - | - | - | - | - | - | - |
| Bmr1_02g03525 | RBC | - | - | - | ✔ | - | - | - | - | - | - | - | - | - |
| Bmr1_04g05940 | RBC | - | - | - | - | - | - | - | - | - | - | - | ✔ | - |
| Bmr1_04g08730 | RBC | - | - | - | - | - | - | - | - | - | - | - | ✔ | - |
| Bmr1_02g03980 | RBC | - | - | - | - | - | - | - | - | - | - | - | ✔ | - |
| Bmr1_01g01955 | RBC | - | - | - | - | - | - | - | - | - | - | - | ✔ | - |
| Bmr1_04g09445 | RBC | - | - | - | - | - | - | - | - | - | ✔ | - | - | - |
| Bmr1_03g00380 | RBC | - | - | - | - | - | - | - | - | ✔ | - | - | - | - |
| Bmr1_03g00610 | RBC | - | - | - | - | ✔ | - | - | - | - | - | - | - | - |
| Bmr1_03g03170 | RBC | - | - | - | - | - | - | - | - | - | - | - | - | ✔ |
| Bmr1_03g01130 | RBC | - | - | - | - | - | - | - | - | - | - | - | ✔ | - |
| Bmr1_02g02155 | RBC | ✔ | - | - | - | - | - | - | - | - | - | - | - | - |
| Bmr1_04g07030 | RBC | - | - | - | - | - | - | - | - | - | ✔ | - | - | - |
| Bmr1_03g02910 | RBC | - | - | - | - | - | - | - | - | - | - | - | - | ✔ |
| Bmr1_04g09720 | RBC | - | - | - | - | - | ✔ | - | - | - | - | - | - | - |
| Bmr1_04g07750 | RBC | - | - | - | - | - | - | - | - | - | - | - | ✔ | - |
| Bmr1_03g04265 | RBC | - | - | - | - | ✔ | - | - | - | - | - | - | - | - |
| Bmr1_04g07925 | RBC | - | - | - | - | ✔ | - | - | - | - | - | - | - | - |
| Bmr1_02g01870 | RBC | - | - | - | - | - | - | - | - | - | - | - | ✔ | - |
| Bmr1_01g03115 | RBC | - | - | - | - | - | - | - | - | - | ✔ | - | - | - |
| Bmr1_01g00125 | RBC | - | - | - | - | - | - | - | - | - | - | ✔ | - | - |
| Bmr1_03g00030 | RBC | ✔ | - | - | - | - | - | - | - | - | - | - | - | - |
| Bmr1_04g05120 | RBC | - | - | - | - | - | - | - | - | - | - | ✔ | - | - |
| Bmr1_04g06980 | RBC | - | - | - | - | - | - | - | - | - | - | ✔ | - | - |
| Bmr1_03g01095 | RBC | - | - | - | - | - | - | - | - | - | - | - | - | ✔ |
| Bmr1_01g02030 | RBC | - | - | - | - | - | - | - | - | - | - | - | - | ✔ |
| Bmr1_03g02600 | RBC | - | - | - | - | - | - | - | - | - | - | ✔ | - | - |
| Bmr1_02g04090 | RBC | - | - | - | - | ✔ | - | - | - | - | - | - | - | - |
| Bmr1_03g04215 | RBC | - | - | - | ✔ | - | - | - | - | - | - | - | - | - |
| Bmr1_02g01625 | RBC | - | - | - | - | - | - | - | - | - | ✔ | - | - | - |
| Bmr1_01g02635 | RBC | - | - | - | - | ✔ | - | - | - | - | - | - | - | - |
| Bmr1_01g03280 | RBC | - | - | - | - | - | - | ✔ | - | - | - | - | - | - |
| Bmr1_02g03465 | RBC | - | - | - | - | ✔ | - | - | - | - | - | - | - | - |
| Bmr1_02g00530 | RBC | - | - | - | - | ✔ | - | - | - | - | - | - | - | - |
| Bmr1_04g06995 | RBC | - | - | - | - | - | - | - | - | - | - | - | ✔ | - |
| Bmr1_03g00577 | RBC | - | - | - | - | - | - | ✔ | - | - | - | - | - | - |
| Bmr1_01g00481 | RBC | - | - | - | - | - | - | - | - | - | - | - | - | ✔ |
| Bmr1_03g01820 | RBC | - | - | - | - | ✔ | - | - | - | - | - | - | - | - |
| Bmr1_04g06490 | RBC | - | - | - | ✔ | - | - | - | - | - | - | - | - | - |
| Bmr1_03g01940 | RBC | ✔ | - | - | - | - | - | - | - | - | - | - | - | - |
| Bmr1_03g01795 | Plasma | - | - | - | - | - | - | - | - | - | ✔ | - | - | - |
| Bmr1_01g00005 | Serum | - | - | - | - | - | - | ✔ | - | - | - | - | - | - |
| Bmr1_04g05155 | Urine | - | - | - | - | - | ✔ | - | - | - | - | - | - | - |
| Bmr1_04g05290 | Urine | - | - | ✔ | - | - | - | - | - | - | - | - | - | - |
| Bmr1_01g02877 | Urine | - | - | - | - | - | - | - | - | ✔ | - | - | - | - |
| Bmr1_03g00205 | Plasma | - | - | - | - | - | - | ✔ | - | - | - | - | - | - |
| Bmr1_02g00885 | RBC | - | - | - | - | - | - | - | - | - | - | - | - | ✔ |
| Bmr1_01g02945 | RBC | - | - | - | - | - | - | - | - | - | - | - | ✔ | - |
| Bmr1_03g01405 | RBC | ✔ | - | - | - | - | - | - | - | - | - | - | - | - |
| Bmr1_02g00625 | RBC | - | - | - | - | - | - | - | ✔ | - | - | - | - | - |
| Bmr1_03g01645 | RBC | ✔ | - | - | - | - | - | - | - | - | - | - | - | - |
| Bmr1_04g07585 | RBC | - | - | - | - | - | - | - | - | - | - | ✔ | - | - |
| Bmr1_04g05350 | RBC | - | - | - | - | - | - | - | - | - | - | - | - | ✔ |
| Bmr1_01g00310 | RBC | - | - | - | ✔ | - | - | - | - | - | - | - | - | - |
| Bmr1_03g03525 | RBC | - | - | - | - | - | - | ✔ | - | - | - | - | - | - |
| Bmr1_03g04505 | RBC | ✔ | - | - | - | - | - | - | - | - | - | - | - | - |
| Bmr1_02g03930 | RBC | - | - | - | - | - | - | - | - | - | ✔ | - | - | - |
| Bmr1_01g01121 | RBC | - | - | - | - | - | - | - | - | - | - | - | - | ✔ |
| Bmr1_04g05175 | RBC | - | - | - | - | - | - | - | - | - | - | ✔ | - | - |
| Bmr1_02g02930 | RBC | - | - | - | - | - | ✔ | - | - | - | - | - | - | - |
| Bmr1_02g01305 | RBC | ✔ | - | - | - | - | - | - | - | - | - | - | - | - |
| Bmr1_01g01775 | RBC | - | - | - | - | - | - | - | - | ✔ | - | - | - | - |
| Bmr1_03g03265 | RBC | - | - | - | - | - | - | - | - | - | - | ✔ | - | - |
| Bmr1_04g06160 | RBC | - | - | - | - | ✔ | - | - | - | - | - | - | - | - |
| Bmr1_01g02080 | RBC | - | - | - | - | - | - | - | - | - | - | - | - | ✔ |
| Bmr1_04g05635 | RBC | - | - | - | - | - | - | ✔ | - | - | - | - | - | - |
| Bmr1_02g02805 | RBC | - | - | - | - | - | - | - | - | - | - | - | - | ✔ |
| Bmr1_01g00410 | RBC | ✔ | - | - | - | - | - | - | - | - | - | - | - | - |
| Bmr1_04g05780 | RBC | - | - | - | - | - | - | - | - | - | - | - | - | ✔ |
| Bmr1_04g06845 | RBC | - | - | - | - | - | - | - | ✔ | - | - | - | - | - |
| Bmr1_04g05150 | RBC | ✔ | - | - | - | - | - | - | - | - | - | - | - | - |

**Table S13.** Seroreactive *Babesia* proteins identified in the blood and urine of a hamster animal model. Proteins known to be immunoreactive and including GPI-anchored, secreted and transmembrane proteins, were found in different body fluids: RBCs (BMR1_03g00785; BMR1_03g04695; BMR1_03g00947; BMR1_02g04275; BmR1_04g07535; BmR1_04g07556), serum (BMR1_03g00785; BMR1_03g04695; BMR1_03g00947; BMR1_02g04275; BmR1_04g07535), plasma (BMR1_03g00785; BMR1_03g00947), urine (BMR1_03g00785, BMR1_02g04275) of hamsters infected with *Babesia*. Y = Yes, - = No.

| **Seroreactive antigens** | **IgG response** | **IgM response** | **RBC** | **Serum** | **Plasma** | **Urine** |
| --- | --- | --- | --- | --- | --- | --- |
| BmGPI12 (BMR1_03g00785) | Y | Y | Y | Y | Y | Y |
| Rhoptry neck protein 2 (BMR1_03g04695) | Y | Y | Y | Y | - | - |
| Conserved Plasmodium membrane protein (BMR1_03g00947) | Y | Y | Y | Y | Y | - |
| BmGPI10 (BMR1_02g04275) | Y | - | Y | Y | - | Y |
| N1-15 protein, maltese-cross seroactive antigen (BmR1_04g07535) | Y | - | Y | Y | - | - |
| hypothetical protein (BmR1_04g07556) | - | Y | Y | - | - | - |

# Supplementary Figures


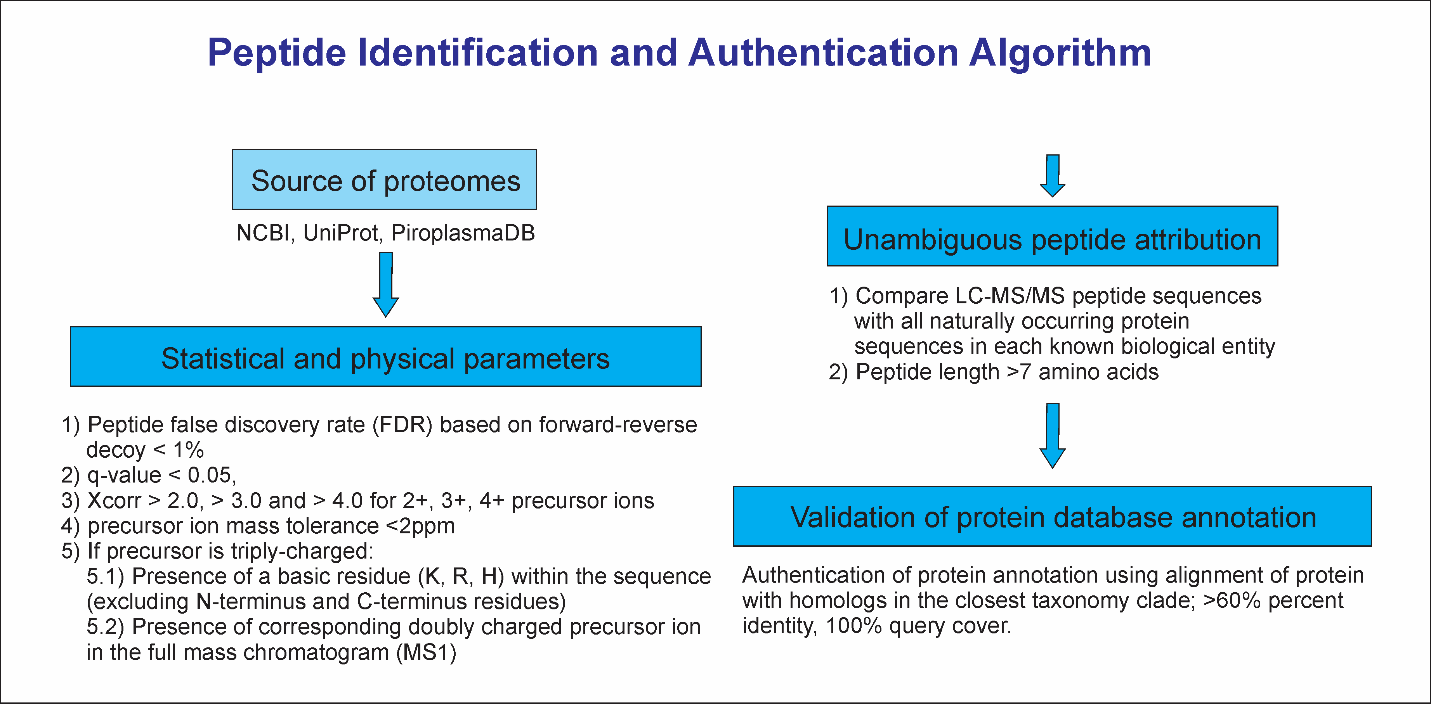


**Fig. S1**. Bioinformatics pipeline for peptide identification and authentication. Appropriate databases were obtained from NCBI, UniProt, PiroplasmaDB. Parameters for spectrum matching were established on a training set of n=110 samples. A series of BLAST analyses was performed in order to unambiguously attribute every peptide to specific microorganisms known to be pathogenic in humans. Finally, authentication of the protein database annotation was performed by aligning the sequence of full length homologous proteins in an evolutionarily related clade. The annotation was considered acceptable if the protein demonstrated greater than 60% homology, over the full query length, with other species in the query.


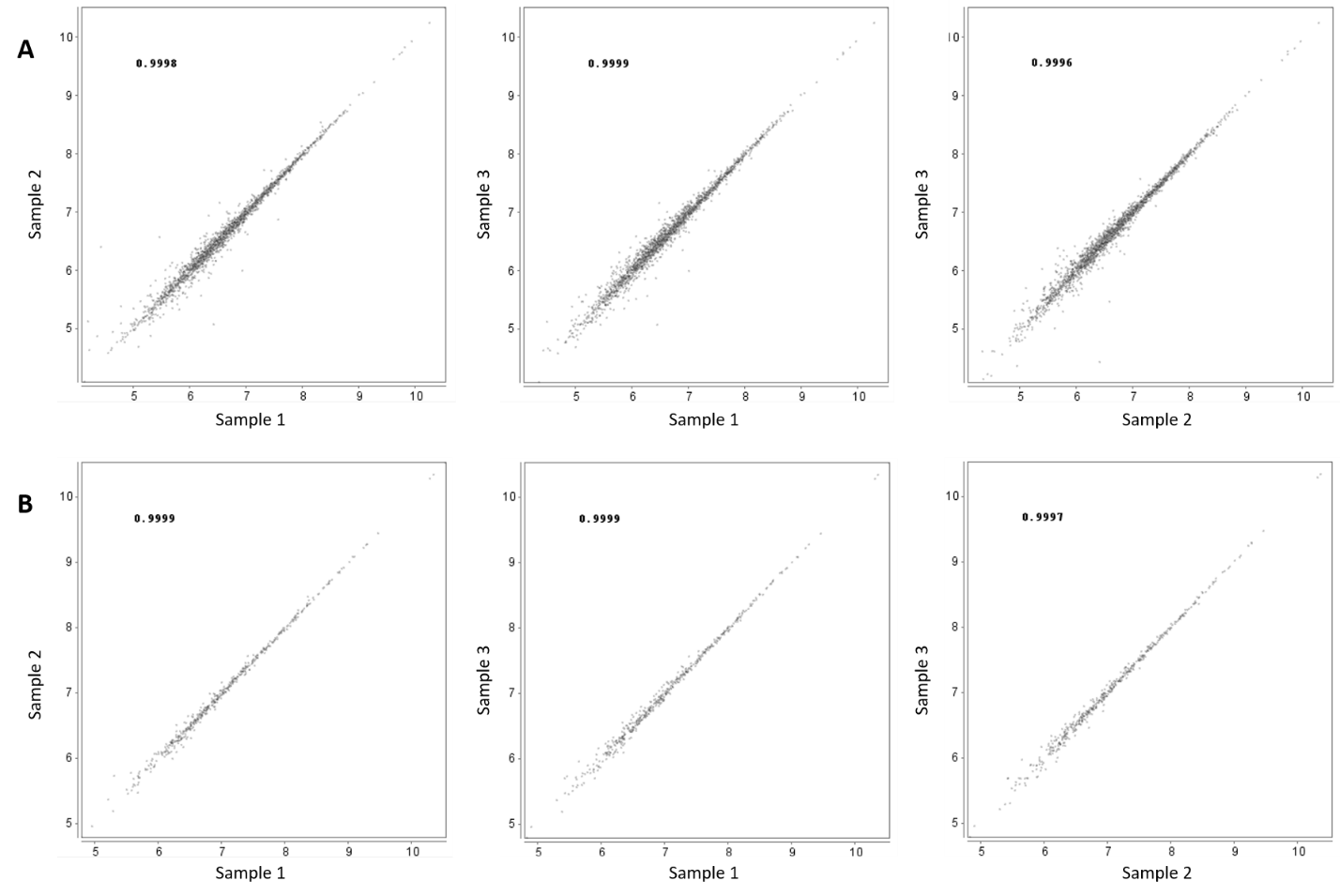


**Fig. S2.** Affinity nanoparticle-mass spectrometry experimental workflow is highly reproducible. Correlation plots obtained via label-free quantification (Peaks Studio) of the MS1 data from 3 technical replicates are shown. Quantification based on peptide (A) and proteins (B) features are reported. Pearson correlation scores are reported and indicate high reproducibility of the experimental workflow in pairwise comparisons.

**Fig. S3.** The number of urinary peptides derived from tick borne pathogens was compared in cases versus controls using the Wilcoxon – Mann – Whitney test (p-value = 2.2E-16). Given the large effect size (3.35) even a low number of observations in the Lyme group (10) and 100 observations in the control group yielded a power of 0.999 with alpha = 0.05. Calculations were conducted using the method of the asymptotic relative efficiency that defines power of the Wilcoxon – Mann – Whitney test relative to the one sample t test. The value of the asymptotic relative efficiency was set at 0.864, the theoretical minimum for the Wilcoxon test, thus providing a conservative estimate of the power. The graph shows how the statistical power varies with the number of observations per group, given effect sizes of 3.35, 2.1 and 0.8.


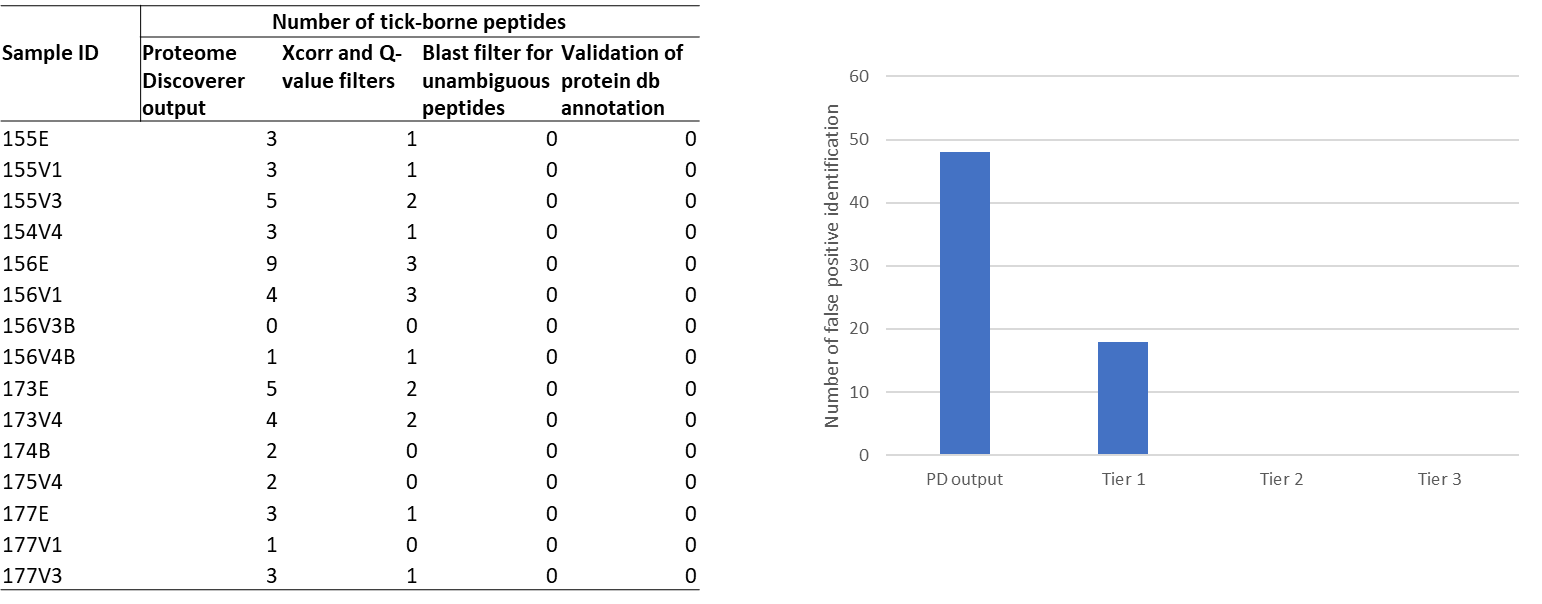


**Fig. S4**. Peptide authentication algorithm reduces the number of false positive peptide identifications. 100 non-Lyme samples in the training set were used to tune the parameters of the peptide discovery and authentication algorithm. Example 15 samples are represented here to illustrate the reduction of false positive peptide identifications through each step of the 3-tier algorithm. Most samples show at least one peptide identification as a result of Proteome Discoverer output. The first tier of the algorithm consists in filtering spectrum matching peptides according to their Xcorr and q-value. The second tier of the algorithm is a series of BLAST analyses against the NCBI Reference Sequence database (RefSeq). The third and last step is a validation of the protein database. 150 independent non-TBI samples in the validation set were subjected to this algorithm and yielded a false positive rate of 0% (based on two peptide per organism cut-off).


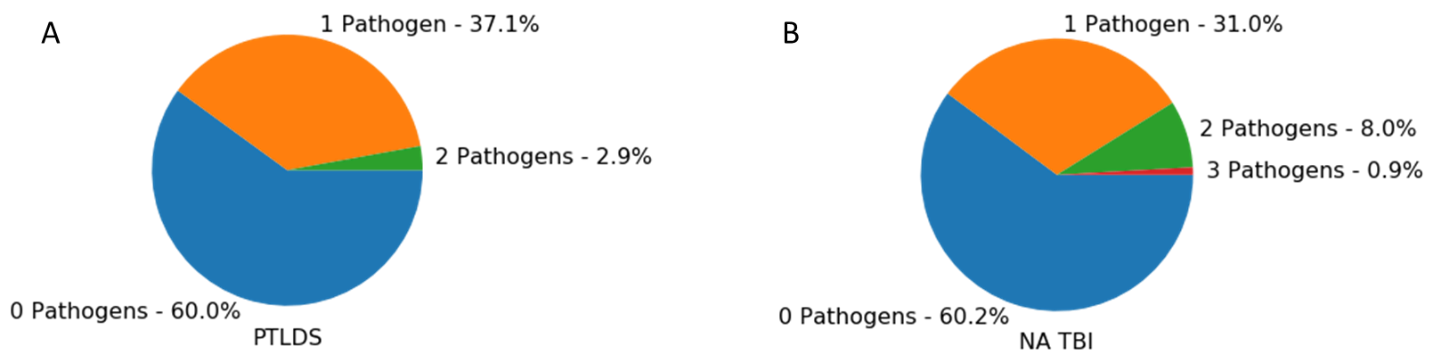


**Fig. S5**. Distribution of pathogen derived, urinary peptides is equivalent in post treatment Lyme disease syndrome (PTLDS) patients and in non-acute, tick-borne disease (NA TBI) patients. A) Pathogen peptides were found in the urine of 40% of patients with PTLDS as well as in patients suspected of tick-borne infections (NA TBI). 37% of PTLDS patients had urinary peptides deriving from one pathogen, and 2.9% from two pathogens. 31% of NA TBI patients had urinary peptides deriving from one pathogen, 8% from two pathogens, and 0.9% from three pathogens.


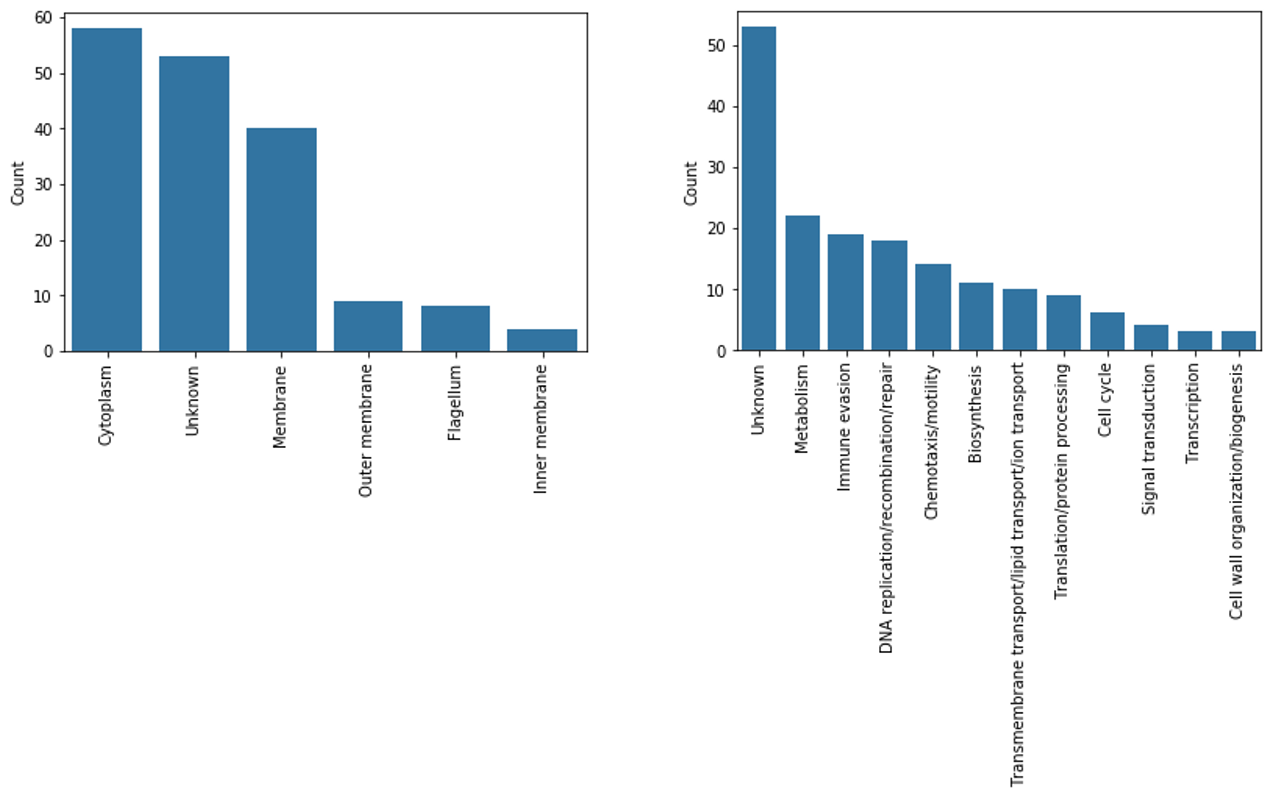


**Fig. S6.** Biological process and cellular component of *Borrelia* derived, urinary peptides identified in the urine of acute LD and non acute patients. Gene Ontology (GO) annotation of 112 proteins from *Borrelia sp*. causing Lyme borreliosis is reported. GO was obtained directly from Uniprot or inferred by homology. Biological process (left) and cellular compartment (right) annotation shows a great diversity in both categories.


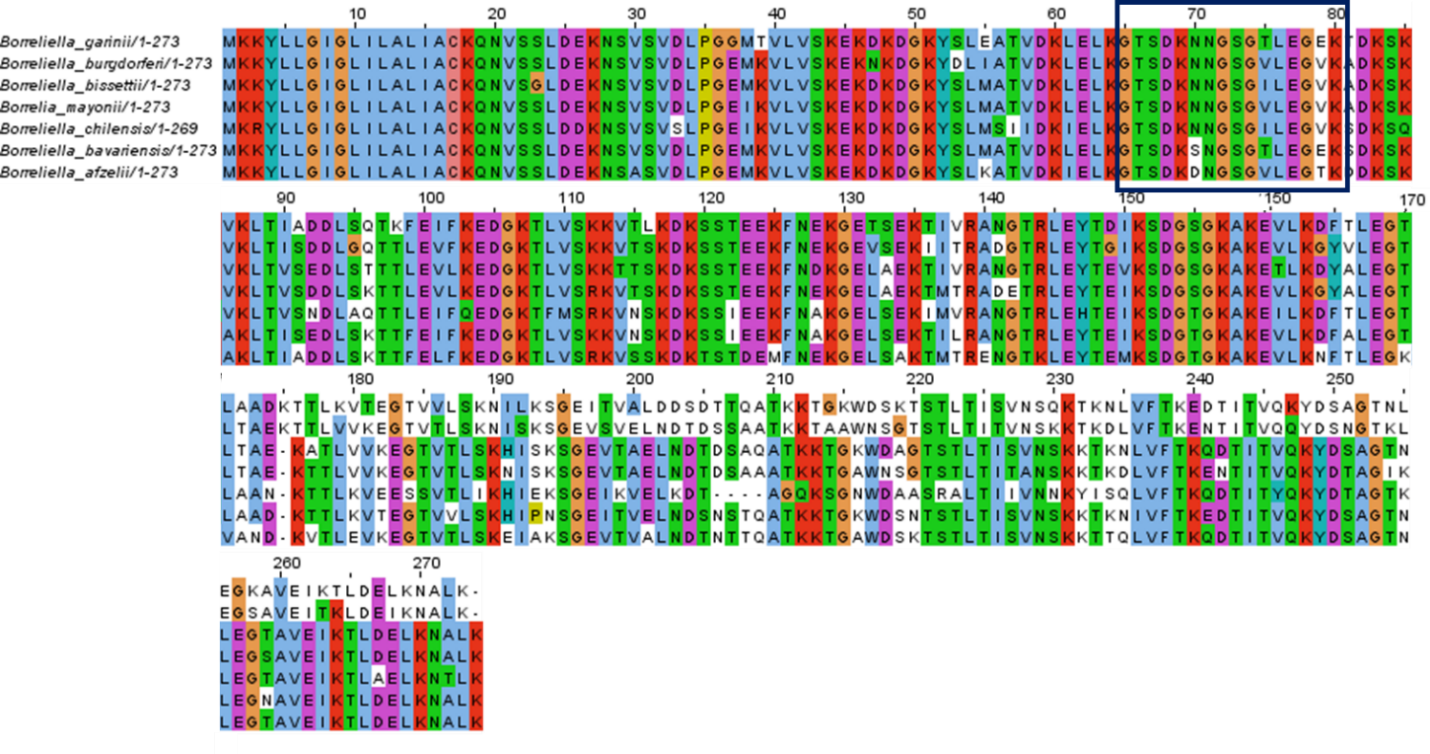

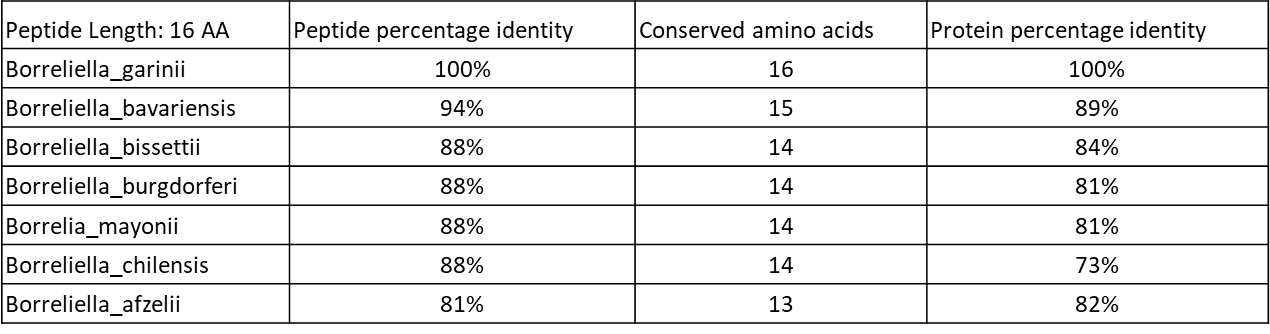


**Fig. S7**. Taxonomy analysis confirms protein database annotation and peptide attribution to an organism at the species level. (Top) Protein database annotation validation. Full length OspA protein from *Borrelia garinii* showed greater than 60% identity with OspA proteins from *Borrelia bavariensis, burgdorferi ss, bissetti, mayoni, chilensis,* and *afzelii*. (Bottom) Comparison of protein sequences shows that the peptide GTSDKSNGSGILEGEK found in the urine of a patient from central Europe has 100% match with OspA from *Borrelia garinii* and one mismatch with OspA from *Borrelia bavariensis*, both previously described in that geographic area.


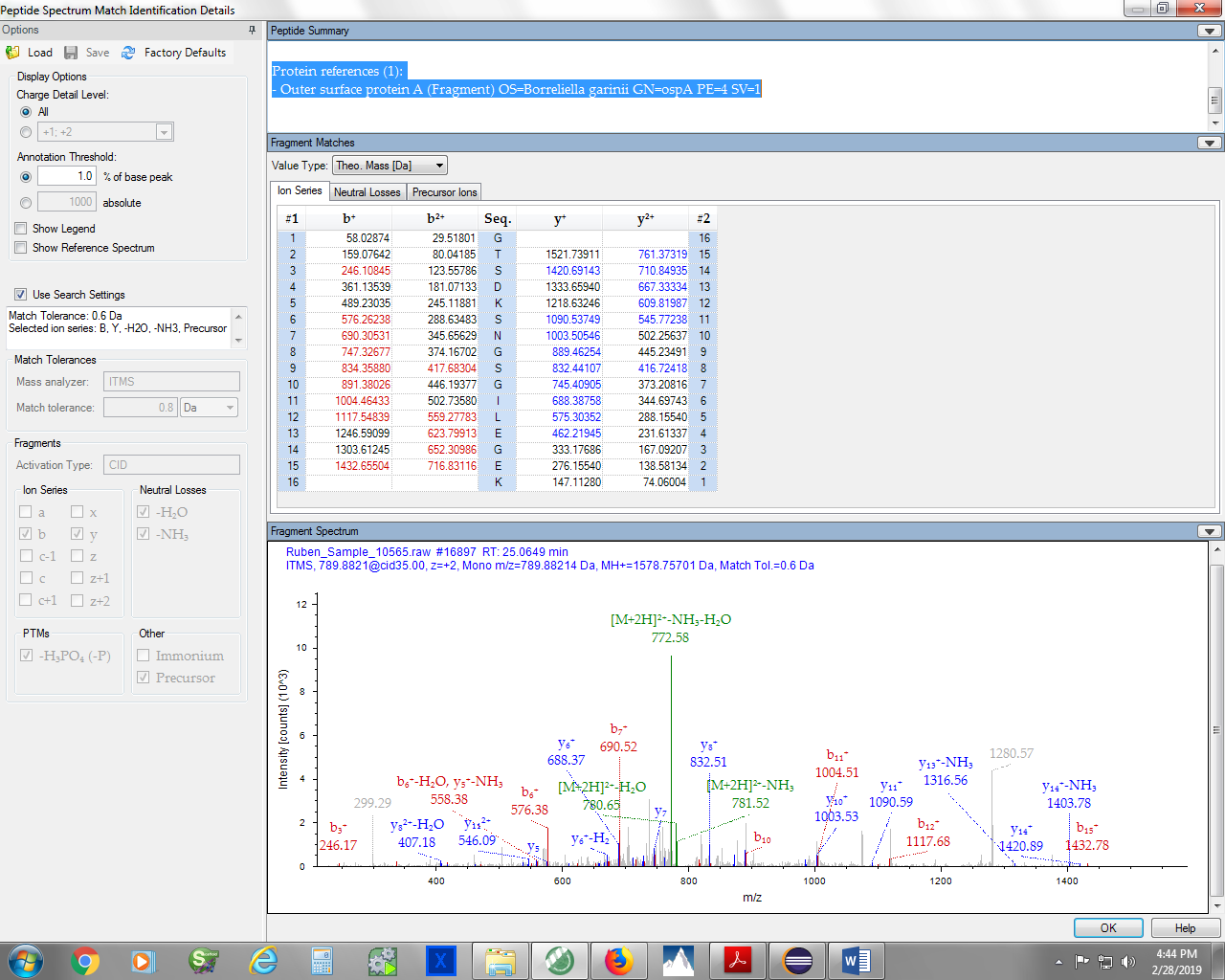

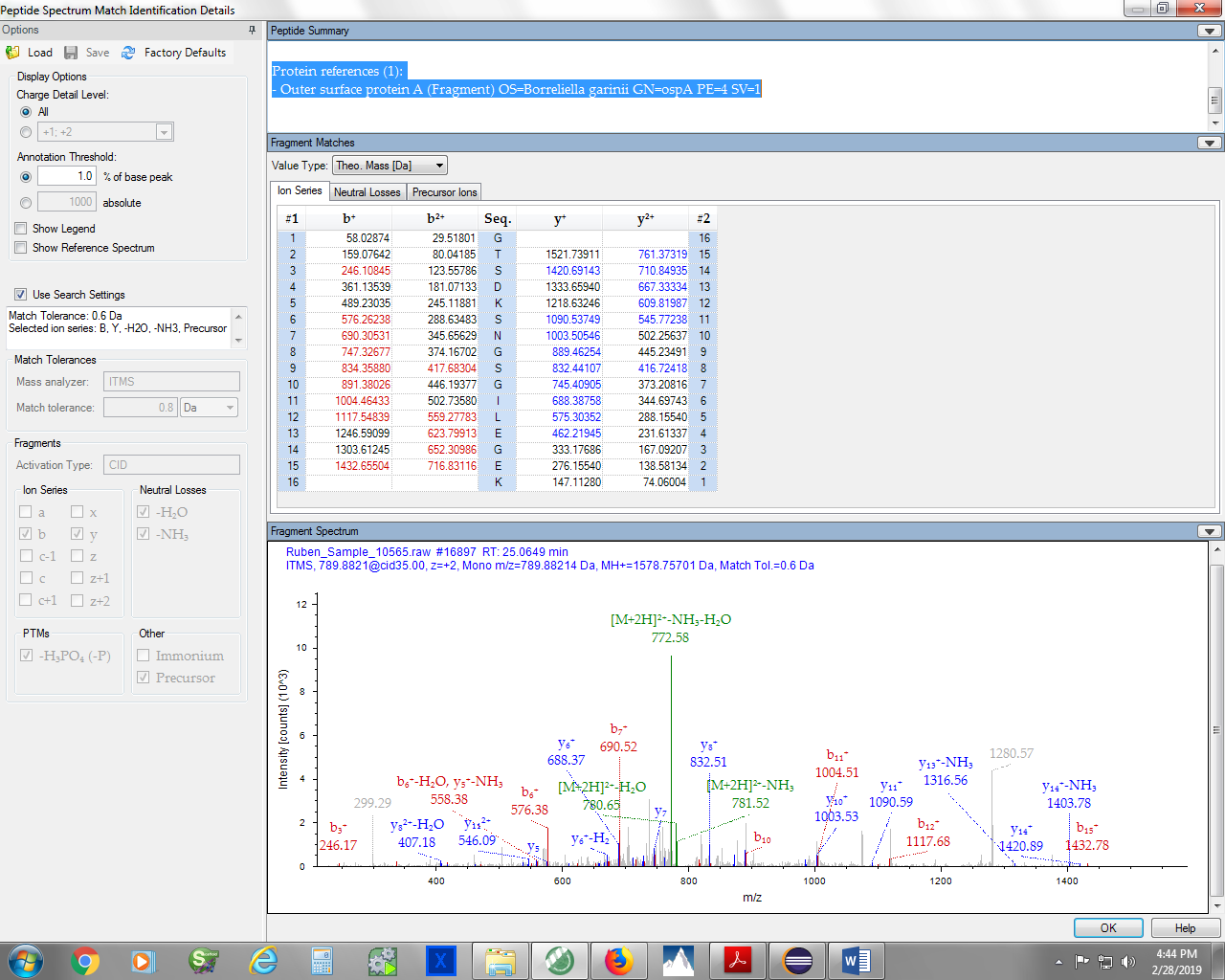


Sequence: **GTSDKSNGSGILEGEK**, Charge: +2, Monoisotopic m/z: 789.88214 Da (-1.78 mmu/-2.26 ppm), MH+: 1578.75701 Da, RT: 25.0649 min,

Identified with: Sequest HT (v1.17); XCorr:2.66, Percolator q-Value:0.000697, Percolator PEP:0.0223, Ions matched by search engine: 0/0

Fragment match tolerance used for search: 0.6 Da

Fragments used for search: b; b-H₂O; b-NH₃; y; y-H₂O; y-NH₃

Protein references:

- **Outer surface protein A** (Fragment) OS=Borrelia garinii GN=ospA PE=4 SV=1

**Fig. S8**. LC-MS/MS CID mass spectrum of an OspA-derived, tryptic peptide captured by affinity hydrogel nanoparticles and detected in the urine of non acute patiens.


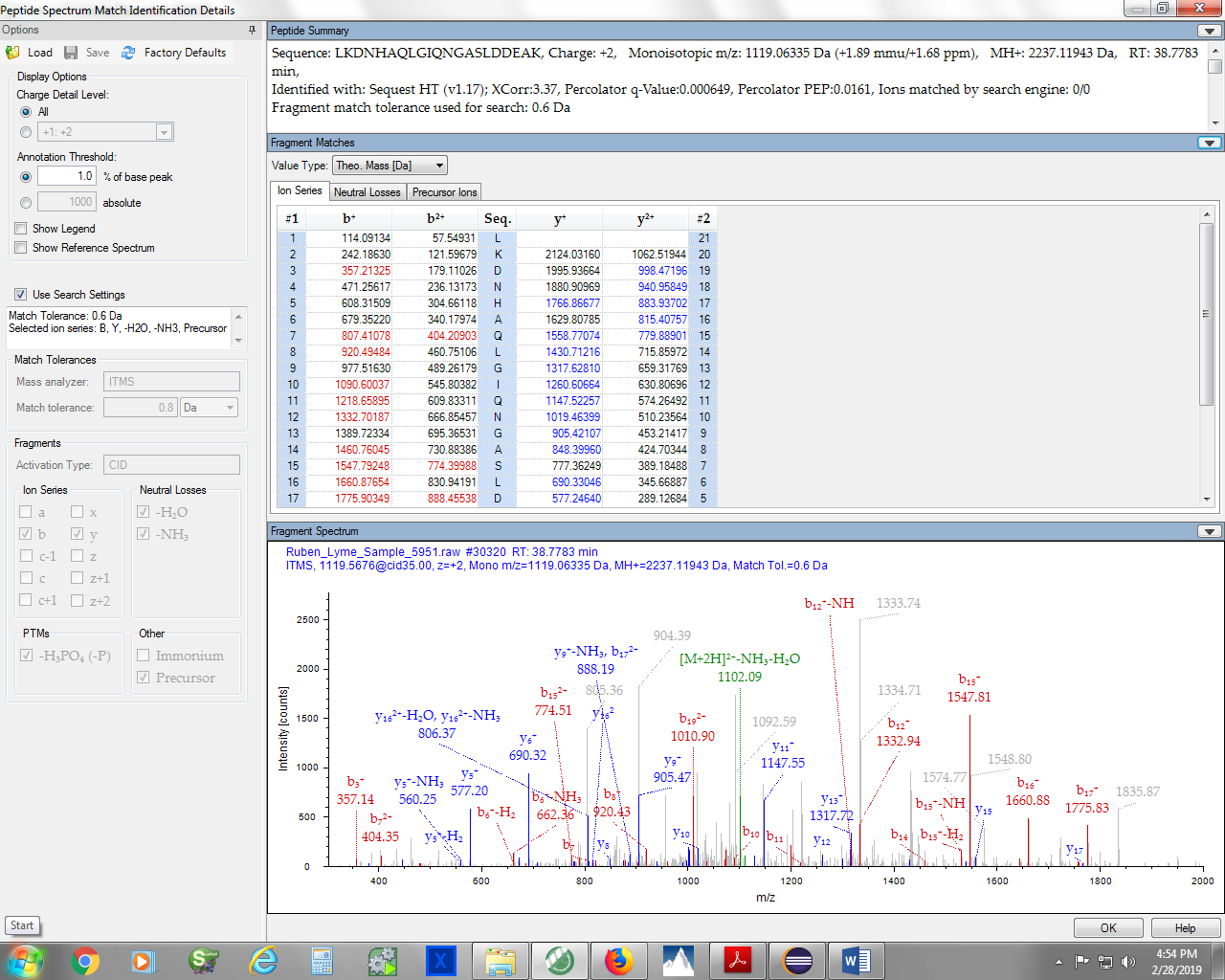


Sequence: **LKDNHAQLGIQNGASLDDEAK**, Charge: +2, Monoisotopic m/z: 1119.06335 Da (+1.89 mmu/+1.68 ppm), MH+: 2237.11943 Da, RT: 38.7783 min,

Identified with: Sequest HT (v1.17); XCorr:3.37, Percolator q-Value:0.000649, Percolator PEP:0.0161, Ions matched by search engine: 0/0

Fragment match tolerance used for search: 0.6 Da

Fragments used for search: b; b-H₂O; b-NH₃; y; y-H₂O; y-NH₃

Protein reference:

- **Outer surface protein C** (Fragment) OS=Borrelia burgdorferi GN=ospC PE=4 SV=1


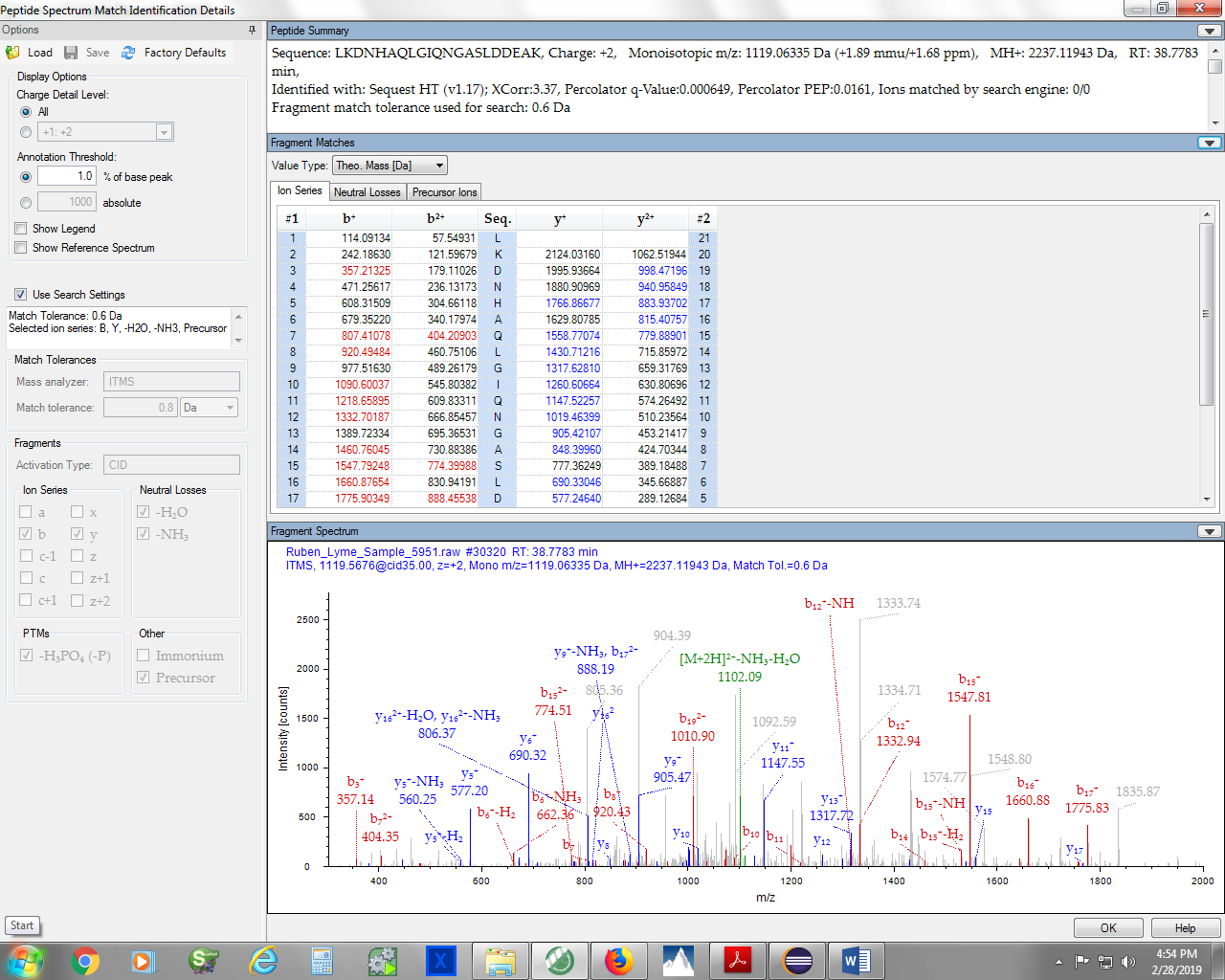


**Fig. S9**. LC-MS/MS CID mass spectrum of an OspC-derived, tryptic peptide captured by affinity hydrogel nanoparticles and detected in the urine of non acute patients.
.


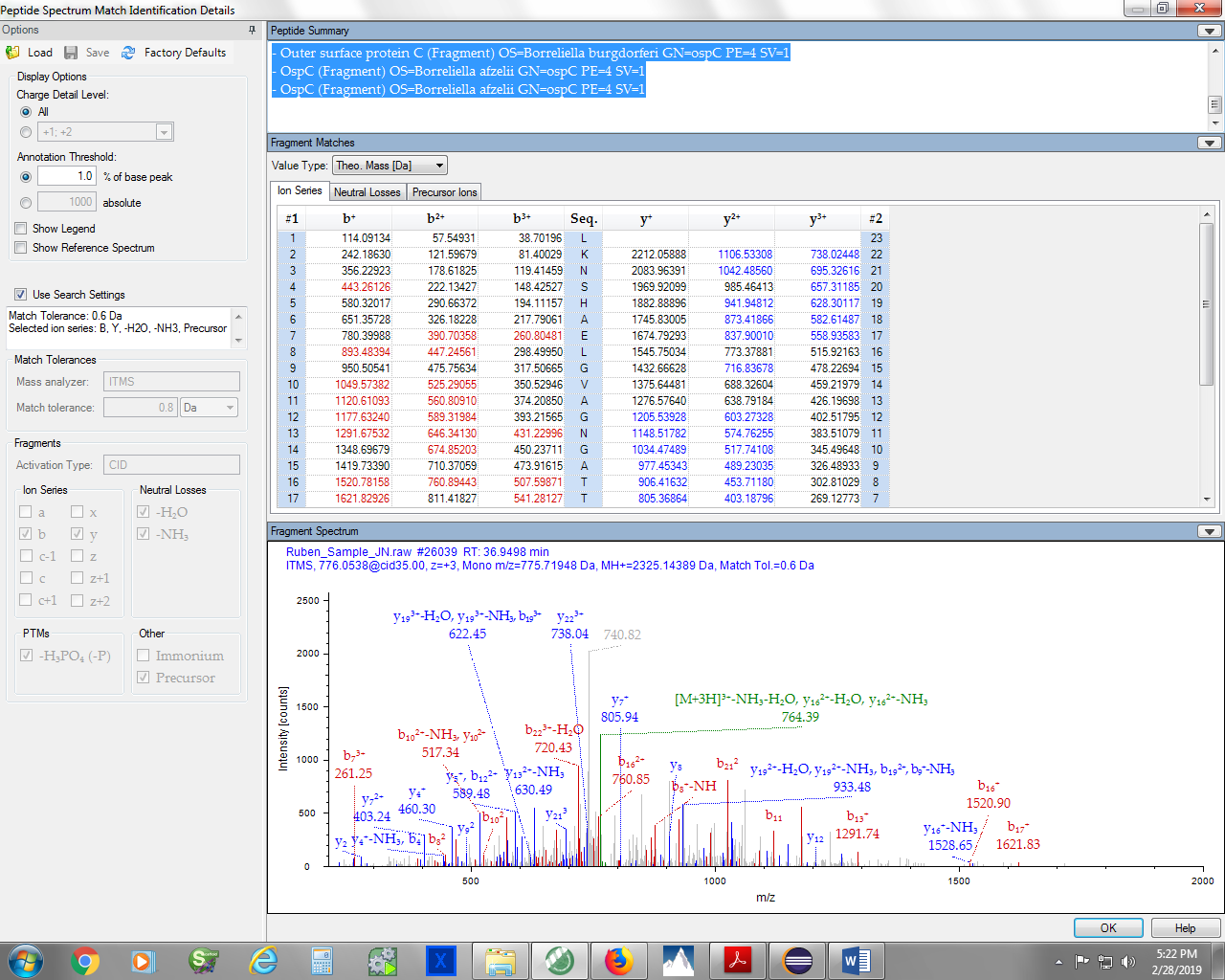

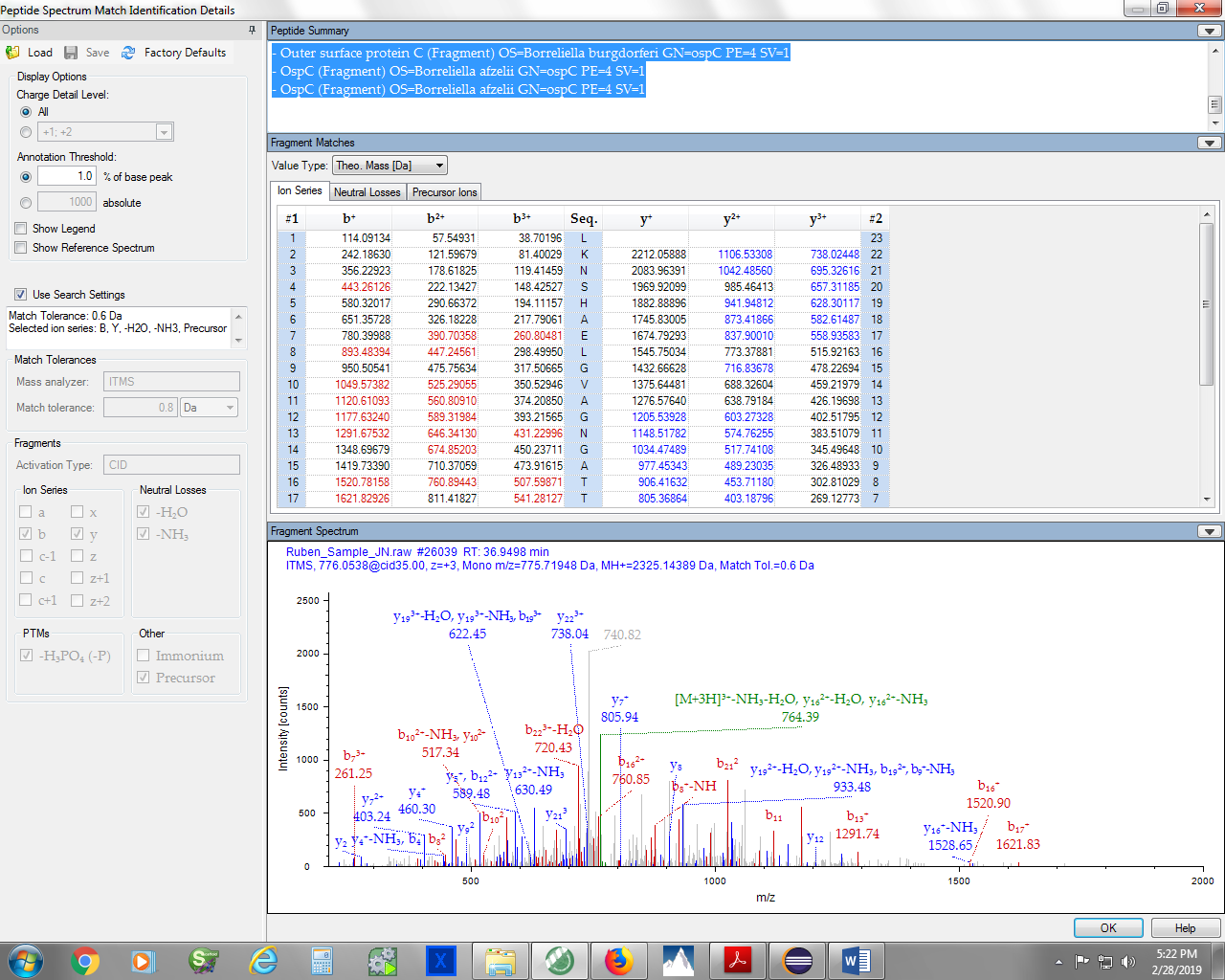


Sequence: **LKNSHAELGVAGNGATTDENAQK**, Charge: +3, Monoisotopic m/z: 775.71948 Da (+0.32 mmu/+0.41 ppm), MH+: 2325.14389 Da, RT: 36.9498 min,

Identified with: Sequest HT (v1.17); XCorr:3.74, Percolator q-Value:0.00109, Percolator PEP:0.0201, Ions matched by search engine: 0/0

Fragment match tolerance used for search: 0.6 Da

Fragments used for search: b; b-H₂O; b-NH₃; y; y-H₂O; y-NH₃

Protein references:

- **Outer surface protein C** (Fragment) OS=Borrelia burgdorferi GN=ospC PE=4 SV=1

**Fig. S10**. LC-MS/MS CID mass spectrum of an OspC-derived, tryptic peptide captured by affinity hydrogel nanoparticles and detected in the urine of non acute patients.


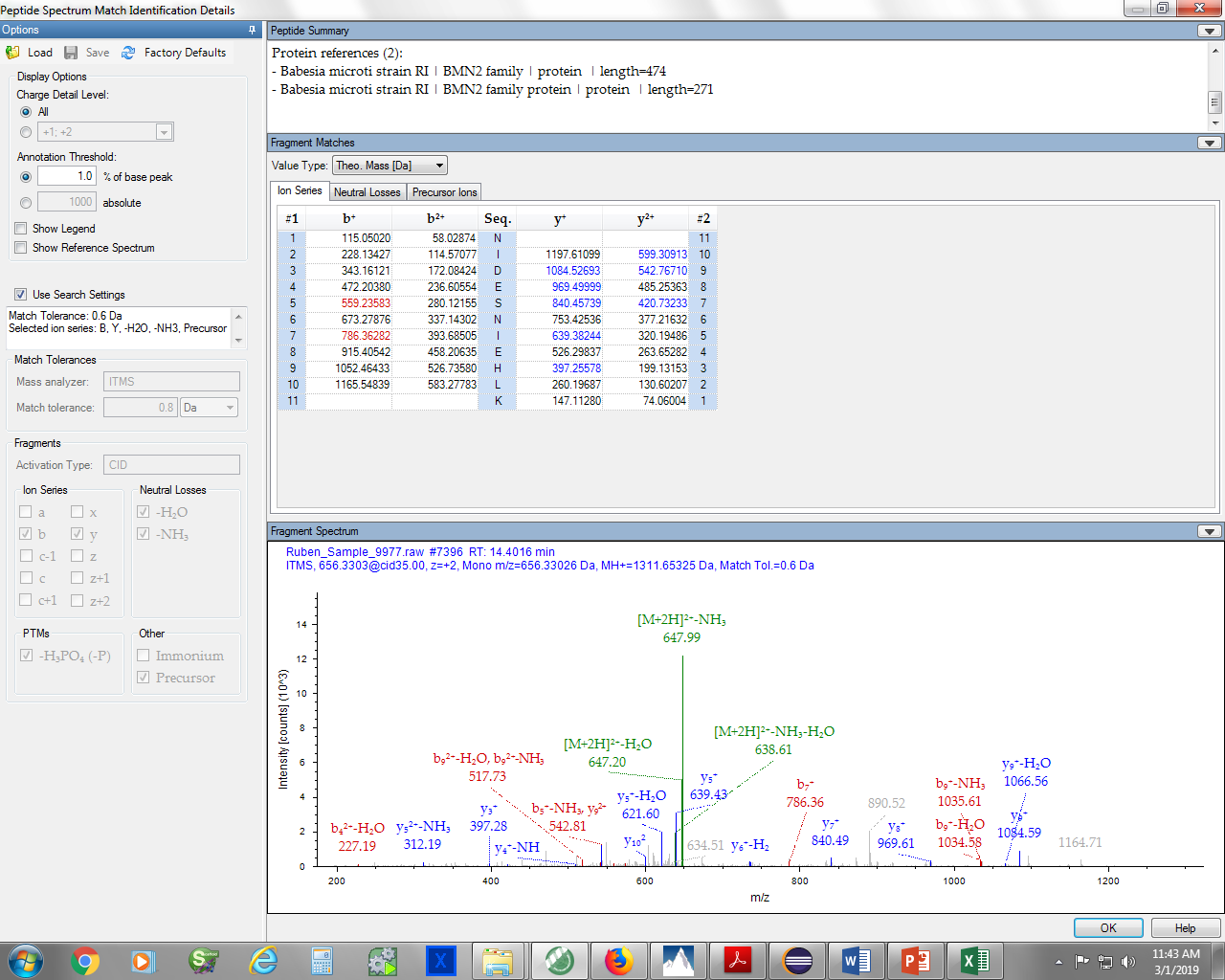

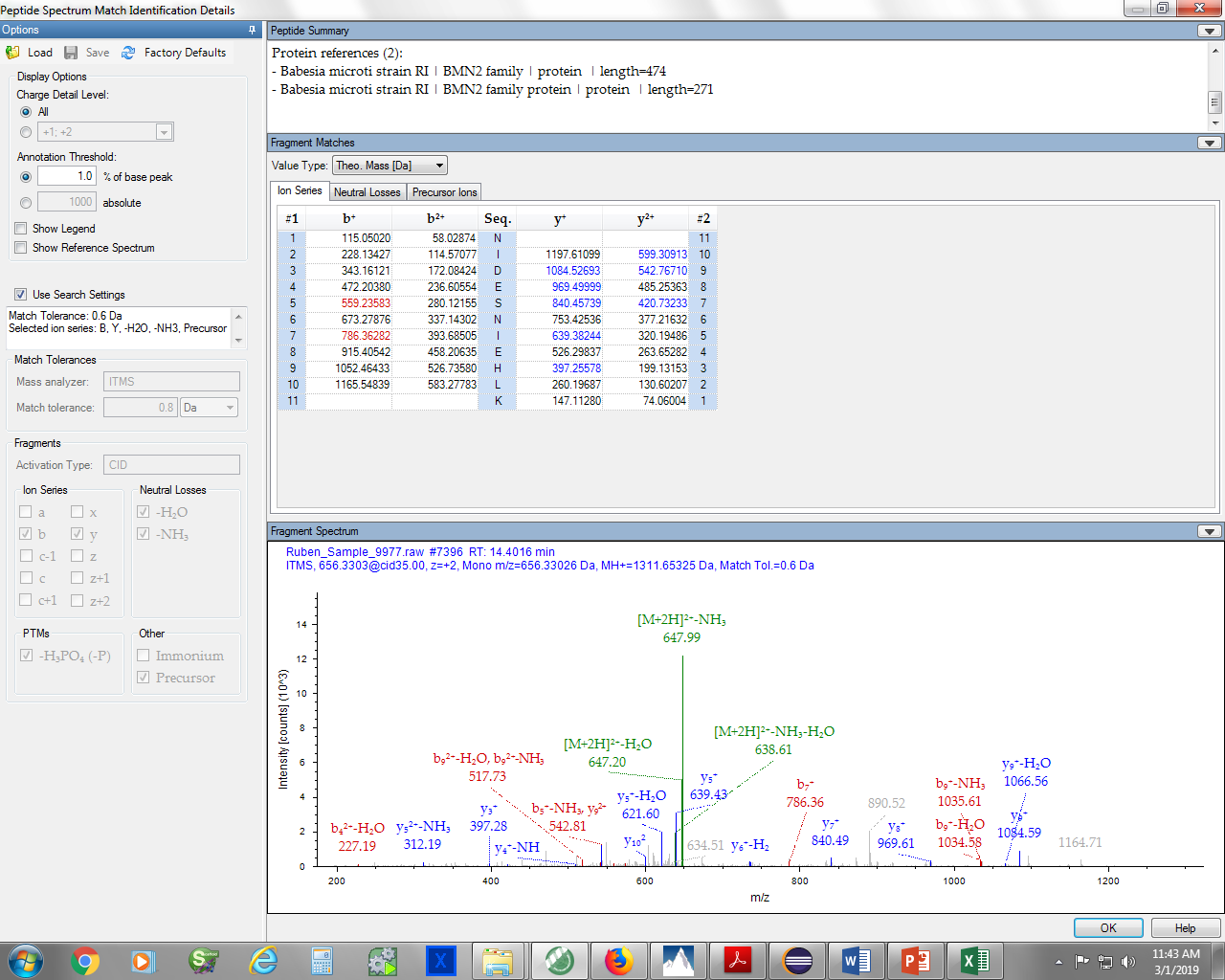


Sequence: **NIDESNIEHLK**, Charge: +2, Monoisotopic m/z: 656.33026 Da (-0.34 mmu/-0.51 ppm), MH+: 1311.65325 Da, RT: 14.4016 min,

Identified with: Sequest HT (v1.17); XCorr:2.42, Percolator q-Value:0.00358, Percolator PEP:0.0448, Ions matched by search engine: 0/0

Fragment match tolerance used for search: 0.6 Da

Fragments used for search: b; b-H₂O; b-NH₃; y; y-H₂O; y-NH₃

Protein reference:

- Babesia microti strain RI | **BMN2** family | protein | length=474

**Fig. S11**. LC-MS/MS CID mass spectrum of a tryptic peptide derived from Protein RecA that was captured by affinity hydrogel nanoparticles and detected in the urine of non-acute patients.


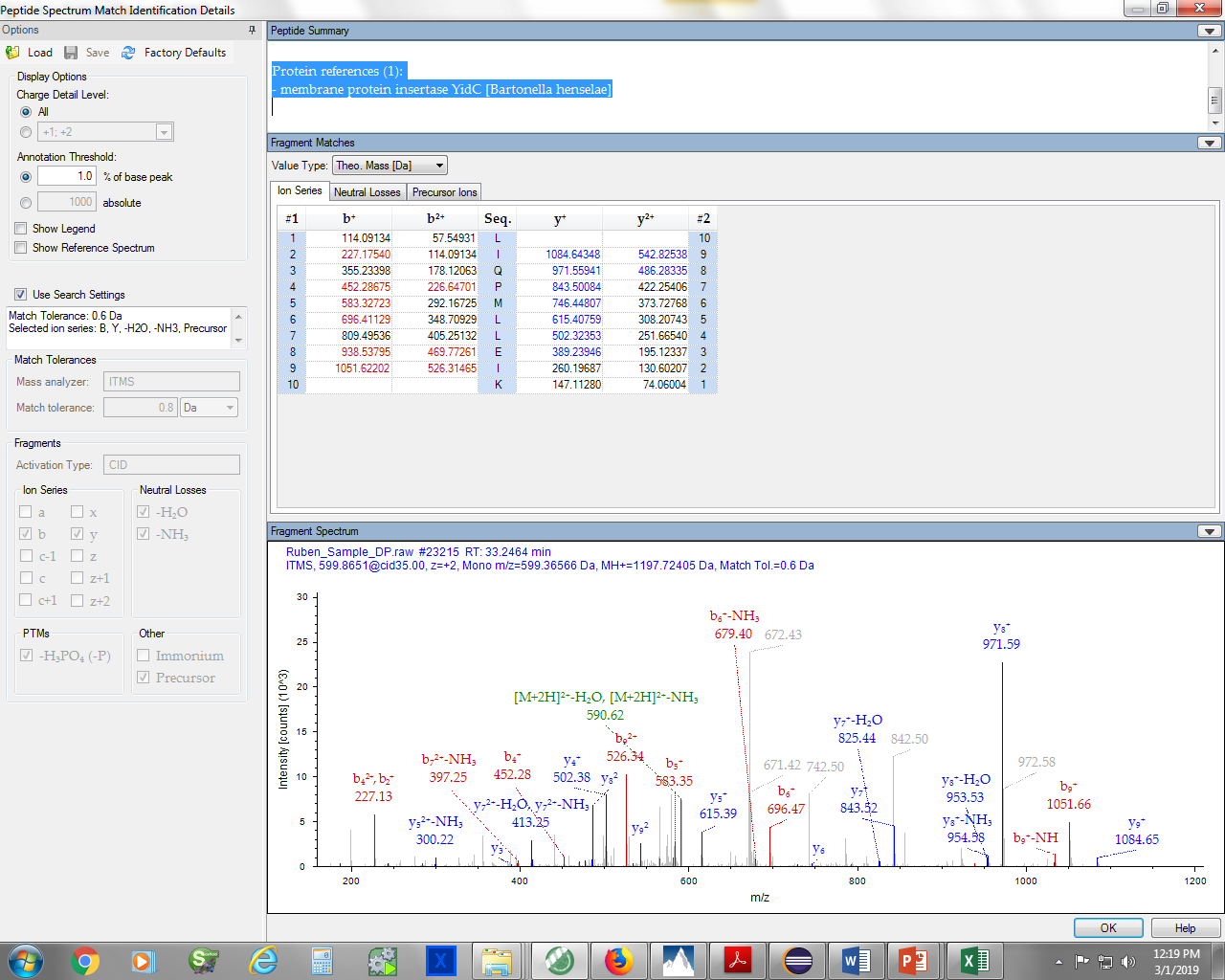

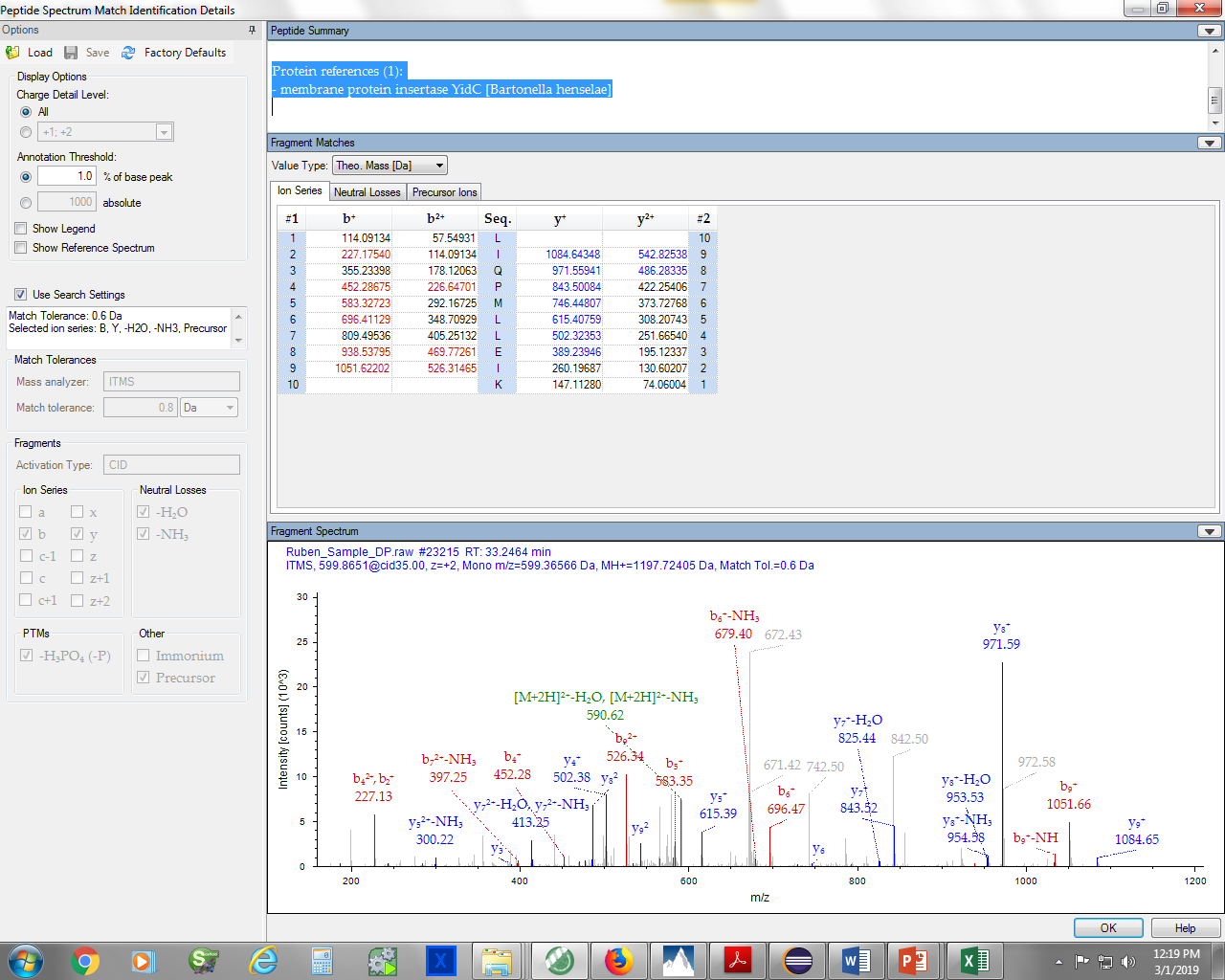


Sequence: **LIQPMLLEIK**, Charge: +2, Monoisotopic m/z: 599.36566 Da (-1.75 mmu/-2.92 ppm), MH+: 1197.72405 Da, RT: 33.2464 min,

Identified with: Sequest HT (v1.17); XCorr:2.63, Percolator q-Value:0.00285, Percolator PEP:0.0913, Ions matched by search engine: 0/0

Fragment match tolerance used for search: 0.6 Da

Fragments used for search: b; b-H₂O; b-NH₃; y; y-H₂O; y-NH₃

Protein reference:

- **membrane protein insertase YidC** [Bartonella henselae]

**Fig. S12**. LC-MS/MS CID mass spectrum of a tryptic peptide derived from membrane protein insertase YidC [*Bartonella henselae*] that was captured by affinity hydrogel nanoparticles and detected in the urine of non acute patients.


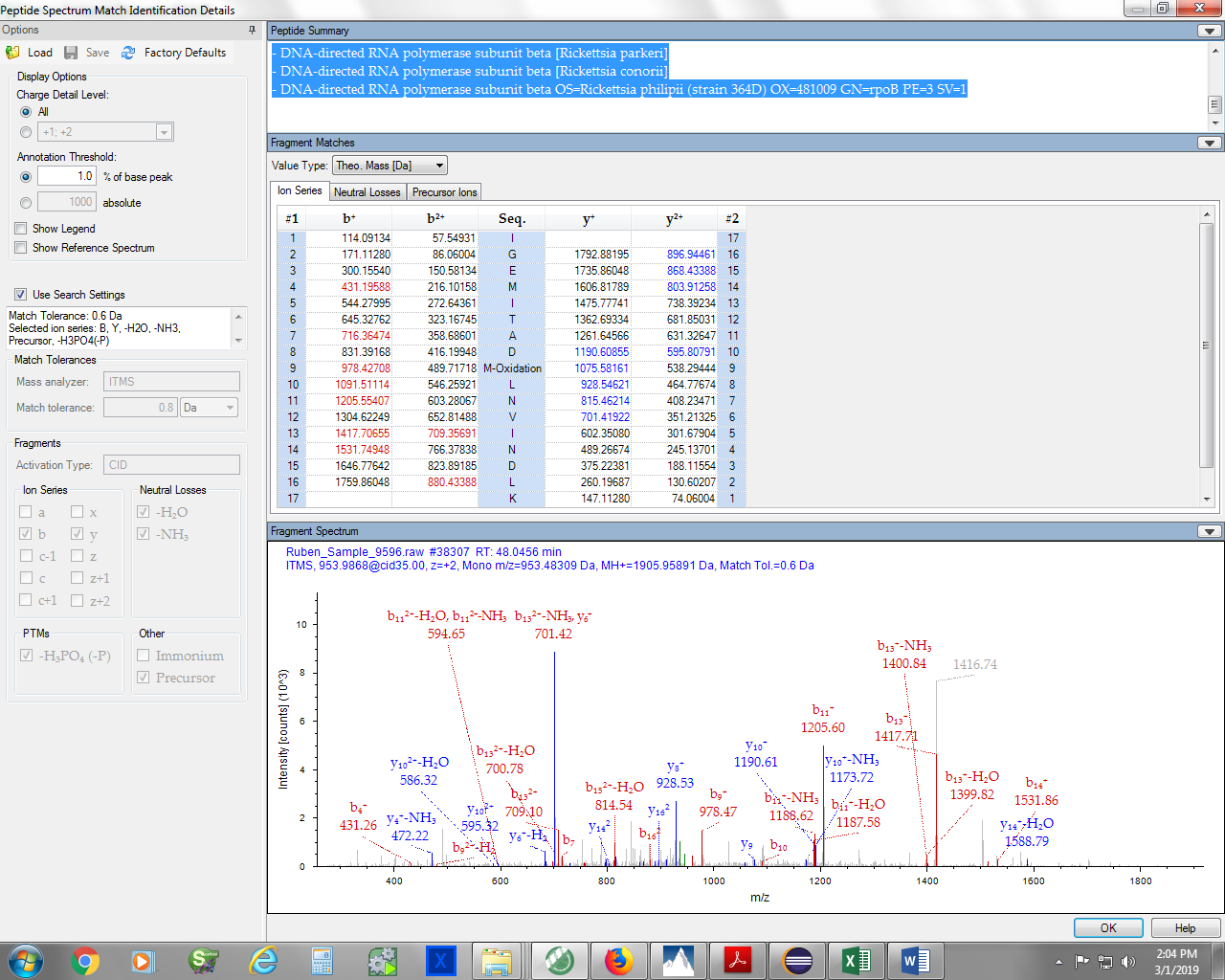

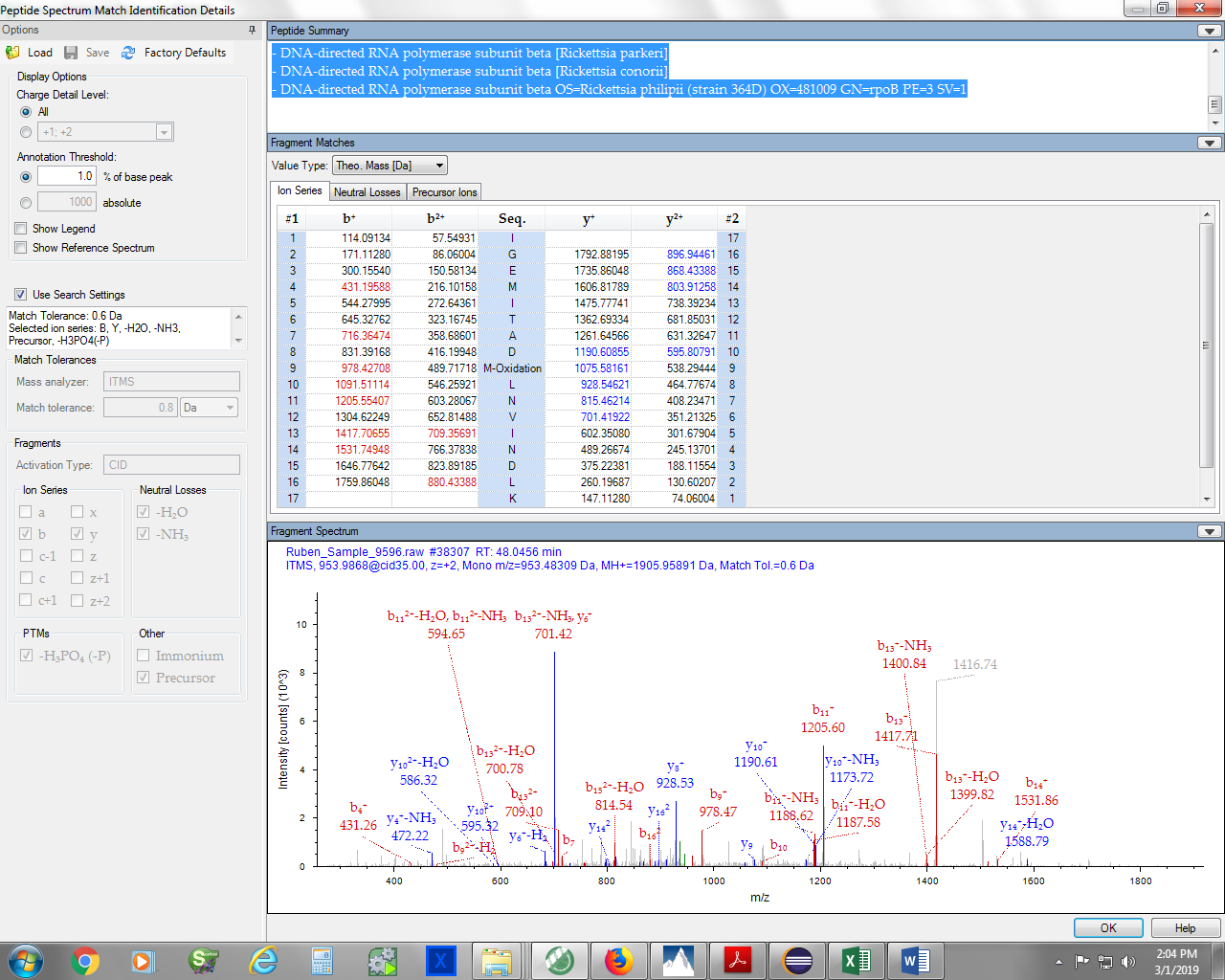


Sequence: **IGEMITADMLNVINDLK**, M9-Oxidation (15.99492 Da)

Charge: +2, Monoisotopic m/z: 953.48309 Da (-3.55 mmu/-3.73 ppm), MH+: 1905.95891 Da, RT: 48.0456 min,

Identified with: Sequest HT (v1.17); XCorr:3.33, Percolator q-Value:0.00502, Percolator PEP:0.0535, Ions matched by search engine: 0/0

Fragment match tolerance used for search: 0.6 Da

Fragments used for search: b; b-H₂O; b-NH₃; y; y-H₂O; y-NH₃

Protein references (3):

- **DNA-directed RNA polymerase subunit beta** [Rickettsia parkeri]

- **DNA-directed RNA polymerase subunit beta** [Rickettsia conorii]

- **DNA-directed RNA polymerase subunit beta** OS=Rickettsia philipii (strain 364D) OX=481009 GN=rpoB PE=3 SV=1

**Fig. S13**. LC-MS/MS CID mass spectrum of a tryptic peptide derived from DNA-directed RNA polymerase subunit beta [*Rickettsia parkeri, Rickettsia conorii, Rickettsia philipii*] that was captured by affinity hydrogel nanoparticles and detected in the urine of non acute patients.


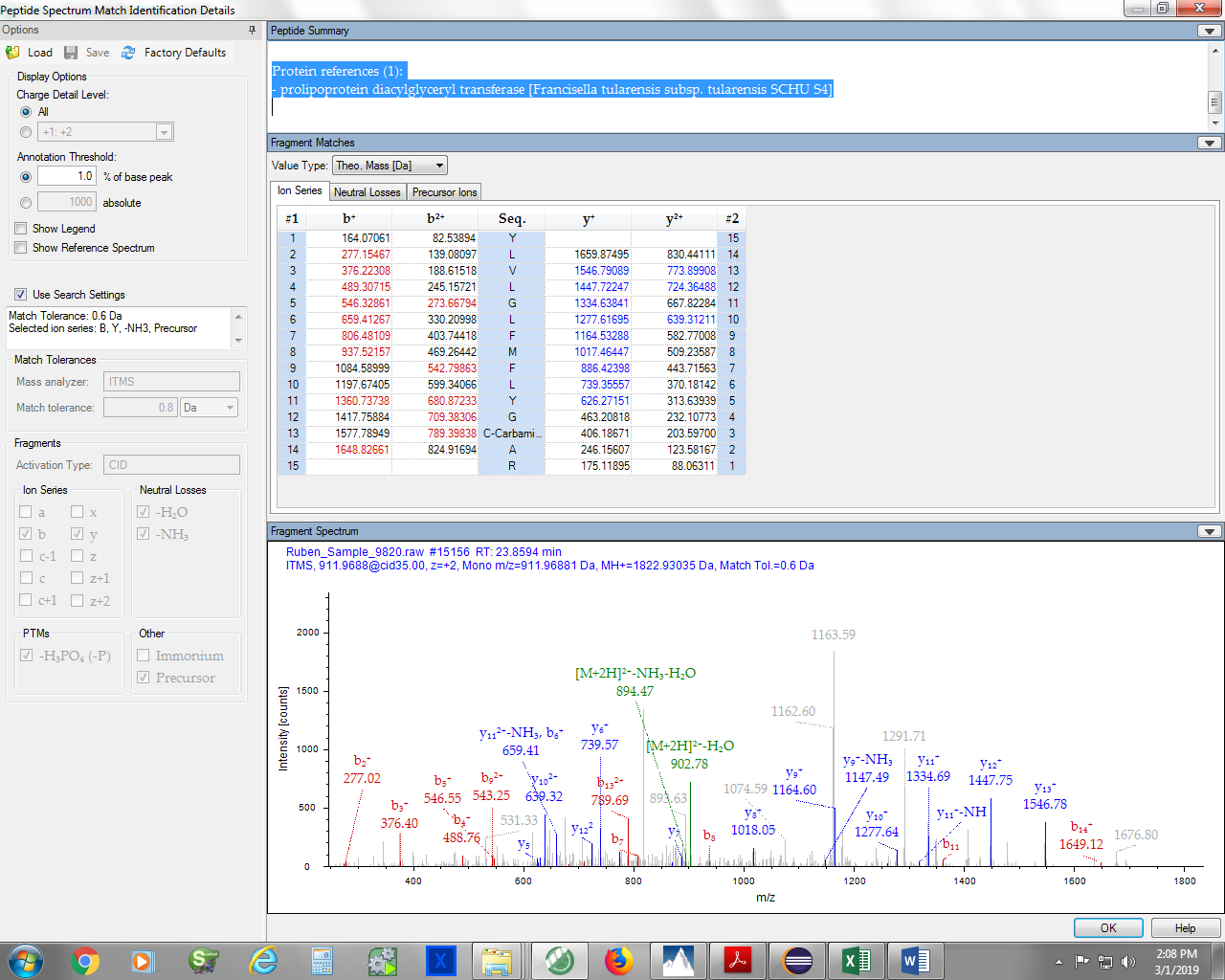

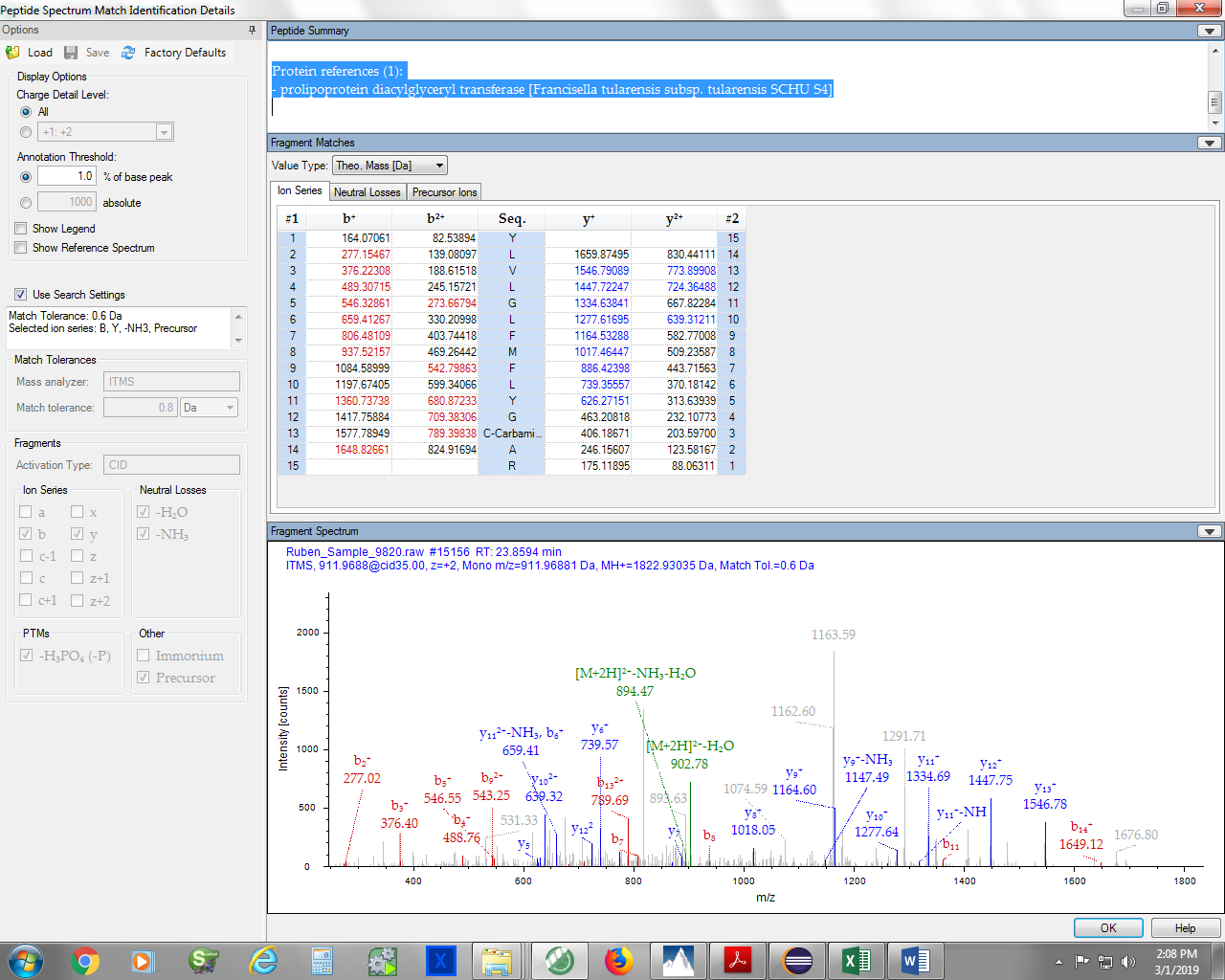


Sequence: **YLVLGLFMFLYGCAR**, C13-Carbamidomethyl (57.02146 Da)

Charge: +2, Monoisotopic m/z: 911.96881 Da (-3.97 mmu/-4.35 ppm), MH+: 1822.93035 Da, RT: 23.8594 min,

Identified with: Sequest HT (v1.17); XCorr:2.18, Percolator q-Value:0.006, Percolator PEP:0.0795, Ions matched by search engine: 0/0

Fragment match tolerance used for search: 0.6 Da

Fragments used for search: b; y; y-NH₃

Protein references (1):

- **prolipoprotein diacylglyceryl transferase** [Francisella tularensis subsp. tularensis SCHU S4]

**Fig. S14**. LC-MS/MS CID mass spectrum of a tryptic peptide derived from prolipoprotein diacylglyceryl transferase [*Francisella tularensis*] that was captured by affinity hydrogel nanoparticles and detected in the urine of non acute patients.


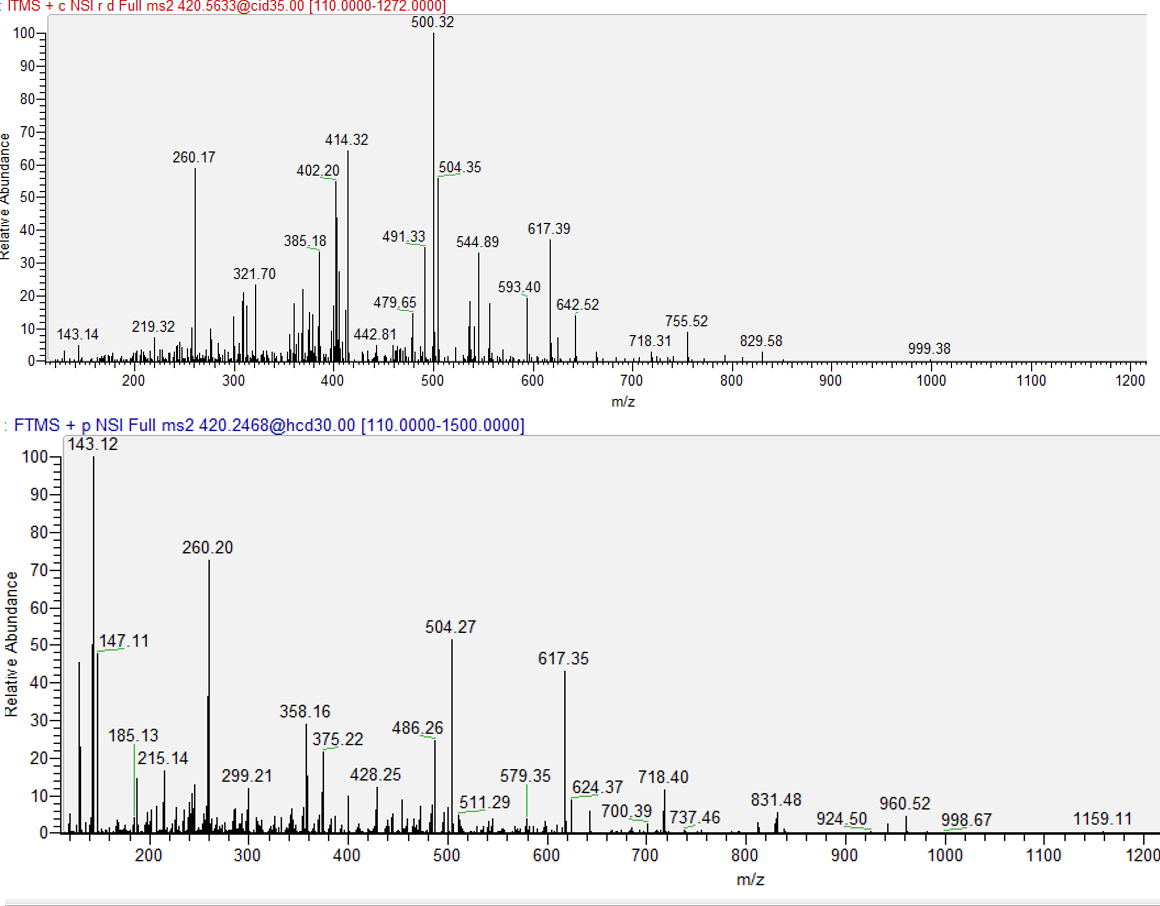


**Fig. S15**. Comparison between discovery MS/MS spectrum (above) and PRM showing entire MS/MS spectrum (below) for peptide AVEIKTLDELK (m/z 420.2468 ^3+^; MH+ 1258.726). While some variability between the spectra is expected since the PRM and discovery approaches use different fragmentation and mass analyzers, the presence of several matching ions of high intensity (m/z: 143.12, 260.20, 504.32, 617.39, 718.40) obtained with high mass accuracy (<2ppm) verifies the identity of this peptide.


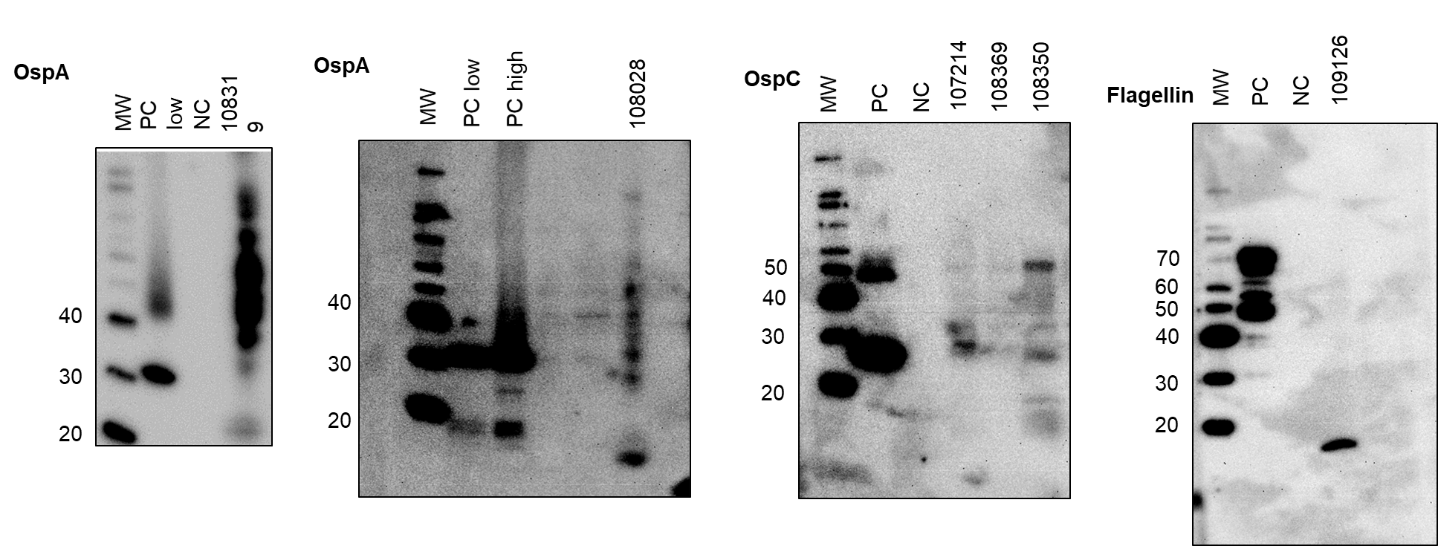


**Fig. S16.** Original, uncropped images of western blot membranes shown in Figure 5.
